# Supplementary material for: Trans-species polymorphism at antimicrobial innate immunity cathelicidin genes of Atlantic cod and related species
Source: PeerJ. 2015 May 21;3:e976. doi: 10.7717/peerj.976 (PMC4451034; doi:10.7717/peerj.976)
Supplement: Supplemental Information 1 — All the supplemental figures in one file. [file peerj-03-976-s001.pdf]

Supplementary data for the manuscript: Trans-species Polymorphism at Antimicrobial Innate Immunity Cathelicidin Genes of Atlantic cod and Related Species

Katrín Halldórsdóttir* and Einar Árnason

Institute of Life and Environmental Sciences, University of Iceland, Sturlugata 7, Reykjavík, Iceland

*Corresponding author. email: katrinhalldorsdottir@gmail.com

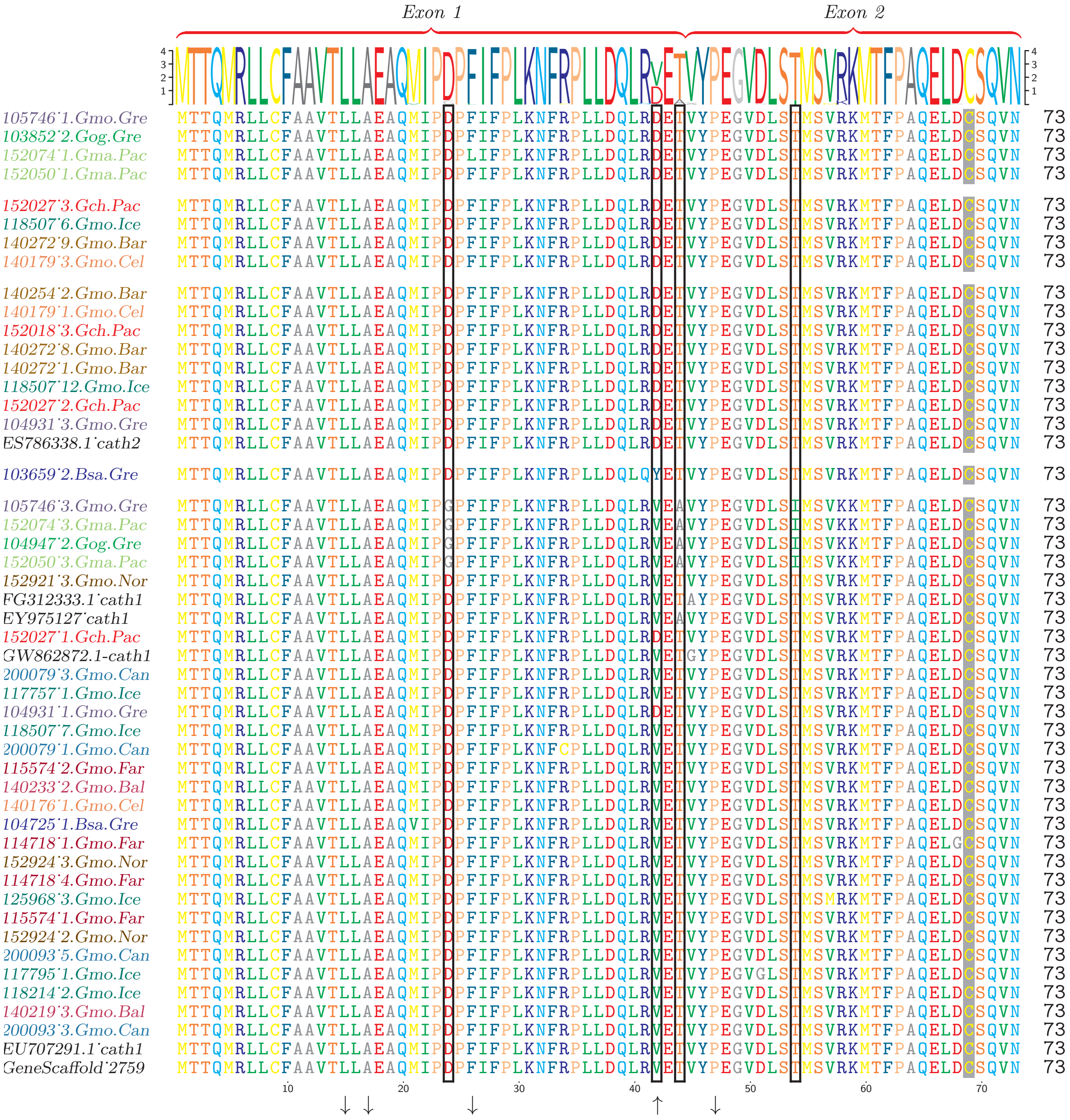
Exon 1
Exon 2
105746˙1.Gmo.Gre
103852˙2.Gog.Gre
152074˙1.Gma.Pac
152050˙1.Gma.Pac
152027˙3.Gch.Pac
118507˙6.Gmo.Ice
140272˙9.Gmo.Bar
140179˙3.Gmo.Cel
140254˙2.Gmo.Bar
140179˙1.Gmo.Cel
152018˙3.Gch.Pac
140272˙8.Gmo.Bar
140272˙1.Gmo.Bar
118507˙12.Gmo.Ice
152027˙2.Gch.Pac
104931˙3.Gmo.Gre
ES786338.1˙cath2
103659˙2.Bsa.Gre
105746˙3.Gmo.Gre
152074˙3.Gma.Pac
104947˙2.Gog.Gre
152050˙3.Gma.Pac
152921˙3.Gmo.Nor
FG312333.1˙cath1
EY975127˙cath1
152027˙1.Gch.Pac
GW862872.1-cath1
200079˙3.Gmo.Can
117757˙1.Gmo.Ice
104931˙1.Gmo.Gre
118507˙7.Gmo.Ice
200079˙1.Gmo.Can
115574˙2.Gmo.Far
140233˙2.Gmo.Bal
140176˙1.Gmo.Cel
104725˙1.Bsa.Gre
114718˙1.Gmo.Far
152924˙3.Gmo.Nor
114718˙4.Gmo.Far
125968˙3.Gmo.Ice
115574˙1.Gmo.Far
152924˙2.Gmo.Nor
200093˙5.Gmo.Can
117795˙1.Gmo.Ice
118214˙2.Gmo.Ice
140219˙3.Gmo.Bal
200093˙3.Gmo.Can
EU707291.1˙cath1
GeneScaffold˙2759
10
20
30
40
50
60
70

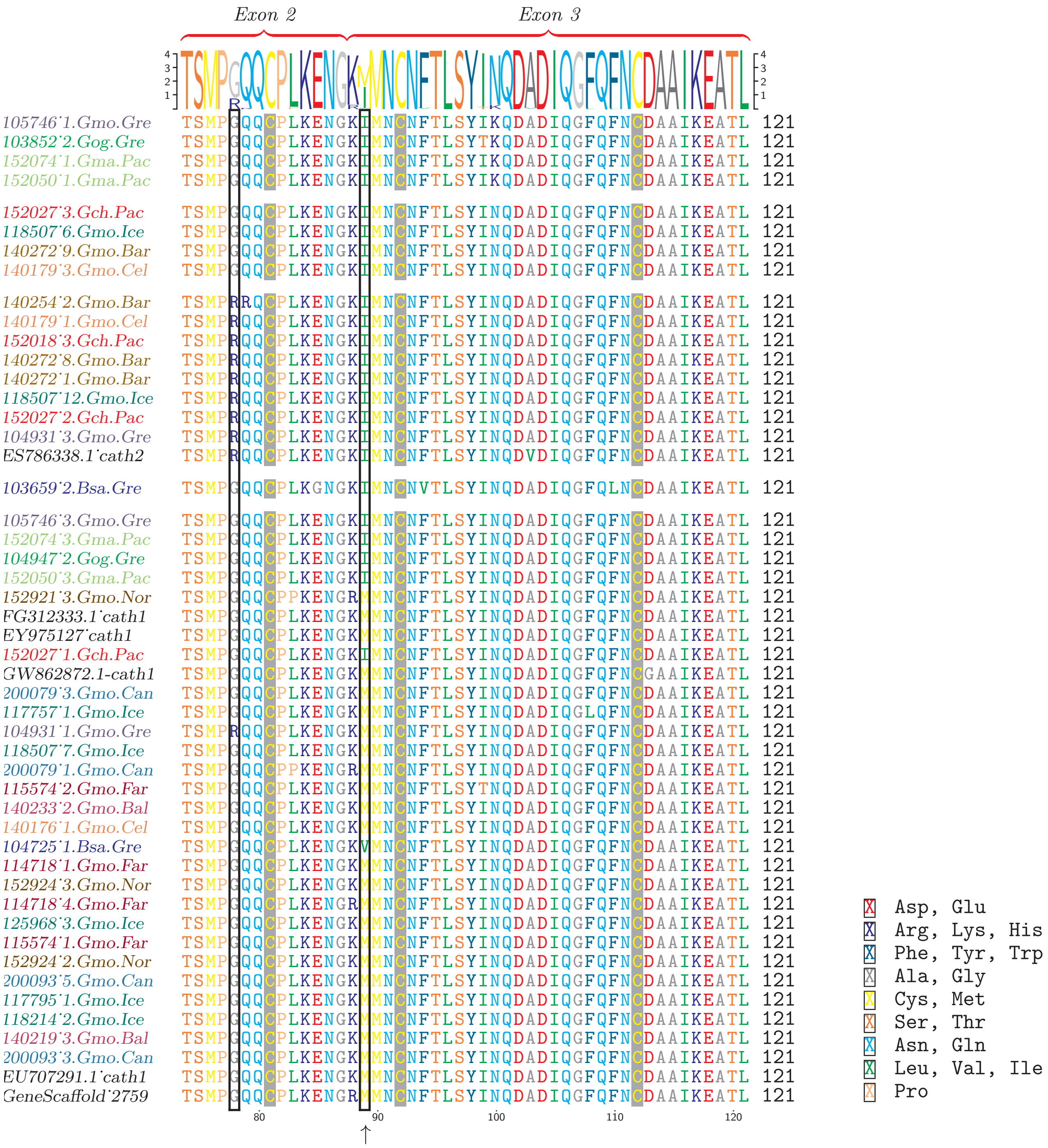

Figure S1. Alignment of amino acid sequences of exons 1, 2, and 3 combined, the conserved part of cathelicidin, from clones of various individuals of Atlantic cod and four closely related taxa. Highly polymorphic sites are boxed. The four conserved cysteine residues characterizing cathelicidin are shaded. Up arrows represent positively selected sites and down arrows negatively selected sites from Tables 1 and 2. Bsa.Gre (*Boreogadus saida*), Gch.Pac (*Gadus chalcogrammus*), Gma.Pac (*Gadus macrocephalus*), Gog.Gre (*Gadus ogac*) and Gmo (*Gadus morhua*) from various locations; Iceland (Gmo.Ice), Greenland (Gmo.Gre), Barents Sea (Gmo.Bar), Celtic Sea (Gmo.Cel), Baltic Sea (Gmo.Bal), Norway (Gmo.Nor), Faroe Islands (Gmo.Far), Canada (Gmo.Can).

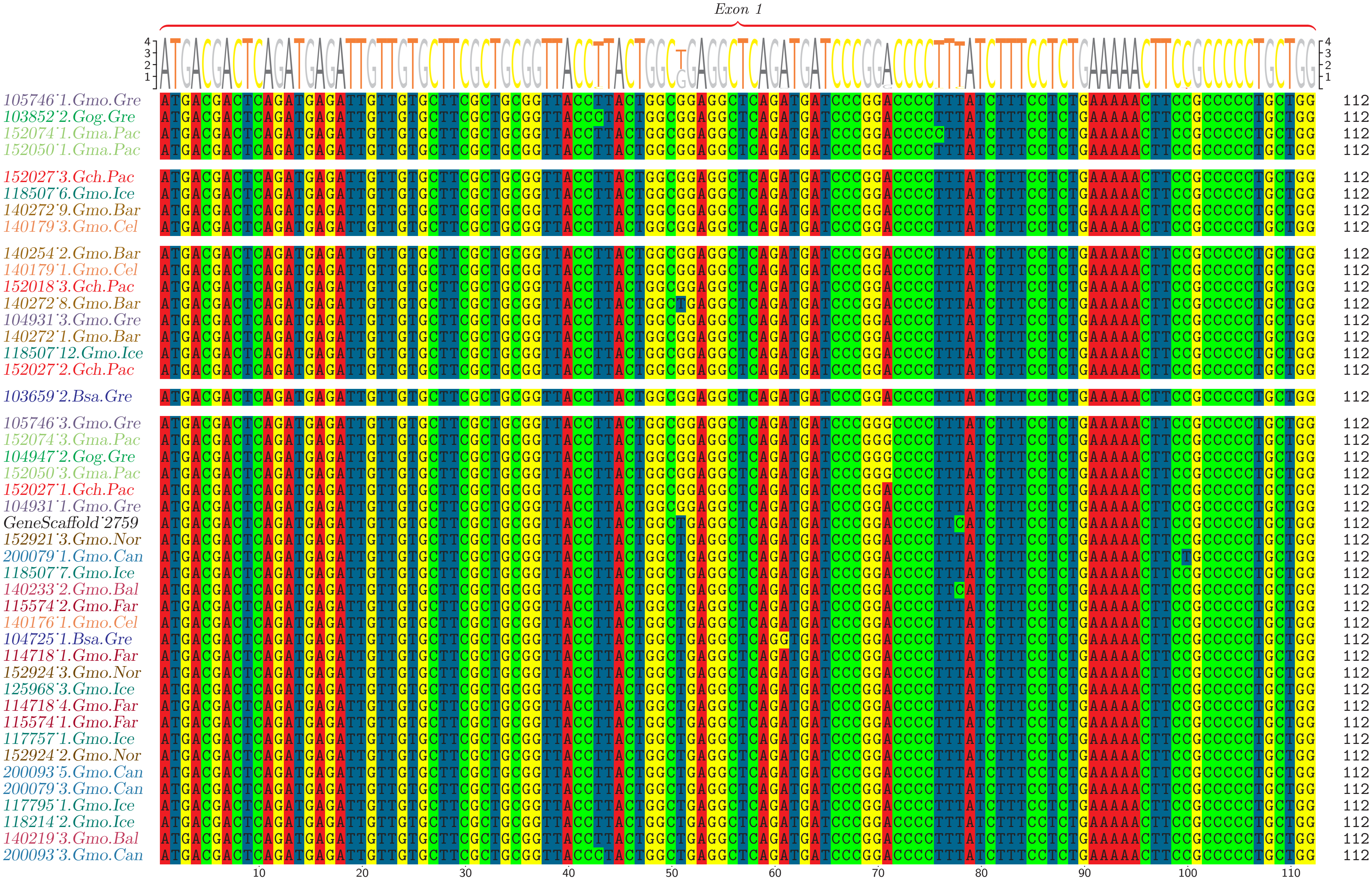

Exon 1
105746˙1.Gmo.Gre
103852˙2.Gog.Gre
152074˙1.Gma.Pac
152050˙1.Gma.Pac
152027˙3.Gch.Pac
118507˙6.Gmo.Ice
140272˙9.Gmo.Bar
140179˙3.Gmo.Cel
140254˙2.Gmo.Bar
140179˙1.Gmo.Cel
152018˙3.Gch.Pac
140272˙8.Gmo.Bar
104931˙3.Gmo.Gre
140272˙1.Gmo.Bar
118507˙12.Gmo.Ice
152027˙2.Gch.Pac
103659˙2.Bsa.Gre
105746˙3.Gmo.Gre
152074˙3.Gma.Pac
104947˙2.Gog.Gre
152050˙3.Gma.Pac
152027˙1.Gch.Pac
104931˙1.Gmo.Gre
GeneScaffold˙2759
152921˙3.Gmo.Nor
200079˙1.Gmo.Can
118507˙7.Gmo.Ice
140233˙2.Gmo.Bal
115574˙2.Gmo.Far
140176˙1.Gmo.Cel
104725˙1.Bsa.Gre
114718˙1.Gmo.Far
152924˙3.Gmo.Nor
125968˙3.Gmo.Ice
114718˙4.Gmo.Far
115574˙1.Gmo.Far
117757˙1.Gmo.Ice
152924˙2.Gmo.Nor
200093˙5.Gmo.Can
200079˙3.Gmo.Can
117795˙1.Gmo.Ice
118214˙2.Gmo.Ice
140219˙3.Gmo.Bal
200093˙3.Gmo.Can

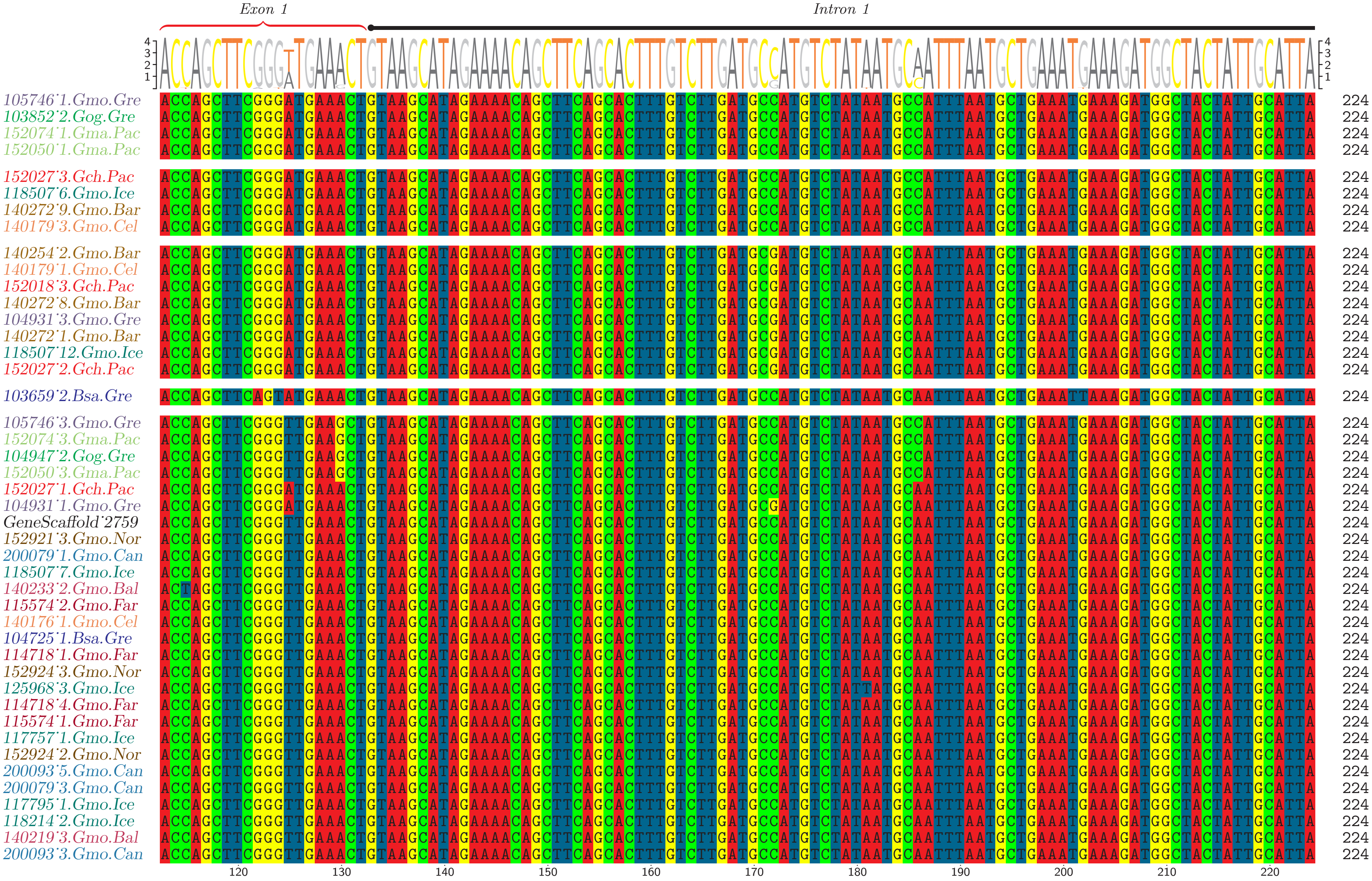

Exon 1
Intron 1
105746˙1.Gmo.Gre
103852˙2.Gog.Gre
152074˙1.Gma.Pac
152050˙1.Gma.Pac
152027˙3.Gch.Pac
118507˙6.Gmo.Ice
140272˙9.Gmo.Bar
140179˙3.Gmo.Cel
140254˙2.Gmo.Bar
140179˙1.Gmo.Cel
152018˙3.Gch.Pac
140272˙8.Gmo.Bar
104931˙3.Gmo.Gre
140272˙1.Gmo.Bar
118507˙12.Gmo.Ice
152027˙2.Gch.Pac
103659˙2.Bsa.Gre
105746˙3.Gmo.Gre
152074˙3.Gma.Pac
104947˙2.Gog.Gre
152050˙3.Gma.Pac
152027˙1.Gch.Pac
104931˙1.Gmo.Gre
GeneScaffold˙2759
152921˙3.Gmo.Nor
200079˙1.Gmo.Can
118507˙7.Gmo.Ice
140233˙2.Gmo.Bal
115574˙2.Gmo.Far
140176˙1.Gmo.Cel
104725˙1.Bsa.Gre
114718˙1.Gmo.Far
152924˙3.Gmo.Nor
125968˙3.Gmo.Ice
114718˙4.Gmo.Far
115574˙1.Gmo.Far
117757˙1.Gmo.Ice
152924˙2.Gmo.Nor
200093˙5.Gmo.Can
200079˙3.Gmo.Can
117795˙1.Gmo.Ice
118214˙2.Gmo.Ice
140219˙3.Gmo.Bal
200093˙3.Gmo.Can
224
120
130
140
150
160
170
180
190
200
210
220

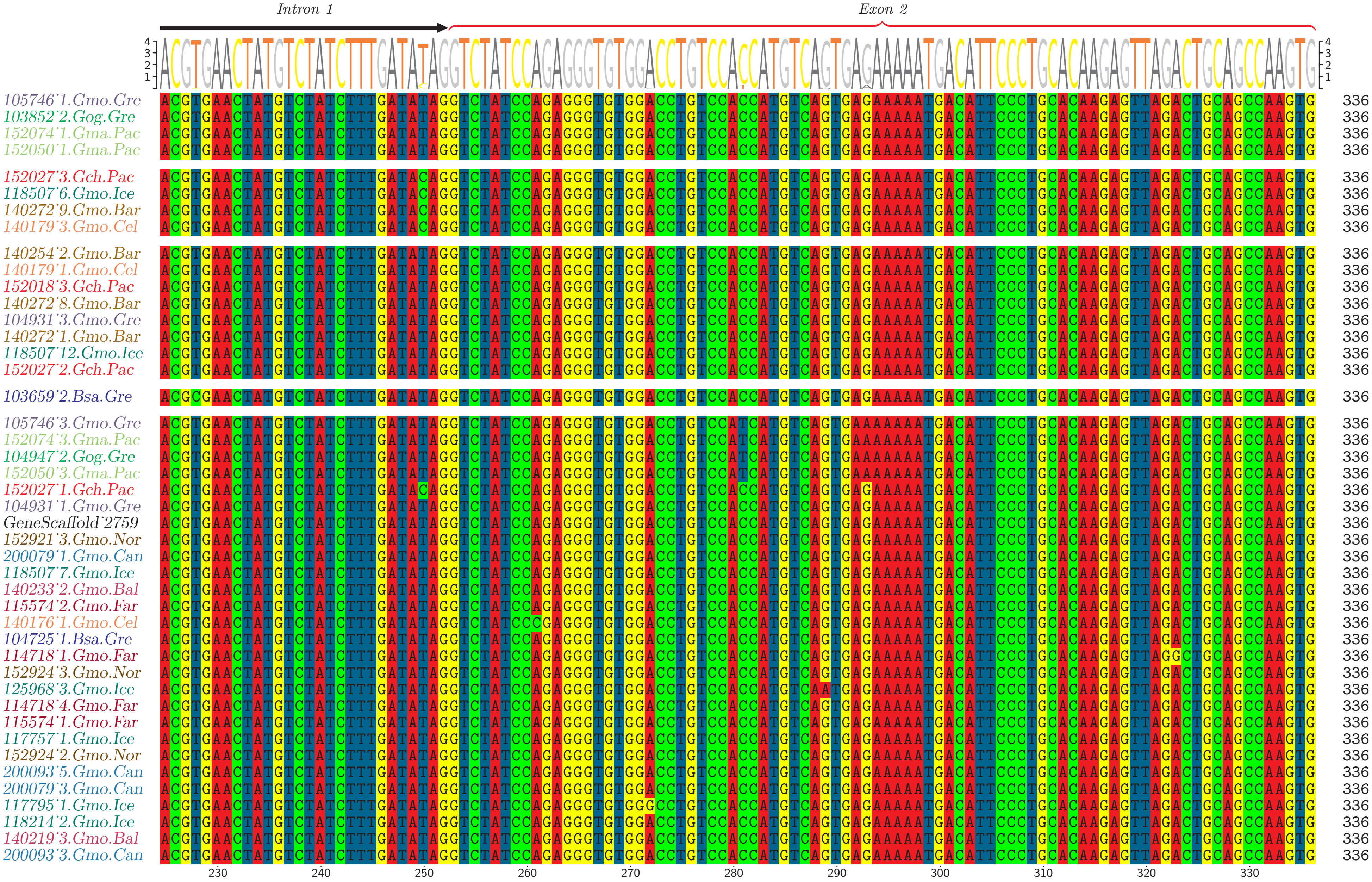

Intron 1
Exon 2
105746˙1.Gmo.Gre
103852˙2.Gog.Gre
152074˙1.Gma.Pac
152050˙1.Gma.Pac
152027˙3.Gch.Pac
118507˙6.Gmo.Ice
140272˙9.Gmo.Bar
140179˙3.Gmo.Cel
140254˙2.Gmo.Bar
140179˙1.Gmo.Cel
152018˙3.Gch.Pac
140272˙8.Gmo.Bar
104931˙3.Gmo.Gre
140272˙1.Gmo.Bar
118507˙12.Gmo.Ice
152027˙2.Gch.Pac
103659˙2.Bsa.Gre
105746˙3.Gmo.Gre
152074˙3.Gma.Pac
104947˙2.Gog.Gre
152050˙3.Gma.Pac
152027˙1.Gch.Pac
104931˙1.Gmo.Gre
GeneScaffold˙2759
152921˙3.Gmo.Nor
200079˙1.Gmo.Can
118507˙7.Gmo.Ice
140233˙2.Gmo.Bal
115574˙2.Gmo.Far
140176˙1.Gmo.Cel
104725˙1.Bsa.Gre
114718˙1.Gmo.Far
152924˙3.Gmo.Nor
125968˙3.Gmo.Ice
114718˙4.Gmo.Far
115574˙1.Gmo.Far
117757˙1.Gmo.Ice
152924˙2.Gmo.Nor
200093˙5.Gmo.Can
200079˙3.Gmo.Can
117795˙1.Gmo.Ice
118214˙2.Gmo.Ice
140219˙3.Gmo.Bal
200093˙3.Gmo.Can
230
240
250
260
270
280
290
300
310
320
330

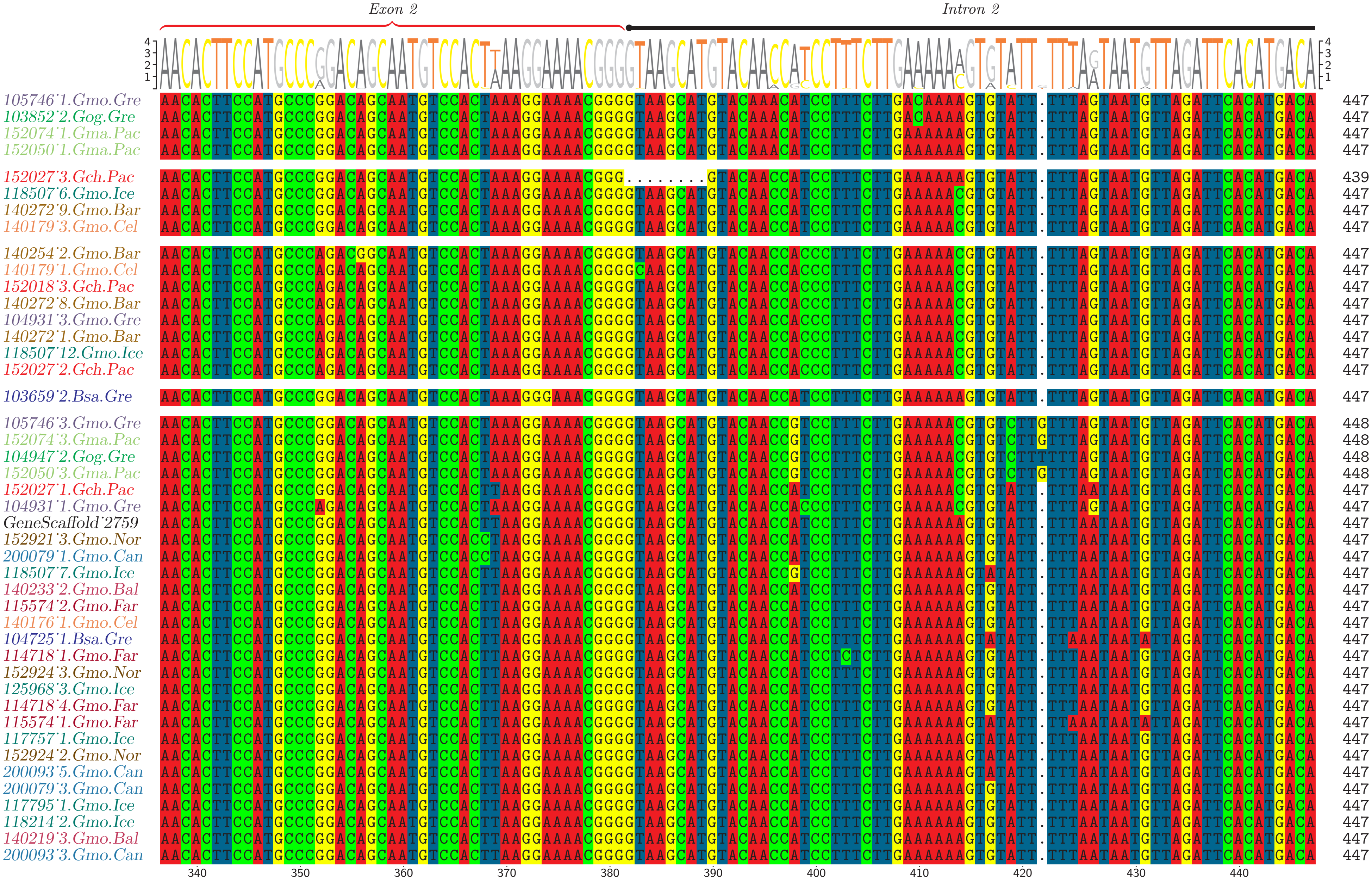
Exon 2
Intron 2
105746˙1.Gmo.Gre
103852˙2.Gog.Gre
152074˙1.Gma.Pac
152050˙1.Gma.Pac
152027˙3.Gch.Pac
118507˙6.Gmo.Ice
140272˙9.Gmo.Bar
140179˙3.Gmo.Cel
140254˙2.Gmo.Bar
140179˙1.Gmo.Cel
152018˙3.Gch.Pac
140272˙8.Gmo.Bar
104931˙3.Gmo.Gre
140272˙1.Gmo.Bar
118507˙12.Gmo.Ice
152027˙2.Gch.Pac
103659˙2.Bsa.Gre
105746˙3.Gmo.Gre
152074˙3.Gma.Pac
104947˙2.Gog.Gre
152050˙3.Gma.Pac
152027˙1.Gch.Pac
104931˙1.Gmo.Gre
GeneScaffold˙2759
152921˙3.Gmo.Nor
200079˙1.Gmo.Can
118507˙7.Gmo.Ice
140233˙2.Gmo.Bal
115574˙2.Gmo.Far
140176˙1.Gmo.Cel
104725˙1.Bsa.Gre
114718˙1.Gmo.Far
152924˙3.Gmo.Nor
125968˙3.Gmo.Ice
114718˙4.Gmo.Far
115574˙1.Gmo.Far
117757˙1.Gmo.Ice
152924˙2.Gmo.Nor
200093˙5.Gmo.Can
200079˙3.Gmo.Can
117795˙1.Gmo.Ice
118214˙2.Gmo.Ice
140219˙3.Gmo.Bal
200093˙3.Gmo.Can
340
350
360
370
380
390
400
410
420
430
440

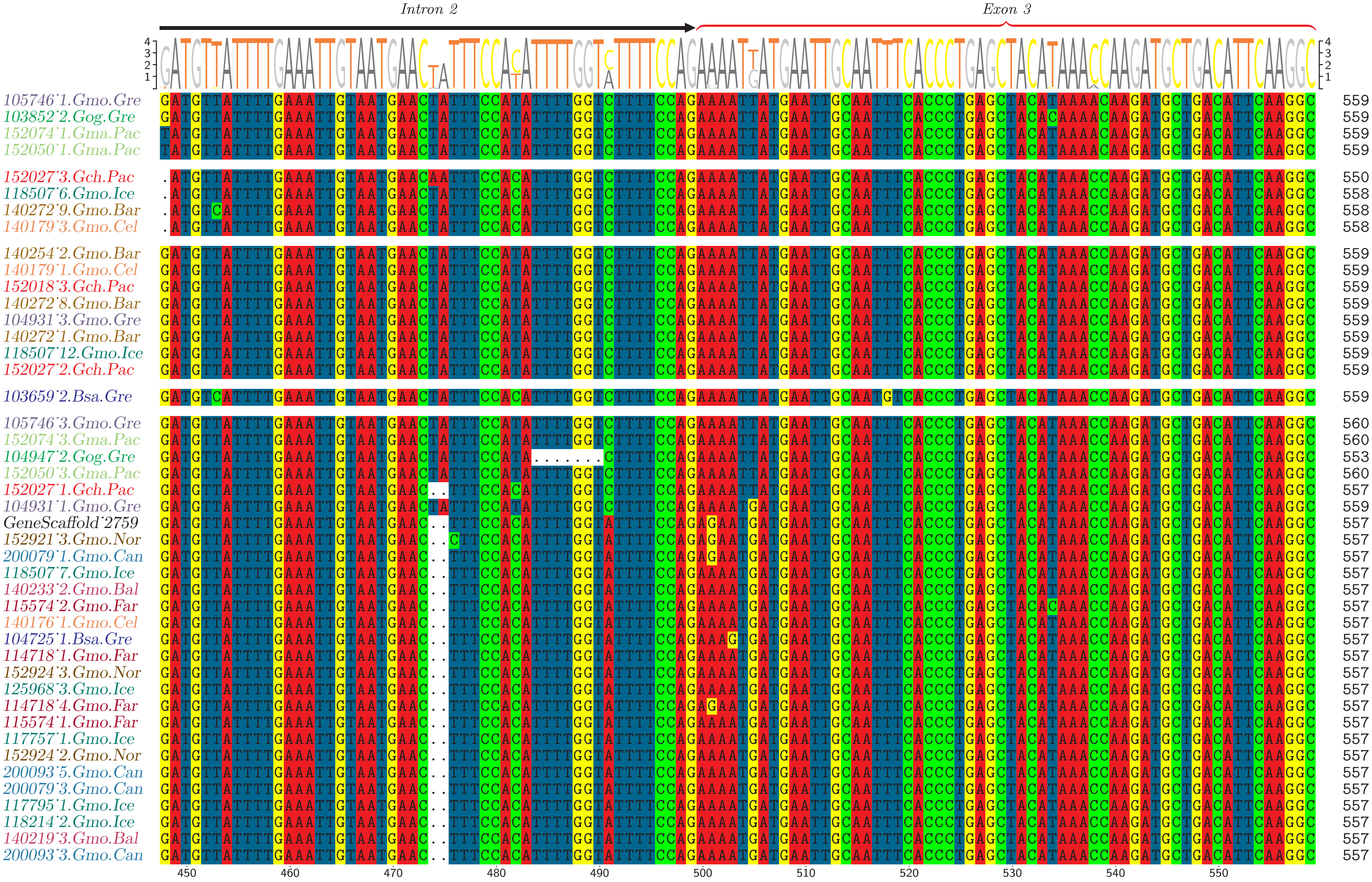

Intron 2
Exon 3
105746˙1.Gmo.Gre 559
103852˙2.Gog.Gre 559
152074˙1.Gma.Pac 559
152050˙1.Gma.Pac 559
152027˙3.Gch.Pac 550
118507˙6.Gmo.Ice 558
140272˙9.Gmo.Bar 558
140179˙3.Gmo.Cel 558
140254˙2.Gmo.Bar 559
140179˙1.Gmo.Cel 559
152018˙3.Gch.Pac 559
140272˙8.Gmo.Bar 559
104931˙3.Gmo.Gre 559
140272˙1.Gmo.Bar 559
118507˙12.Gmo.Ice 559
152027˙2.Gch.Pac 559
103659˙2.Bsa.Gre 559
105746˙3.Gmo.Gre 560
152074˙3.Gma.Pac 560
104947˙2.Gog.Gre 553
152050˙3.Gma.Pac 560
152027˙1.Gch.Pac 557
104931˙1.Gmo.Gre 559
GeneScaffold˙2759 557
152921˙3.Gmo.Nor 557
200079˙1.Gmo.Can 557
118507˙7.Gmo.Ice 557
140233˙2.Gmo.Bal 557
115574˙2.Gmo.Far 557
140176˙1.Gmo.Cel 557
104725˙1.Bsa.Gre 557
114718˙1.Gmo.Far 557
152924˙3.Gmo.Nor 557
125968˙3.Gmo.Ice 557
114718˙4.Gmo.Far 557
115574˙1.Gmo.Far 557
117757˙1.Gmo.Ice 557
152924˙2.Gmo.Nor 557
200093˙5.Gmo.Can 557
200079˙3.Gmo.Can 557
117795˙1.Gmo.Ice 557
118214˙2.Gmo.Ice 557
140219˙3.Gmo.Bal 557
200093˙3.Gmo.Can 557
450 460 470 480 490 500 510 520 530 540 550

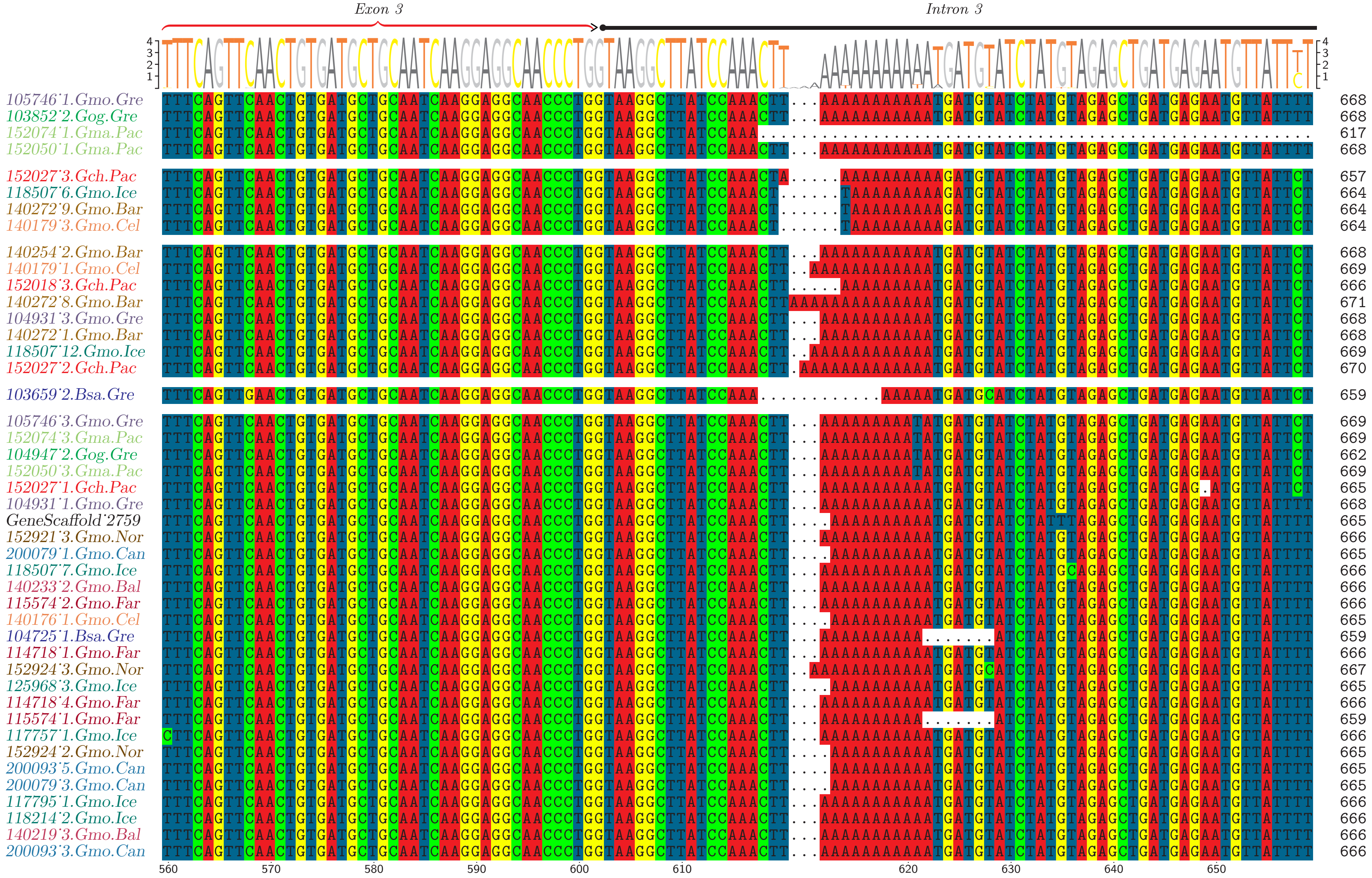

Exon 3
Intron 3
105746˙1.Gmo.Gre 668
103852˙2.Gog.Gre 668
152074˙1.Gma.Pac 617
152050˙1.Gma.Pac 668
152027˙3.Gch.Pac 657
118507˙6.Gmo.Ice 664
140272˙9.Gmo.Bar 664
140179˙3.Gmo.Cel 664
140254˙2.Gmo.Bar 668
140179˙1.Gmo.Cel 669
152018˙3.Gch.Pac 666
140272˙8.Gmo.Bar 671
104931˙3.Gmo.Gre 668
140272˙1.Gmo.Bar 668
118507˙12.Gmo.Ice 669
152027˙2.Gch.Pac 670
103659˙2.Bsa.Gre 659
105746˙3.Gmo.Gre 669
152074˙3.Gma.Pac 669
104947˙2.Gog.Gre 662
152050˙3.Gma.Pac 669
152027˙1.Gch.Pac 665
104931˙1.Gmo.Gre 668
GeneScaffold˙2759 665
152921˙3.Gmo.Nor 666
200079˙1.Gmo.Can 665
118507˙7.Gmo.Ice 666
140233˙2.Gmo.Bal 666
115574˙2.Gmo.Far 666
140176˙1.Gmo.Cel 665
104725˙1.Bsa.Gre 659
114718˙1.Gmo.Far 666
152924˙3.Gmo.Nor 667
125968˙3.Gmo.Ice 665
114718˙4.Gmo.Far 666
115574˙1.Gmo.Far 659
117757˙1.Gmo.Ice 666
152924˙2.Gmo.Nor 665
200093˙5.Gmo.Can 665
200079˙3.Gmo.Can 665
117795˙1.Gmo.Ice 666
118214˙2.Gmo.Ice 666
140219˙3.Gmo.Bal 666
200093˙3.Gmo.Can 666
560 570 580 590 600 610 620 630 640 650

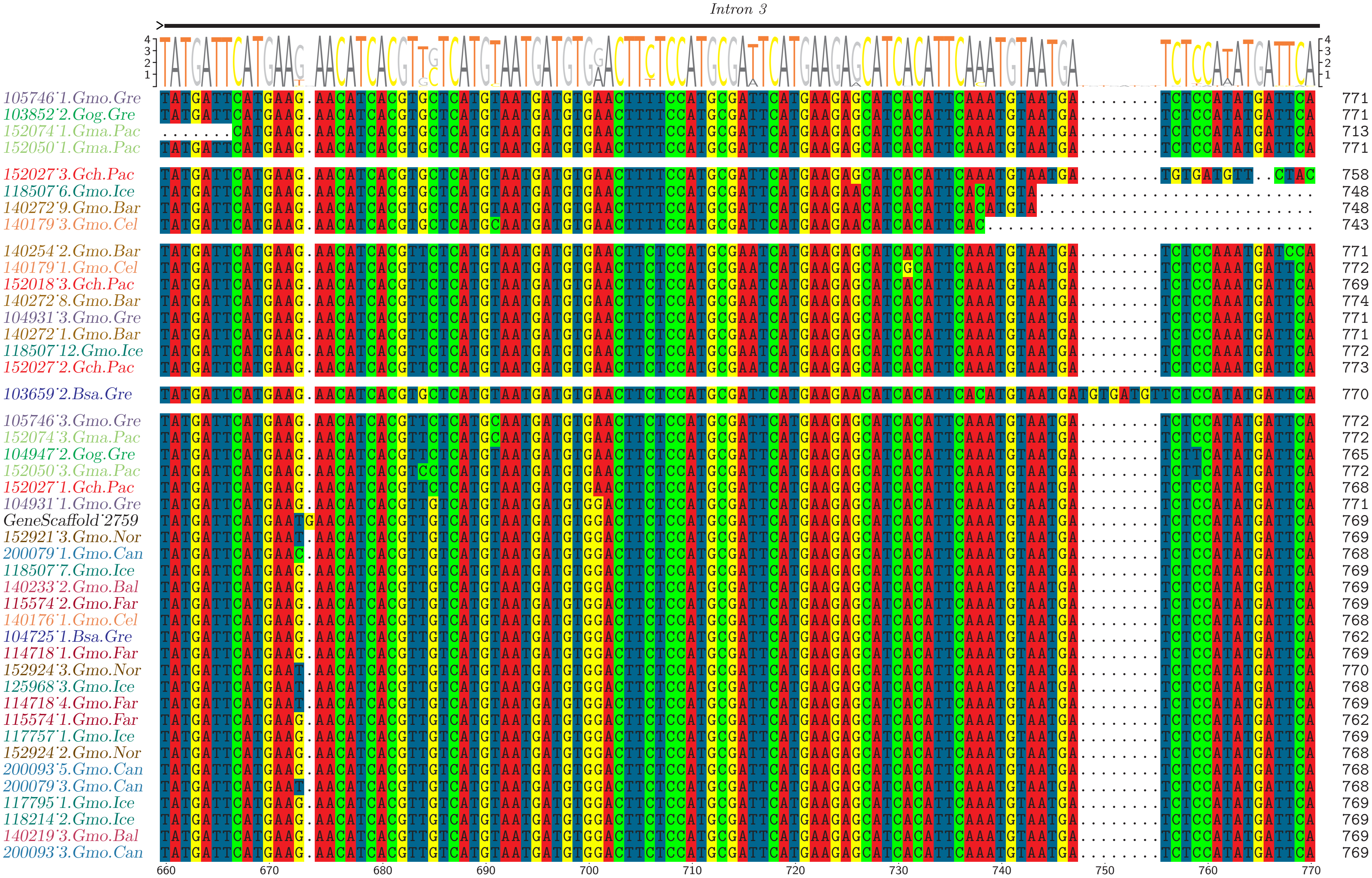
Intron 3
105746˙1.Gmo.Gre 771
103852˙2.Gog.Gre 771
152074˙1.Gma.Pac 713
152050˙1.Gma.Pac 771
152027˙3.Gch.Pac 758
118507˙6.Gmo.Ice 748
140272˙9.Gmo.Bar 748
140179˙3.Gmo.Cel 743
140254˙2.Gmo.Bar 771
140179˙1.Gmo.Cel 772
152018˙3.Gch.Pac 769
140272˙8.Gmo.Bar 774
104931˙3.Gmo.Gre 771
140272˙1.Gmo.Bar 771
118507˙12.Gmo.Ice 772
152027˙2.Gch.Pac 773
103659˙2.Bsa.Gre 770
105746˙3.Gmo.Gre 772
152074˙3.Gma.Pac 772
104947˙2.Gog.Gre 765
152050˙3.Gma.Pac 772
152027˙1.Gch.Pac 768
104931˙1.Gmo.Gre 771
GeneScaffold˙2759 769
152921˙3.Gmo.Nor 769
200079˙1.Gmo.Can 768
118507˙7.Gmo.Ice 769
140233˙2.Gmo.Bal 769
115574˙2.Gmo.Far 769
140176˙1.Gmo.Cel 768
104725˙1.Bsa.Gre 762
114718˙1.Gmo.Far 769
152924˙3.Gmo.Nor 770
125968˙3.Gmo.Ice 768
114718˙4.Gmo.Far 769
115574˙1.Gmo.Far 762
117757˙1.Gmo.Ice 769
152924˙2.Gmo.Nor 768
200093˙5.Gmo.Can 768
200079˙3.Gmo.Can 768
117795˙1.Gmo.Ice 769
118214˙2.Gmo.Ice 769
140219˙3.Gmo.Bal 769
200093˙3.Gmo.Can 769
660 670 680 690 700 710 720 730 740 750 760 770

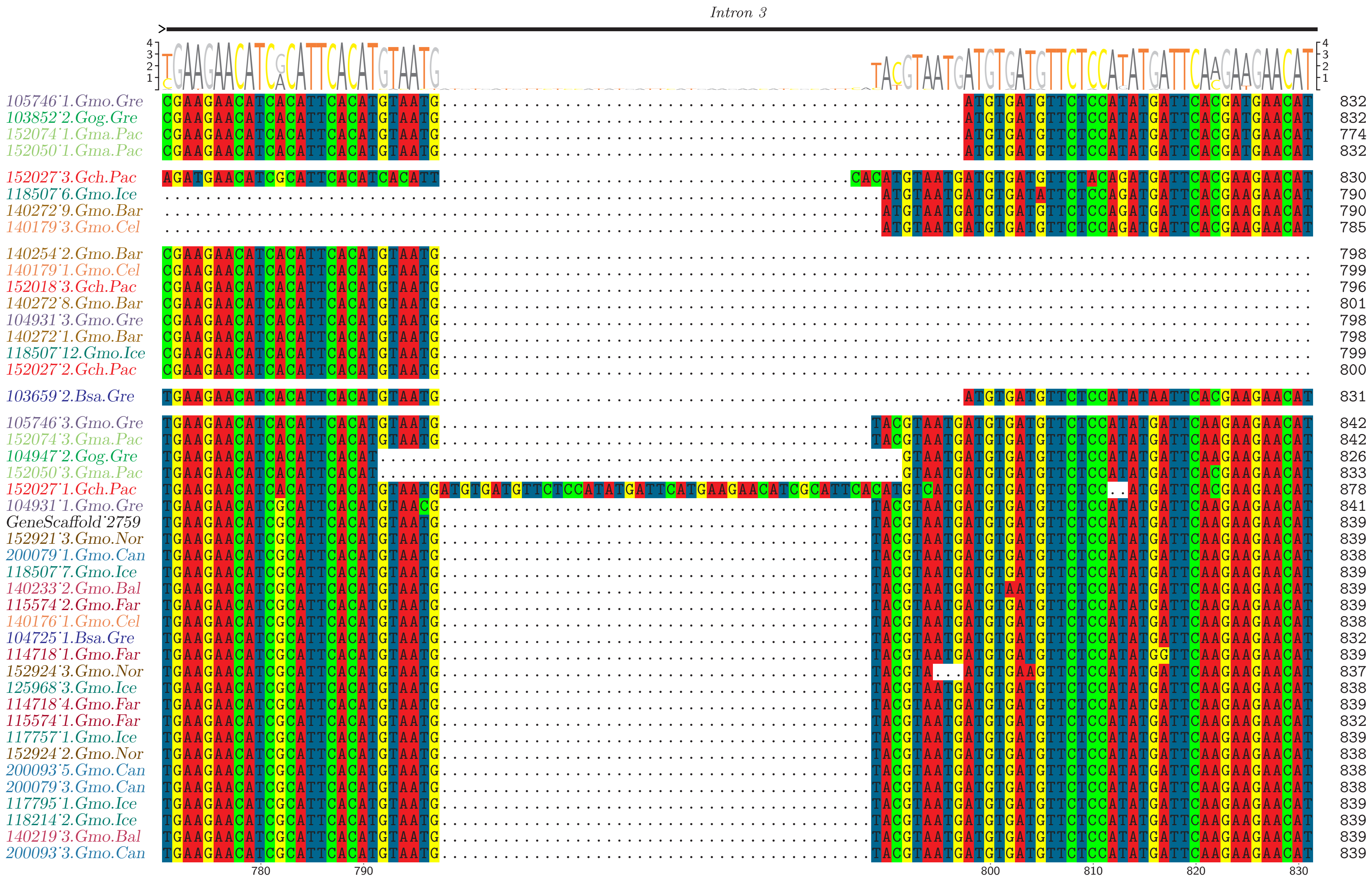
Intron 3
105746˙1.Gmo.Gre 832
103852˙2.Gog.Gre 832
152074˙1.Gma.Pac 774
152050˙1.Gma.Pac 832
152027˙3.Gch.Pac 830
118507˙6.Gmo.Ice 790
140272˙9.Gmo.Bar 790
140179˙3.Gmo.Cel 785
140254˙2.Gmo.Bar 798
140179˙1.Gmo.Cel 799
152018˙3.Gch.Pac 796
140272˙8.Gmo.Bar 801
104931˙3.Gmo.Gre 798
140272˙1.Gmo.Bar 798
118507˙12.Gmo.Ice 799
152027˙2.Gch.Pac 800
103659˙2.Bsa.Gre 831
105746˙3.Gmo.Gre 842
152074˙3.Gma.Pac 842
104947˙2.Gog.Gre 826
152050˙3.Gma.Pac 833
152027˙1.Gch.Pac 878
104931˙1.Gmo.Gre 841
GeneScaffold˙2759 839
152921˙3.Gmo.Nor 839
200079˙1.Gmo.Can 838
118507˙7.Gmo.Ice 839
140233˙2.Gmo.Bal 839
115574˙2.Gmo.Far 839
140176˙1.Gmo.Cel 838
104725˙1.Bsa.Gre 832
114718˙1.Gmo.Far 839
152924˙3.Gmo.Nor 837
125968˙3.Gmo.Ice 838
114718˙4.Gmo.Far 839
115574˙1.Gmo.Far 832
117757˙1.Gmo.Ice 839
152924˙2.Gmo.Nor 838
200093˙5.Gmo.Can 838
200079˙3.Gmo.Can 838
117795˙1.Gmo.Ice 839
118214˙2.Gmo.Ice 839
140219˙3.Gmo.Bal 839
200093˙3.Gmo.Can 839
780
790
800
810
820
830

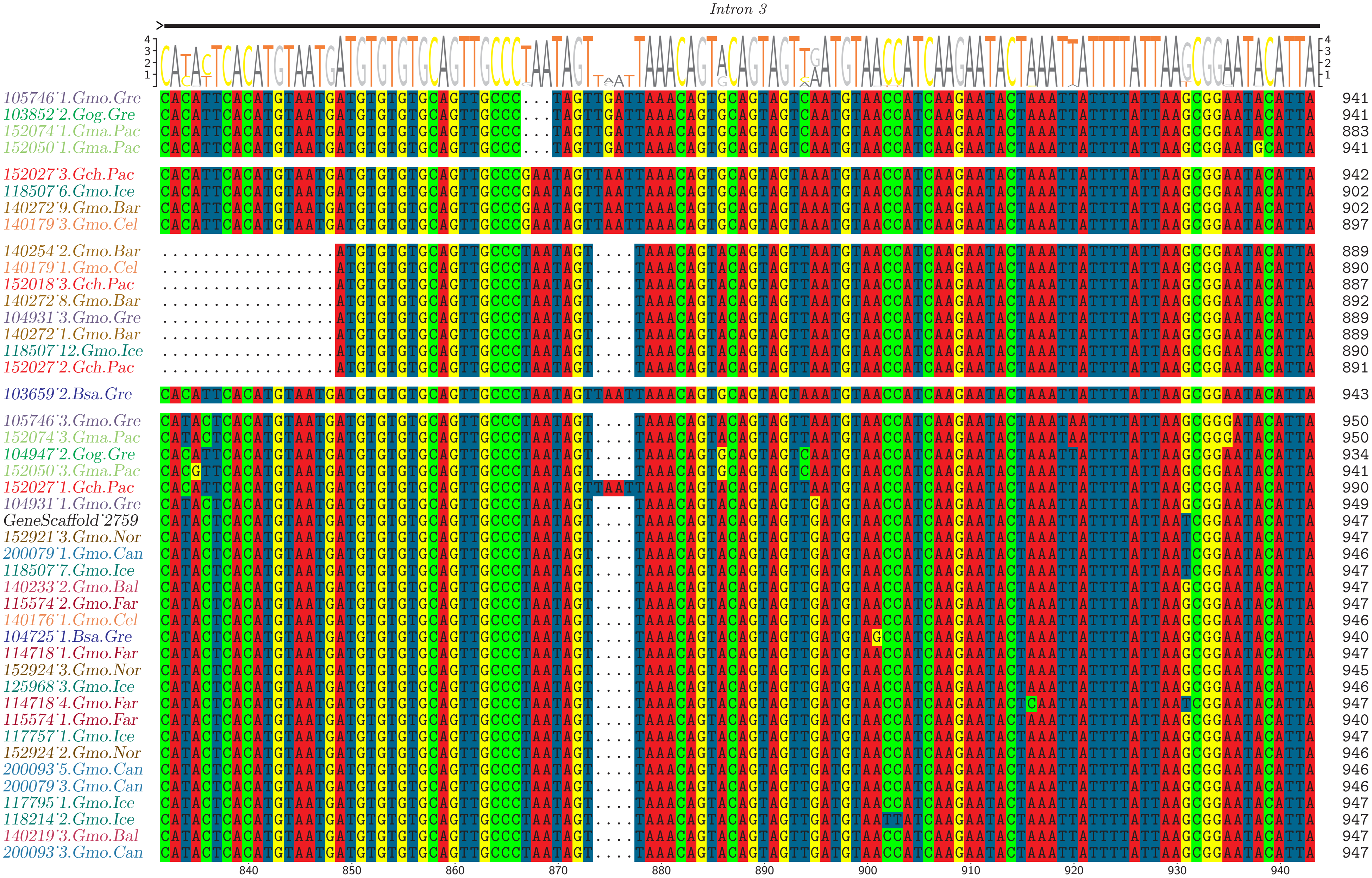
Intron 3
105746˙1.Gmo.Gre 941
103852˙2.Gog.Gre 941
152074˙1.Gma.Pac 883
152050˙1.Gma.Pac 941
152027˙3.Gch.Pac 942
118507˙6.Gmo.Ice 902
140272˙9.Gmo.Bar 902
140179˙3.Gmo.Cel 897
140254˙2.Gmo.Bar 889
140179˙1.Gmo.Cel 890
152018˙3.Gch.Pac 887
140272˙8.Gmo.Bar 892
104931˙3.Gmo.Gre 889
140272˙1.Gmo.Bar 889
118507˙12.Gmo.Ice 890
152027˙2.Gch.Pac 891
103659˙2.Bsa.Gre 943
105746˙3.Gmo.Gre 950
152074˙3.Gma.Pac 950
104947˙2.Gog.Gre 934
152050˙3.Gma.Pac 941
152027˙1.Gch.Pac 990
104931˙1.Gmo.Gre 949
GeneScaffold˙2759 947
152921˙3.Gmo.Nor 947
200079˙1.Gmo.Can 946
118507˙7.Gmo.Ice 947
140233˙2.Gmo.Bal 947
115574˙2.Gmo.Far 947
140176˙1.Gmo.Cel 946
104725˙1.Bsa.Gre 940
114718˙1.Gmo.Far 947
152924˙3.Gmo.Nor 945
125968˙3.Gmo.Ice 946
114718˙4.Gmo.Far 947
115574˙1.Gmo.Far 940
117757˙1.Gmo.Ice 947
152924˙2.Gmo.Nor 946
200093˙5.Gmo.Can 946
200079˙3.Gmo.Can 946
117795˙1.Gmo.Ice 947
118214˙2.Gmo.Ice 947
140219˙3.Gmo.Bal 947
200093˙3.Gmo.Can 947
840 850 860 870 880 890 900 910 920 930 940

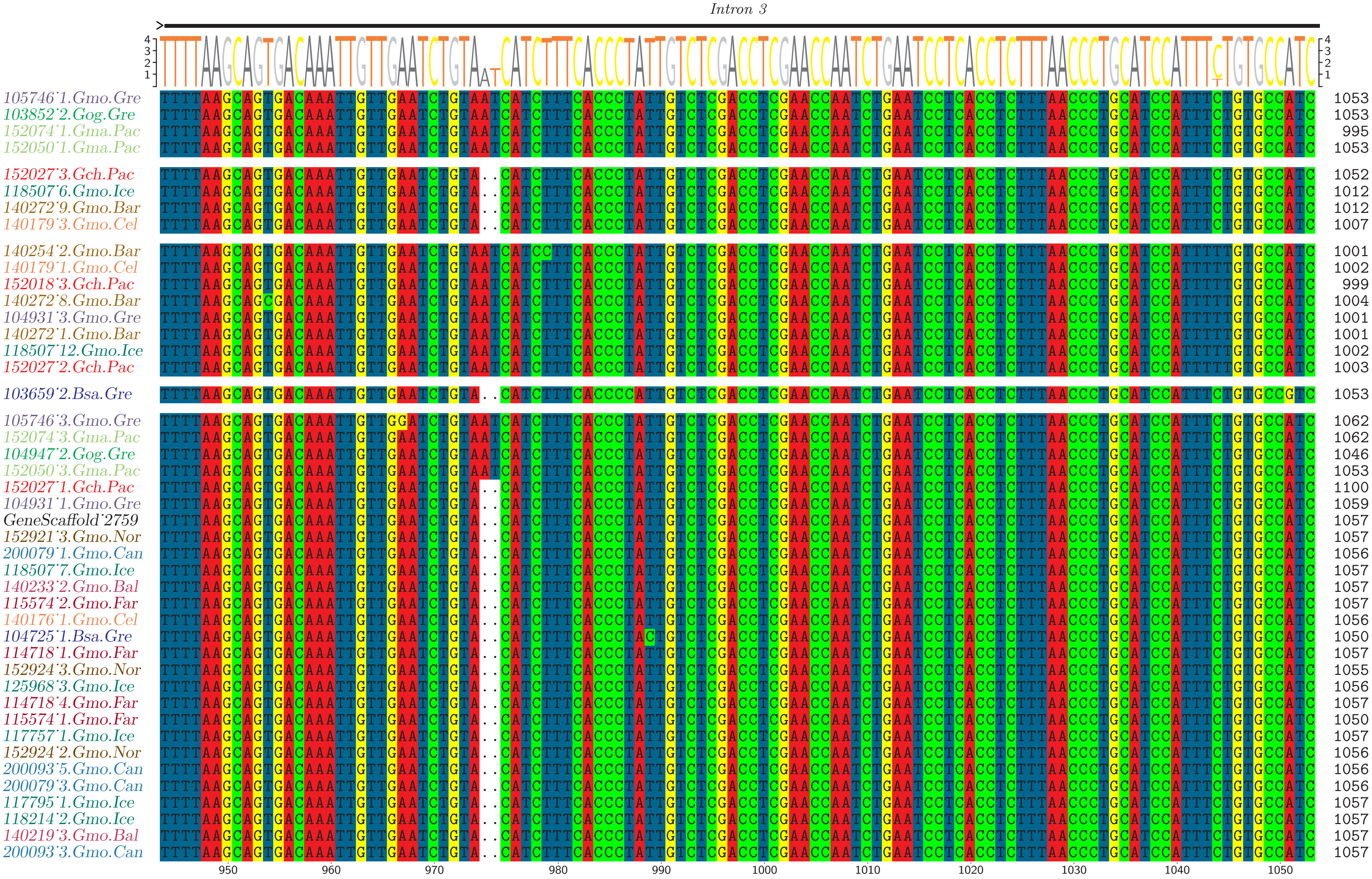

Intron 3
105746˙1.Gmo.Gre 1053
103852˙2.Gog.Gre 1053
152074˙1.Gma.Pac 995
152050˙1.Gma.Pac 1053
152027˙3.Gch.Pac 1052
118507˙6.Gmo.Ice 1012
140272˙9.Gmo.Bar 1012
140179˙3.Gmo.Cel 1007
140254˙2.Gmo.Bar 1001
140179˙1.Gmo.Cel 1002
152018˙3.Gch.Pac 999
140272˙8.Gmo.Bar 1004
104931˙3.Gmo.Gre 1001
140272˙1.Gmo.Bar 1001
118507˙12.Gmo.Ice 1002
152027˙2.Gch.Pac 1003
103659˙2.Bsa.Gre 1053
105746˙3.Gmo.Gre 1062
152074˙3.Gma.Pac 1062
104947˙2.Gog.Gre 1046
152050˙3.Gma.Pac 1053
152027˙1.Gch.Pac 1100
104931˙1.Gmo.Gre 1059
GeneScaffold˙2759 1057
152921˙3.Gmo.Nor 1057
200079˙1.Gmo.Can 1056
118507˙7.Gmo.Ice 1057
140233˙2.Gmo.Bal 1057
115574˙2.Gmo.Far 1057
140176˙1.Gmo.Cel 1056
104725˙1.Bsa.Gre 1050
114718˙1.Gmo.Far 1057
152924˙3.Gmo.Nor 1055
125968˙3.Gmo.Ice 1056
114718˙4.Gmo.Far 1057
115574˙1.Gmo.Far 1050
117757˙1.Gmo.Ice 1057
152924˙2.Gmo.Nor 1056
200093˙5.Gmo.Can 1056
200079˙3.Gmo.Can 1056
117795˙1.Gmo.Ice 1057
118214˙2.Gmo.Ice 1057
140219˙3.Gmo.Bal 1057
200093˙3.Gmo.Can 1057
950 960 970 980 990 1000 1010 1020 1030 1040 1050

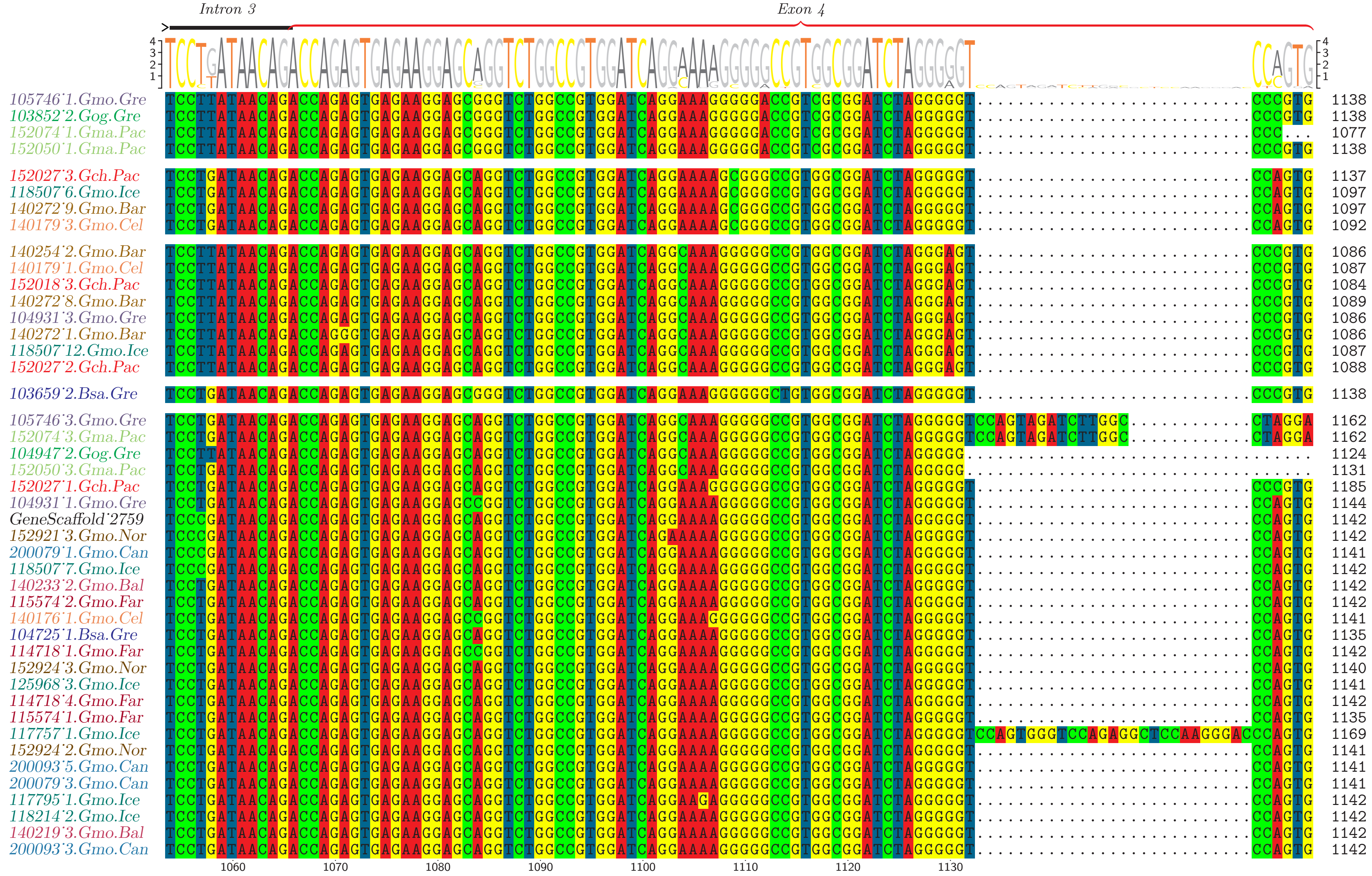

Intron 3
Exon 4
105746˙1.Gmo.Gre 1138
103852˙2.Gog.Gre 1138
152074˙1.Gma.Pac 1077
152050˙1.Gma.Pac 1138
152027˙3.Gch.Pac 1137
118507˙6.Gmo.Ice 1097
140272˙9.Gmo.Bar 1097
140179˙3.Gmo.Cel 1092
140254˙2.Gmo.Bar 1086
140179˙1.Gmo.Cel 1087
152018˙3.Gch.Pac 1084
140272˙8.Gmo.Bar 1089
104931˙3.Gmo.Gre 1086
140272˙1.Gmo.Bar 1086
118507˙12.Gmo.Ice 1087
152027˙2.Gch.Pac 1088
103659˙2.Bsa.Gre 1138
105746˙3.Gmo.Gre 1162
152074˙3.Gma.Pac 1162
104947˙2.Gog.Gre 1124
152050˙3.Gma.Pac 1131
152027˙1.Gch.Pac 1185
104931˙1.Gmo.Gre 1144
GeneScaffold˙2759 1142
152921˙3.Gmo.Nor 1142
200079˙1.Gmo.Can 1141
118507˙7.Gmo.Ice 1142
140233˙2.Gmo.Bal 1142
115574˙2.Gmo.Far 1142
140176˙1.Gmo.Cel 1141
104725˙1.Bsa.Gre 1135
114718˙1.Gmo.Far 1142
152924˙3.Gmo.Nor 1140
125968˙3.Gmo.Ice 1141
114718˙4.Gmo.Far 1142
115574˙1.Gmo.Far 1135
117757˙1.Gmo.Ice 1169
152924˙2.Gmo.Nor 1141
200093˙5.Gmo.Can 1141
200079˙3.Gmo.Can 1141
117795˙1.Gmo.Ice 1142
118214˙2.Gmo.Ice 1142
140219˙3.Gmo.Bal 1142
200093˙3.Gmo.Can 1142
1060
1070
1080
1090
1100
1110
1120
1130

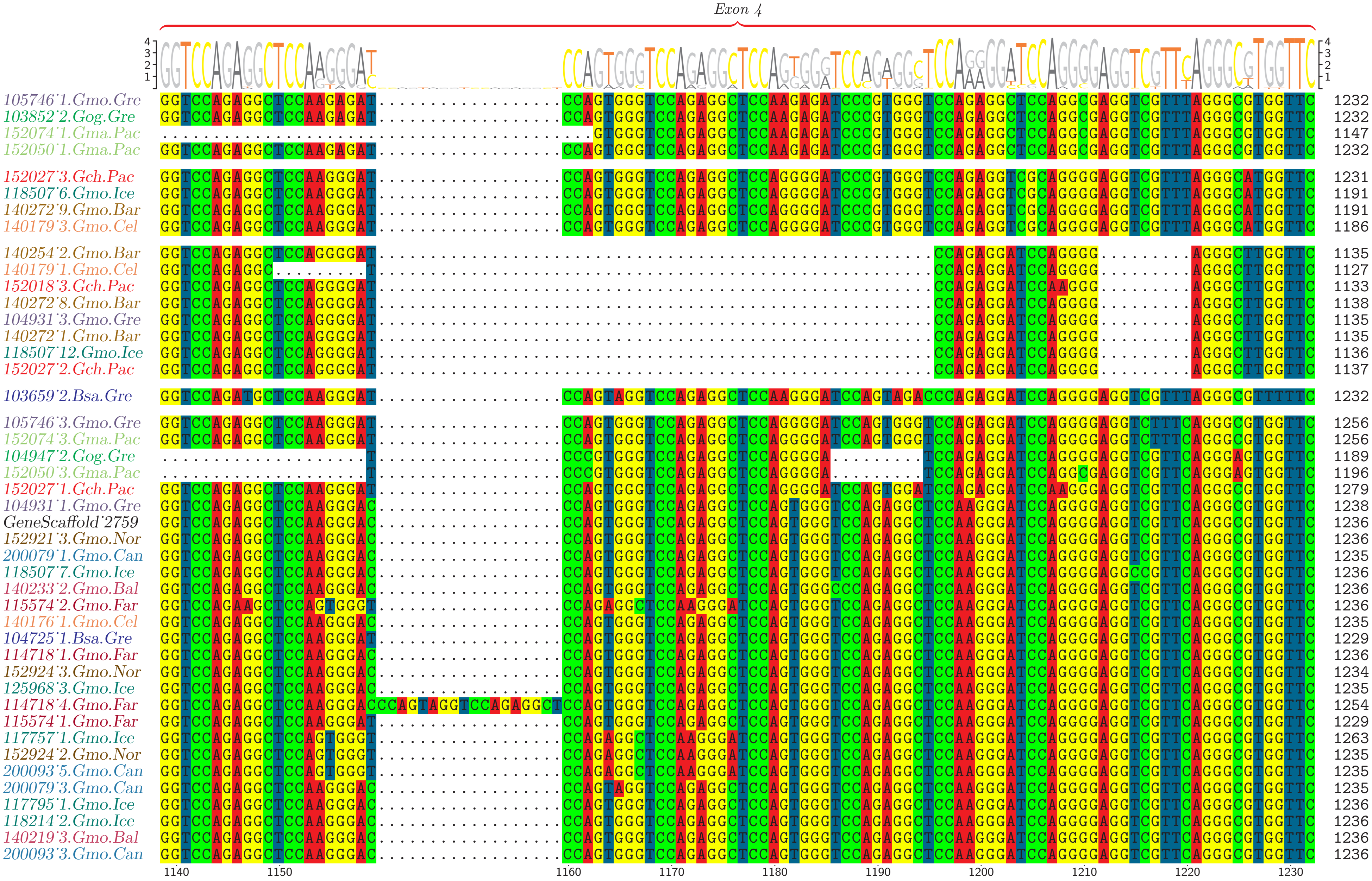
Exon 4
105746˙1.Gmo.Gre 1232
103852˙2.Gog.Gre 1232
152074˙1.Gma.Pac 1147
152050˙1.Gma.Pac 1232
152027˙3.Gch.Pac 1231
118507˙6.Gmo.Ice 1191
140272˙9.Gmo.Bar 1191
140179˙3.Gmo.Cel 1186
140254˙2.Gmo.Bar 1135
140179˙1.Gmo.Cel 1127
152018˙3.Gch.Pac 1133
140272˙8.Gmo.Bar 1138
104931˙3.Gmo.Gre 1135
140272˙1.Gmo.Bar 1135
118507˙12.Gmo.Ice 1136
152027˙2.Gch.Pac 1137
103659˙2.Bsa.Gre 1232
105746˙3.Gmo.Gre 1256
152074˙3.Gma.Pac 1256
104947˙2.Gog.Gre 1189
152050˙3.Gma.Pac 1196
152027˙1.Gch.Pac 1279
104931˙1.Gmo.Gre 1238
GeneScaffold˙2759 1236
152921˙3.Gmo.Nor 1236
200079˙1.Gmo.Can 1235
118507˙7.Gmo.Ice 1236
140233˙2.Gmo.Bal 1236
115574˙2.Gmo.Far 1236
140176˙1.Gmo.Cel 1235
104725˙1.Bsa.Gre 1229
114718˙1.Gmo.Far 1236
152924˙3.Gmo.Nor 1234
125968˙3.Gmo.Ice 1235
114718˙4.Gmo.Far 1254
115574˙1.Gmo.Far 1229
117757˙1.Gmo.Ice 1263
152924˙2.Gmo.Nor 1235
200093˙5.Gmo.Can 1235
200079˙3.Gmo.Can 1235
117795˙1.Gmo.Ice 1236
118214˙2.Gmo.Ice 1236
140219˙3.Gmo.Bal 1236
200093˙3.Gmo.Can 1236
1140 1150 1160 1170 1180 1190 1200 1210 1220 1230

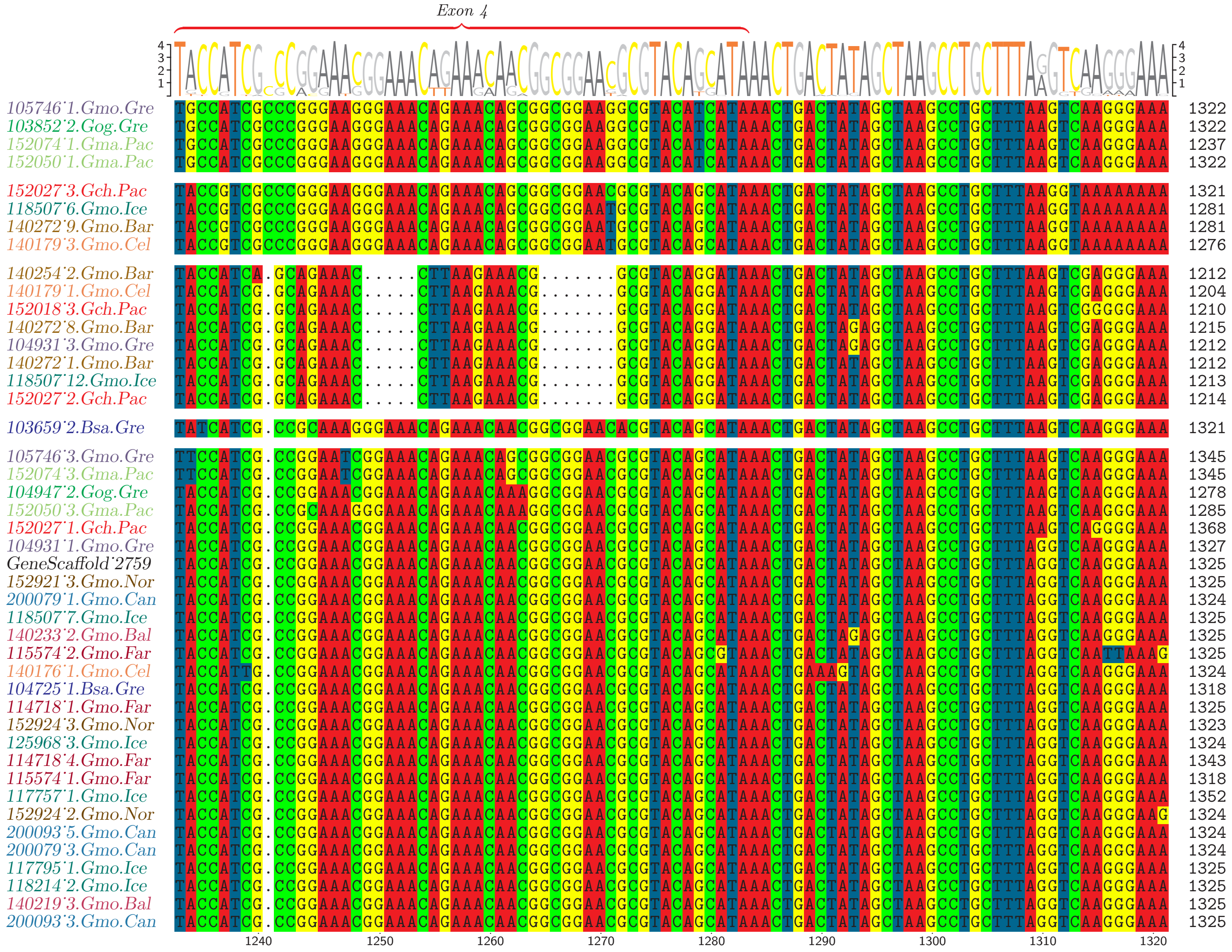

Figure S2. Alignment of nucleotide sequences of cathelicidin among clones from various individuals of Atlantic cod and four closely related taxa. Bsa.Gre (*Boreogadus saida*), Gch.Pac (*Gadus chalcogrammus*), Gma.Pac (*Gadus macrocephalus*), Gog.Gre (*Gadus ogac*) and Gmo (*Gadus morhua*) from various locations; Iceland (Gmo.Ice), Greenland (Gmo.Gre), Barents Sea (Gmo.Bar), Celtic Sea (Gmo.Cel), Baltic Sea (Gmo.Bal), Norway (Gmo.Nor), Faroe Islands (Gmo.Far), Canada (Gmo.Can).

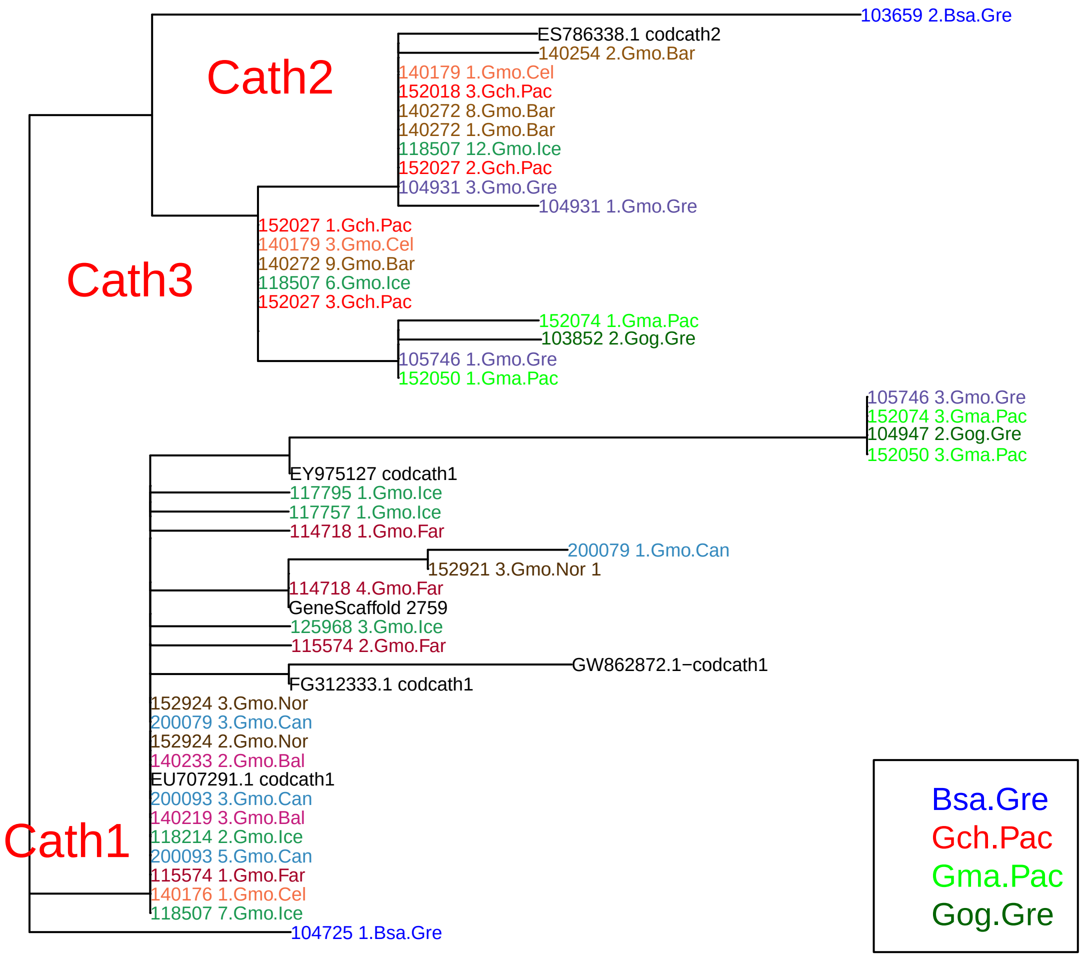

Figure S3. Maximum likelihood phylogenetic tree of Cathelicidin amino acid sequences of the conserved part. Phylogenetic tree built on amino acid sequences in exons 1, 2, and 3 combined, the conserved part of cathelicidin, of clones from various individuals of Atlantic cod and four closely related taxa. Bsa.Gre (*Boreogadus saida*), Gch.Pac (*Gadus chalcogrammus*), Gma.Pac (*Gadus macrocephalus*), Gog.Gre (*Gadus ogac*) and Gmo (*Gadus morhua*) from various locations; Iceland (Gmo.Ice), Greenland (Gmo.Gre), Barents Sea (Gmo.Bar), Celtic Sea (Gmo.Cel), Baltic Sea (Gmo.Bal), Norway (Gmo.Nor), Faroe Islands (Gmo.Far), Canada (Gmo.Can).

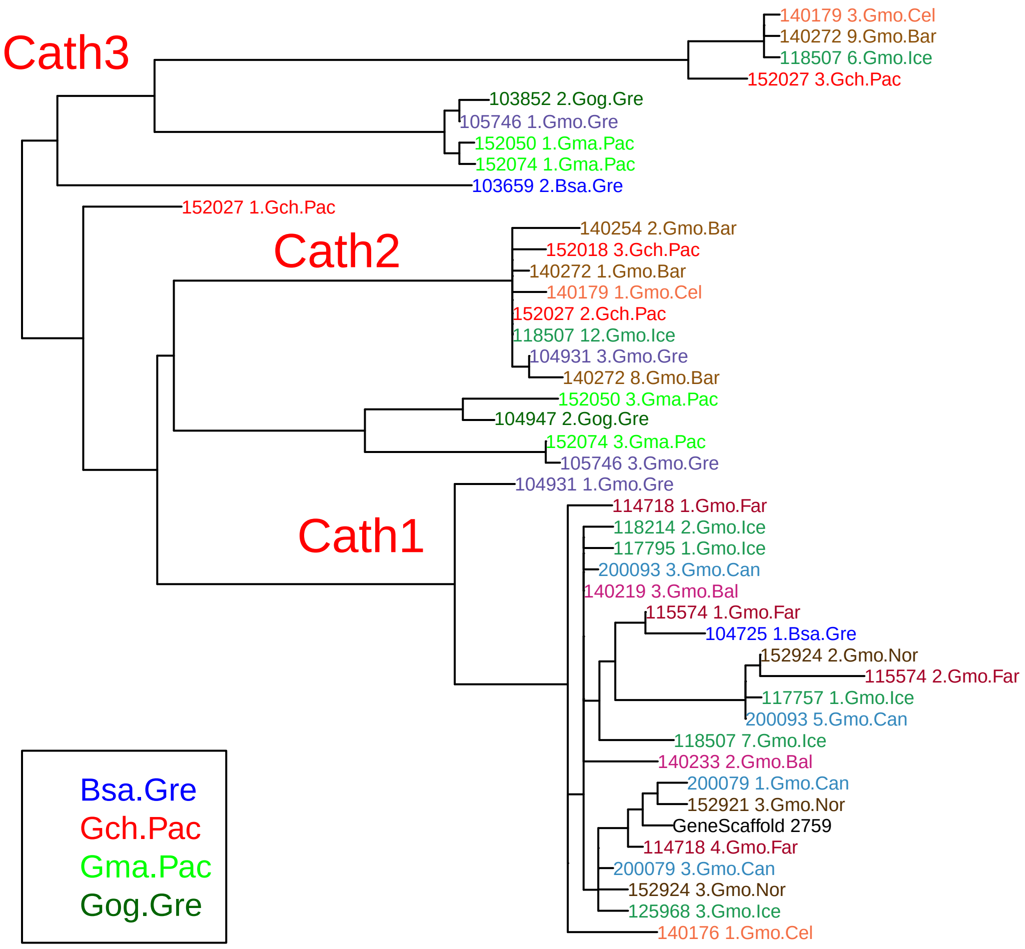

Figure S4. Maximum likelihood phylogenetic tree of nucleotide sequences. Phylogenetic tree of nucleotide sequences of the cathelicidin gene from 43 representatve clones of various individuals of Atlantic cod and four sister taxa. Bsa.Gre (*Boreogadus saida*), Gch.Pac (*Gadus chalcogrammus*), Gma.Pac (*Gadus macrocephalus*), Gog.Gre (*Gadus ogac*) and Gmo (*Gadus morhua*) from various locations; Iceland (Gmo.Ice), Greenland (Gmo.Gre), Barents Sea (Gmo.Bar), Celtic Sea (Gmo.Cel), Baltic Sea (Gmo.Bal), Norway (Gmo.Nor), Faroe Islands (Gmo.Far), Canada (Gmo.Can).

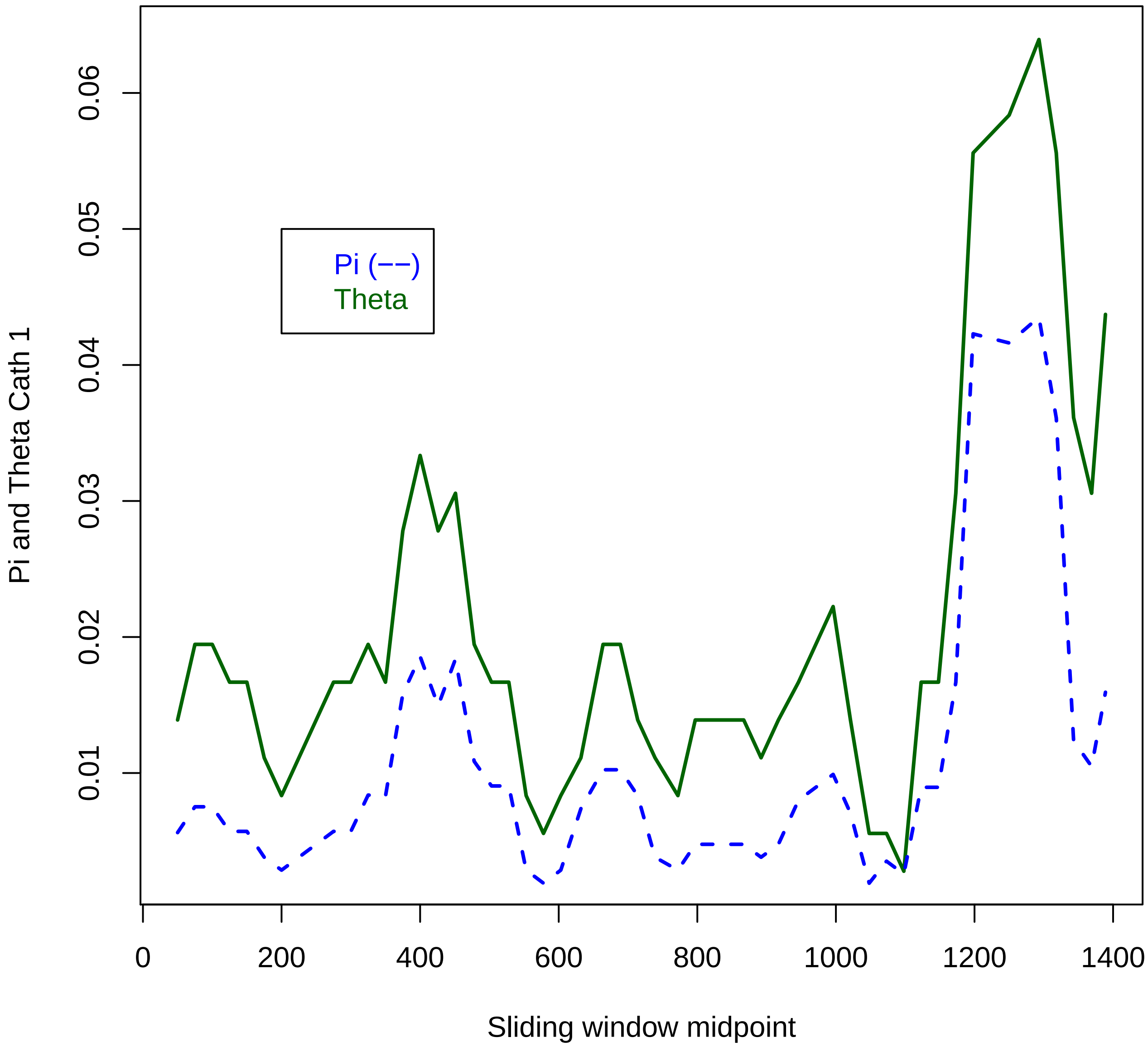

Figure S5. Sliding window analysis of nucleotide diversity π and the scaled mutation rate θ for Cath1. Window length was 100 bp with a 25 bp step size.

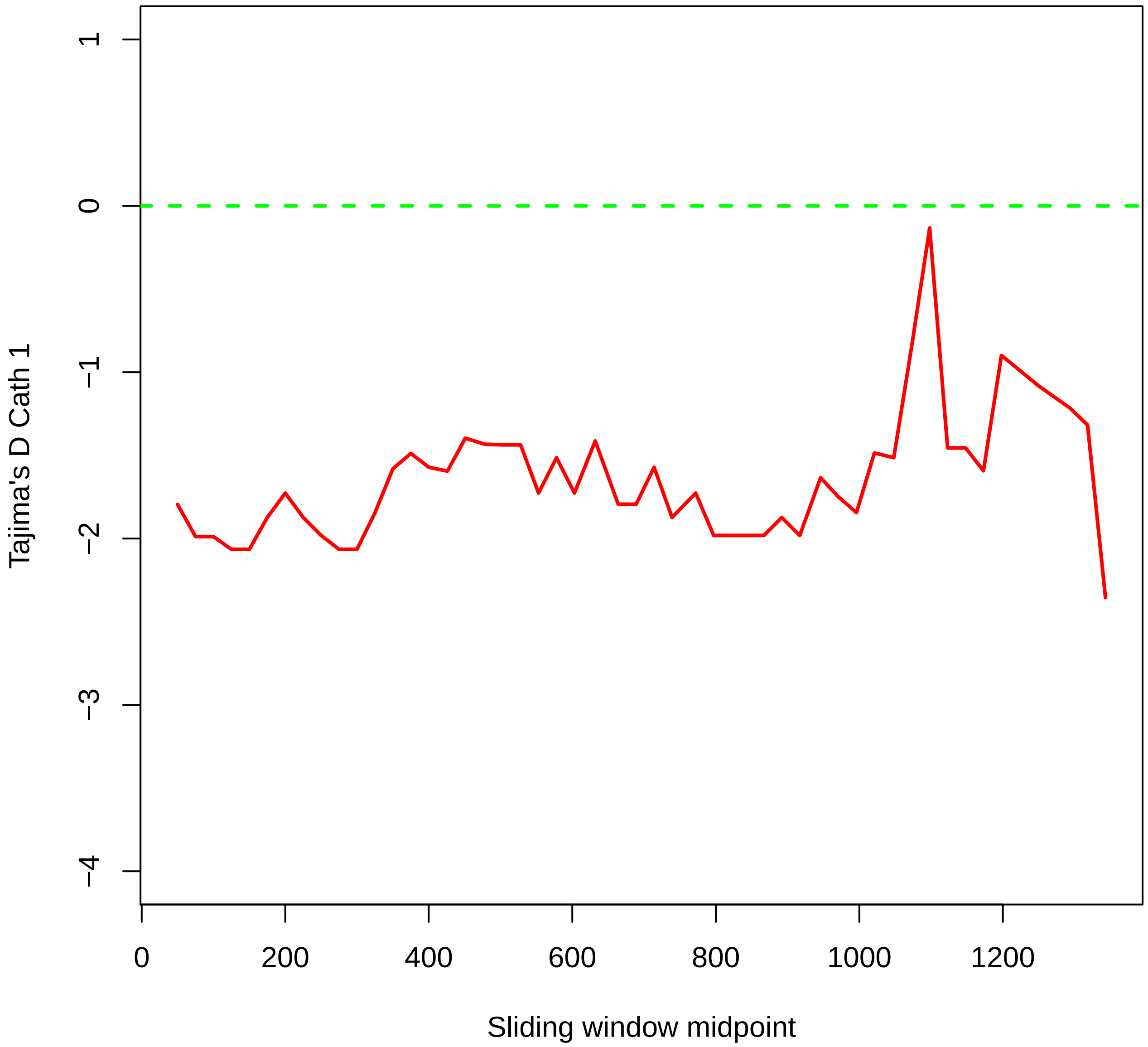

Figure S6. Sliding window Tajima's D for Cath1. Window length was 100 bp with a 25 bp step size.

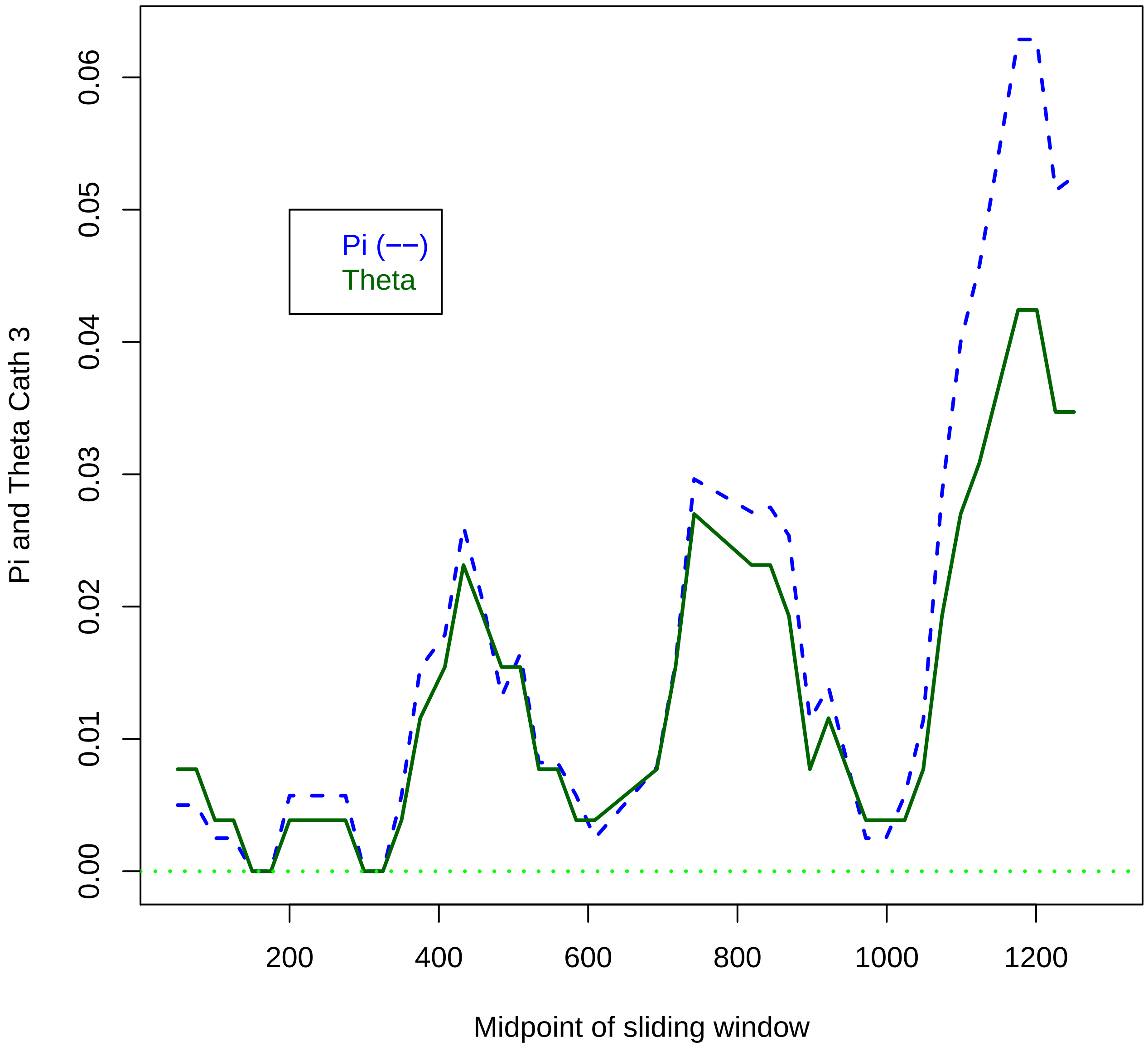

Figure S7. Sliding window analysis of nucleotide diversity π and the scaled mutation rate θ for Cath3. Window length was 100 bp with a 25 bp step size.

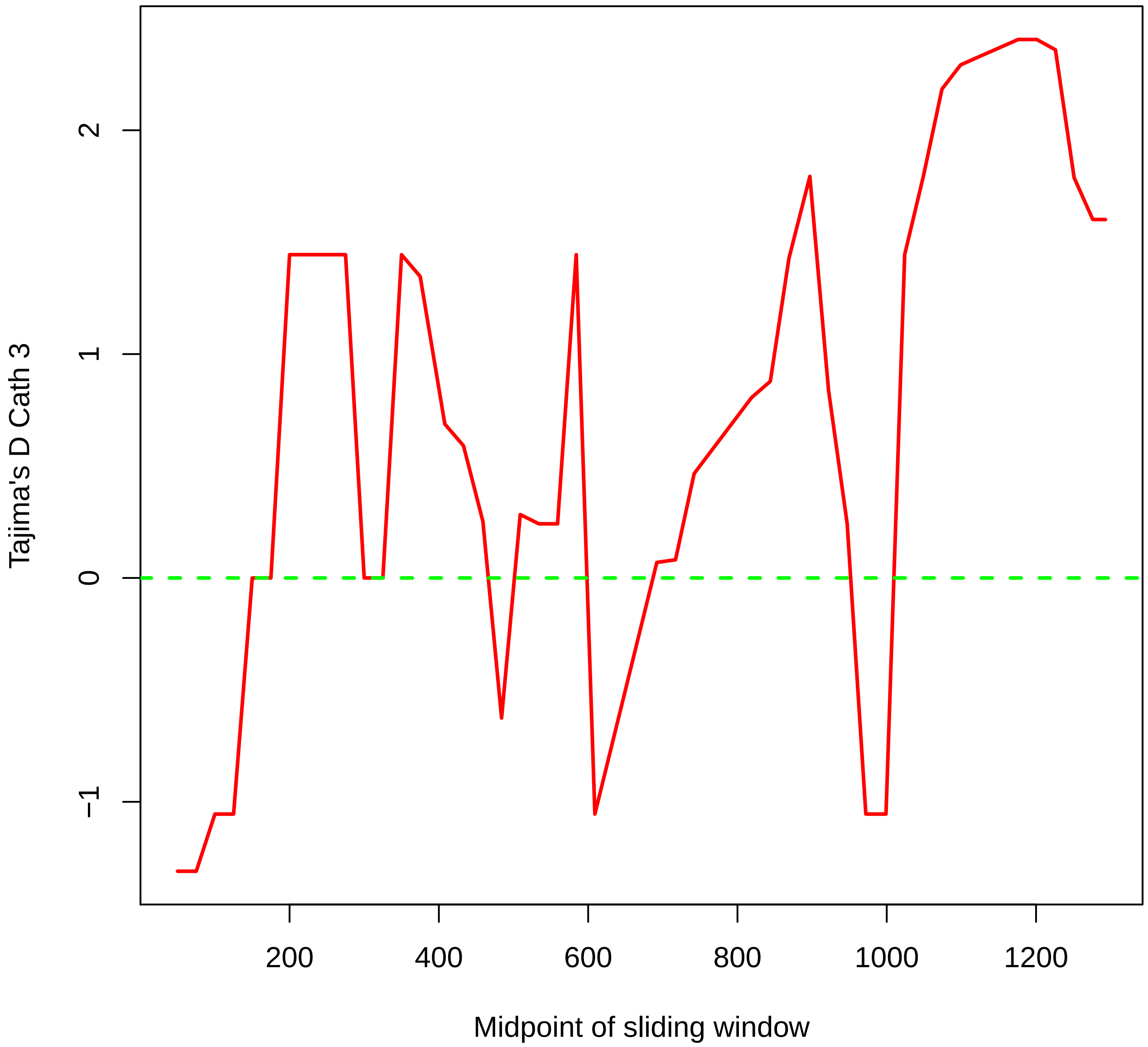

Figure S8. Sliding window Tajima's D for Cath3. Window length was 100 bp with a 25 bp step size.
