# Supplementary material for: Trans-species polymorphism at antimicrobial innate immunity cathelicidin genes of Atlantic cod and related species
Source: PeerJ. 2015 May 21;3:e976. doi: 10.7717/peerj.976 (PMC4451034; doi:10.7717/peerj.976)
Supplement: Figure S1 — Highly polymorphic sites are boxed. The four conserved cysteine residues characterizing cathelicidin are shaded. Up arrows represent positively selected sites and down arrows negatively selected sites from Tables 1 and 2. Bsa.Gre (Boreogadus saida), Gch.Pac (Gadus chalcogrammus), Gma.Pac (Gadus macrocephalus), Gog.Gre (Gadus ogac) and Gmo (Gadus morhua) from various locations; Iceland (Gmo.Ice), Greenland (Gmo.Gre), Barents Sea (Gmo.Bar), Celtic Sea (Gmo.Cel), Baltic Sea (Gmo.Bal), Norway (Gmo.Nor), Faeroe Islands (Gmo.Far), Canada (Gmo.Can). [file peerj-03-976-s002.pdf]

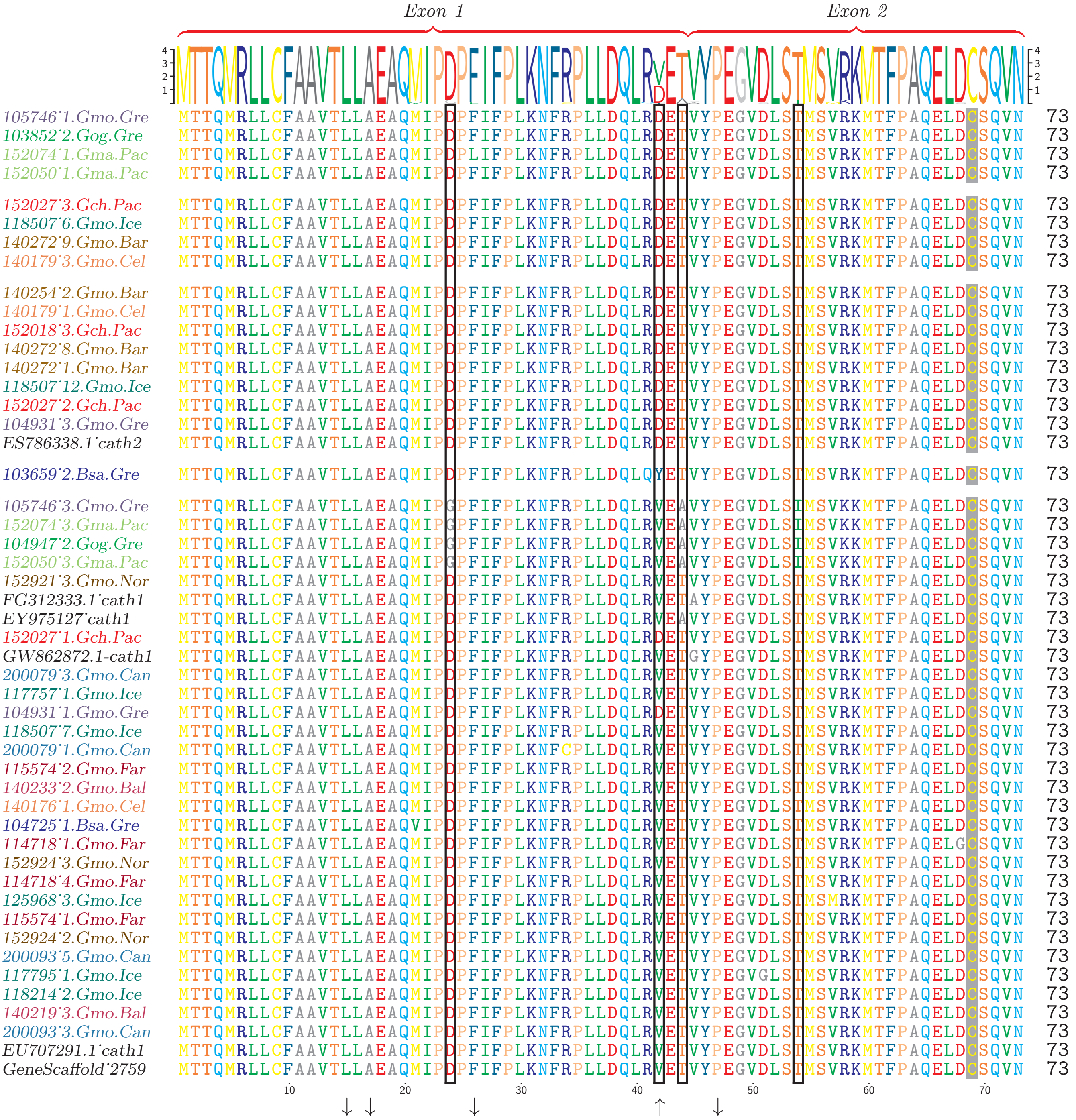

Exon 1
Exon 2
105746˙1.Gmo.Gre 73
103852˙2.Gog.Gre 73
152074˙1.Gma.Pac 73
152050˙1.Gma.Pac 73
152027˙3.Gch.Pac 73
118507˙6.Gmo.Ice 73
140272˙9.Gmo.Bar 73
140179˙3.Gmo.Cel 73
140254˙2.Gmo.Bar 73
140179˙1.Gmo.Cel 73
152018˙3.Gch.Pac 73
140272˙8.Gmo.Bar 73
140272˙1.Gmo.Bar 73
118507˙12.Gmo.Ice 73
152027˙2.Gch.Pac 73
104931˙3.Gmo.Gre 73
ES786338.1˙cath2 73
103659˙2.Bsa.Gre 73
105746˙3.Gmo.Gre 73
152074˙3.Gma.Pac 73
104947˙2.Gog.Gre 73
152050˙3.Gma.Pac 73
152921˙3.Gmo.Nor 73
FG312333.1˙cath1 73
EY975127˙cath1 73
152027˙1.Gch.Pac 73
GW862872.1-cath1 73
200079˙3.Gmo.Can 73
117757˙1.Gmo.Ice 73
104931˙1.Gmo.Gre 73
118507˙7.Gmo.Ice 73
200079˙1.Gmo.Can 73
115574˙2.Gmo.Far 73
140233˙2.Gmo.Bal 73
140176˙1.Gmo.Cel 73
104725˙1.Bsa.Gre 73
114718˙1.Gmo.Far 73
152924˙3.Gmo.Nor 73
114718˙4.Gmo.Far 73
125968˙3.Gmo.Ice 73
115574˙1.Gmo.Far 73
152924˙2.Gmo.Nor 73
200093˙5.Gmo.Can 73
117795˙1.Gmo.Ice 73
118214˙2.Gmo.Ice 73
140219˙3.Gmo.Bal 73
200093˙3.Gmo.Can 73
EU707291.1˙cath1 73
GeneScaffold˙2759 73
10 20 30 40 50 60 70

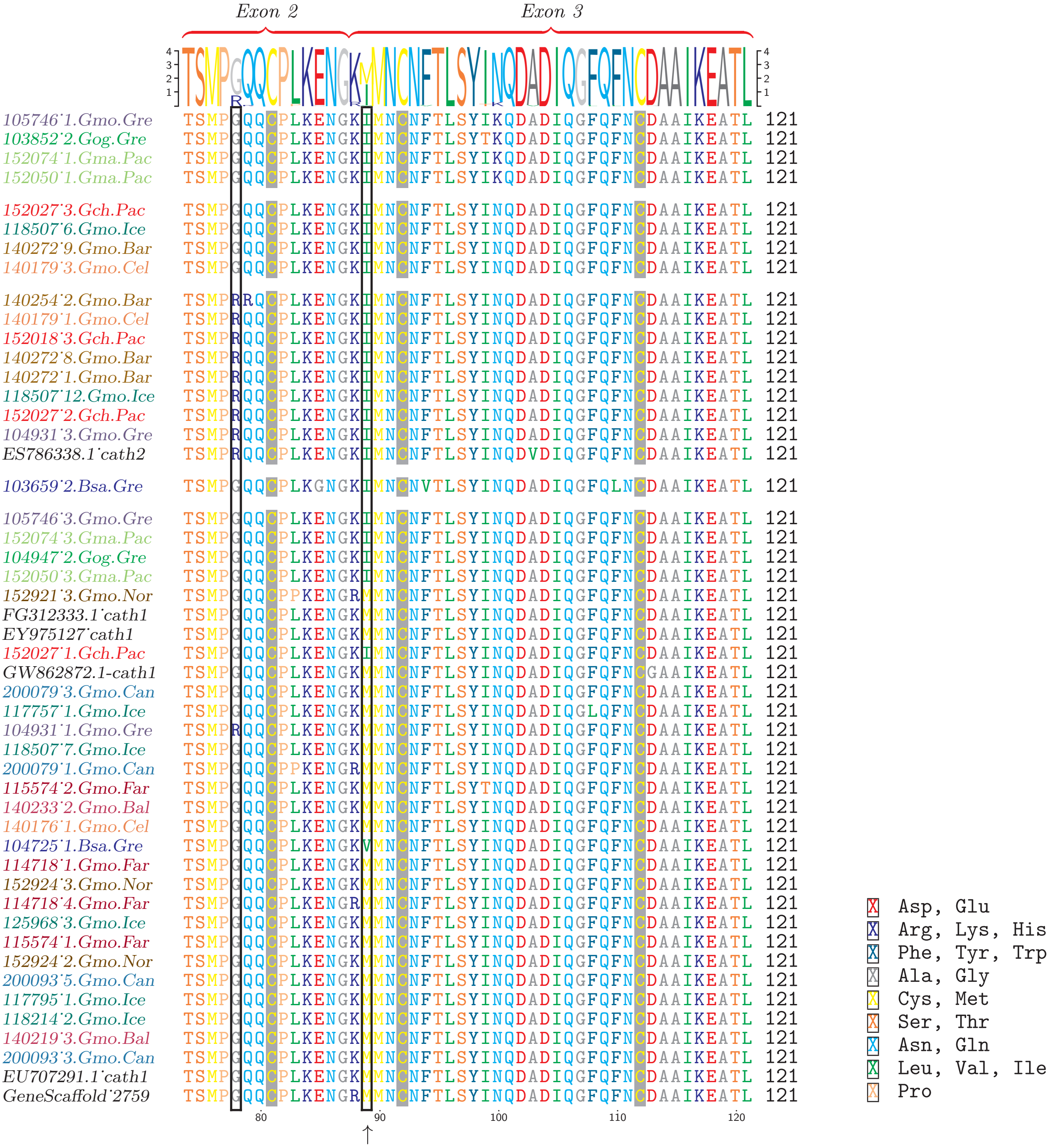

Exon 2
Exon 3
TSMPGQQCPLKENGKMMNCNFTLSYINQDADIQGFQFNCDAAIKEATL
105746˙1.Gmo.Gre TSMPGQQCPLKENGKIMNCNFTLSYIKQDADIQGFQFNCDAAIKEATL 121
103852˙2.Gog.Gre TSMPGQQCPLKENGKIMNCNFTLSYTKQDADIQGFQFNCDAAIKEATL 121
152074˙1.Gma.Pac TSMPGQQCPLKENGKIMNCNFTLSYIKQDADIQGFQFNCDAAIKEATL 121
152050˙1.Gma.Pac TSMPGQQCPLKENGKIMNCNFTLSYIKQDADIQGFQFNCDAAIKEATL 121
152027˙3.Gch.Pac TSMPGQQCPLKENGKIMNCNFTLSYINQDADIQGFQFNCDAAIKEATL 121
118507˙6.Gmo.Ice TSMPGQQCPLKENGKIMNCNFTLSYINQDADIQGFQFNCDAAIKEATL 121
140272˙9.Gmo.Bar TSMPGQQCPLKENGKIMNCNFTLSYINQDADIQGFQFNCDAAIKEATL 121
140179˙3.Gmo.Cel TSMPGQQCPLKENGKIMNCNFTLSYINQDADIQGFQFNCDAAIKEATL 121
140254˙2.Gmo.Bar TSMPRRQCPLKENGKIMNCNFTLSYINQDADIQGFQFNCDAAIKEATL 121
140179˙1.Gmo.Cel TSMPRQQCPLKENGKIMNCNFTLSYINQDADIQGFQFNCDAAIKEATL 121
152018˙3.Gch.Pac TSMPRQQCPLKENGKIMNCNFTLSYINQDADIQGFQFNCDAAIKEATL 121
140272˙8.Gmo.Bar TSMPRQQCPLKENGKIMNCNFTLSYINQDADIQGFQFNCDAAIKEATL 121
140272˙1.Gmo.Bar TSMPRQQCPLKENGKIMNCNFTLSYINQDADIQGFQFNCDAAIKEATL 121
118507˙12.Gmo.Ice TSMPRQQCPLKENGKIMNCNFTLSYINQDADIQGFQFNCDAAIKEATL 121
152027˙2.Gch.Pac TSMPRQQCPLKENGKIMNCNFTLSYINQDADIQGFQFNCDAAIKEATL 121
104931˙3.Gmo.Gre TSMPRQQCPLKENGKIMNCNFTLSYINQDADIQGFQFNCDAAIKEATL 121
ES786338.1˙cath2 TSMPRQQCPLKENGKIMNCNFTLSYINQDVDIQGFQFNCDAAIKEATL 121
103659˙2.Bsa.Gre TSMPGQQCPLKGNGKIMNCNVTLSYINQDADIQGFQLNCDAAIKEATL 121
105746˙3.Gmo.Gre TSMPGQQCPLKENGKIMNCNFTLSYINQDADIQGFQFNCDAAIKEATL 121
152074˙3.Gma.Pac TSMPGQQCPLKENGKIMNCNFTLSYINQDADIQGFQFNCDAAIKEATL 121
104947˙2.Gog.Gre TSMPGQQCPLKENGKIMNCNFTLSYINQDADIQGFQFNCDAAIKEATL 121
152050˙3.Gma.Pac TSMPGQQCPLKENGKIMNCNFTLSYINQDADIQGFQFNCDAAIKEATL 121
152921˙3.Gmo.Nor TSMPGQQCPPKENGRMMNCNFTLSYINQDADIQGFQFNCDAAIKEATL 121
FG312333.1˙cath1 TSMPGQQCPLKENGKMMNCNFTLSYINQDADIQGFQFNCDAAIKEATL 121
EY975127˙cath1 TSMPGQQCPLKENGKMMNCNFTLSYINQDADIQGFQFNCDAAIKEATL 121
152027˙1.Gch.Pac TSMPGQQCPLKENGKIMNCNFTLSYINQDADIQGFQFNCDAAIKEATL 121
GW862872.1-cath1 TSMPGQQCPLKENGKMMNCNFTLSYINQDADIQGFQFNCGAAIKEATL 121
200079˙3.Gmo.Can TSMPGQQCPLKENGKMMNCNFTLSYINQDADIQGFQFNCDAAIKEATL 121
117757˙1.Gmo.Ice TSMPGQQCPLKENGKMMNCNFTLSYINQDADIQGLQFNCDAAIKEATL 121
104931˙1.Gmo.Gre TSMPRQQCPLKENGKMMNCNFTLSYINQDADIQGFQFNCDAAIKEATL 121
118507˙7.Gmo.Ice TSMPGQQCPLKENGKMMNCNFTLSYINQDADIQGFQFNCDAAIKEATL 121
200079˙1.Gmo.Can TSMPGQQCPPKENGRMMNCNFTLSYINQDADIQGFQFNCDAAIKEATL 121
115574˙2.Gmo.Far TSMPGQQCPLKENGKMMNCNFTLSYTNQDADIQGFQFNCDAAIKEATL 121
140233˙2.Gmo.Bal TSMPGQQCPLKENGKMMNCNFTLSYINQDADIQGFQFNCDAAIKEATL 121
140176˙1.Gmo.Cel TSMPGQQCPLKENGKMMNCNFTLSYINQDADIQGFQFNCDAAIKEATL 121
104725˙1.Bsa.Gre TSMPGQQCPLKENGKVMNCNFTLSYINQDADIQGFQFNCDAAIKEATL 121
114718˙1.Gmo.Far TSMPGQQCPLKENGKMMNCNFTLSYINQDADIQGFQFNCDAAIKEATL 121
152924˙3.Gmo.Nor TSMPGQQCPLKENGKMMNCNFTLSYINQDADIQGFQFNCDAAIKEATL 121
114718˙4.Gmo.Far TSMPGQQCPLKENGRMMNCNFTLSYINQDADIQGFQFNCDAAIKEATL 121
125968˙3.Gmo.Ice TSMPGQQCPLKENGKMMNCNFTLSYINQDADIQGFQFNCDAAIKEATL 121
115574˙1.Gmo.Far TSMPGQQCPLKENGKMMNCNFTLSYINQDADIQGFQFNCDAAIKEATL 121
152924˙2.Gmo.Nor TSMPGQQCPLKENGKMMNCNFTLSYINQDADIQGFQFNCDAAIKEATL 121
200093˙5.Gmo.Can TSMPGQQCPLKENGKMMNCNFTLSYINQDADIQGFQFNCDAAIKEATL 121
117795˙1.Gmo.Ice TSMPGQQCPLKENGKMMNCNFTLSYINQDADIQGFQFNCDAAIKEATL 121
118214˙2.Gmo.Ice TSMPGQQCPLKENGKMMNCNFTLSYINQDADIQGFQFNCDAAIKEATL 121
140219˙3.Gmo.Bal TSMPGQQCPLKENGKMMNCNFTLSYINQDADIQGFQFNCDAAIKEATL 121
200093˙3.Gmo.Can TSMPGQQCPLKENGKMMNCNFTLSYINQDADIQGFQFNCDAAIKEATL 121
EU707291.1˙cath1 TSMPGQQCPLKENGKMMNCNFTLSYINQDADIQGFQFNCDAAIKEATL 121
GeneScaffold˙2759 TSMPGQQCPLKENGRMMNCNFTLSYINQDADIQGFQFNCDAAIKEATL 121
80
90
100
110
120
X Asp, Glu
X Arg, Lys, His
X Phe, Tyr, Trp
X Ala, Gly
X Cys, Met
X Ser, Thr
X Asn, Gln
X Leu, Val, Ile
X Pro
