# Supplementary material for: Trans-species polymorphism at antimicrobial innate immunity cathelicidin genes of Atlantic cod and related species
Source: PeerJ. 2015 May 21;3:e976. doi: 10.7717/peerj.976 (PMC4451034; doi:10.7717/peerj.976)
Supplement: Figure S2 — Bsa.Gre (Boreogadus saida), Gch.Pac (Gadus chalcogrammus), Gma.Pac (Gadus macrocephalus), Gog.Gre (Gadus ogac) and Gmo (Gadus morhua) from various locations; Iceland (Gmo.Ice), Greenland (Gmo.Gre), Barents Sea (Gmo.Bar), Celtic Sea (Gmo.Cel), Baltic Sea (Gmo.Bal), Norway (Gmo.Nor), Faeroe Islands (Gmo.Far), Canada (Gmo.Can). [file peerj-03-976-s003.pdf]

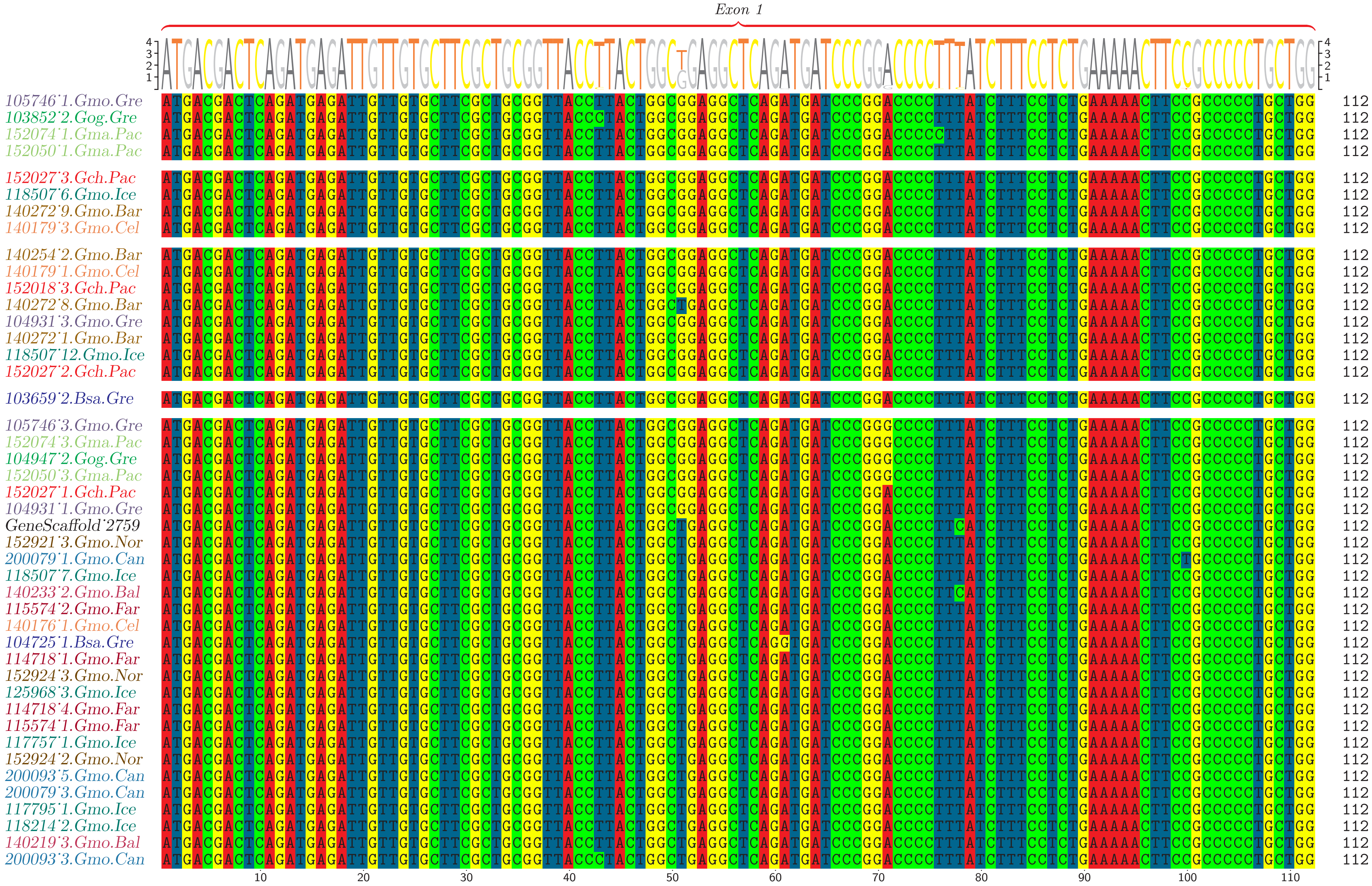
Exon 1
105746˙1.Gmo.Gre
103852˙2.Gog.Gre
152074˙1.Gma.Pac
152050˙1.Gma.Pac
152027˙3.Gch.Pac
118507˙6.Gmo.Ice
140272˙9.Gmo.Bar
140179˙3.Gmo.Cel
140254˙2.Gmo.Bar
140179˙1.Gmo.Cel
152018˙3.Gch.Pac
140272˙8.Gmo.Bar
104931˙3.Gmo.Gre
140272˙1.Gmo.Bar
118507˙12.Gmo.Ice
152027˙2.Gch.Pac
103659˙2.Bsa.Gre
105746˙3.Gmo.Gre
152074˙3.Gma.Pac
104947˙2.Gog.Gre
152050˙3.Gma.Pac
152027˙1.Gch.Pac
104931˙1.Gmo.Gre
GeneScaffold˙2759
152921˙3.Gmo.Nor
200079˙1.Gmo.Can
118507˙7.Gmo.Ice
140233˙2.Gmo.Bal
115574˙2.Gmo.Far
140176˙1.Gmo.Cel
104725˙1.Bsa.Gre
114718˙1.Gmo.Far
152924˙3.Gmo.Nor
125968˙3.Gmo.Ice
114718˙4.Gmo.Far
115574˙1.Gmo.Far
117757˙1.Gmo.Ice
152924˙2.Gmo.Nor
200093˙5.Gmo.Can
200079˙3.Gmo.Can
117795˙1.Gmo.Ice
118214˙2.Gmo.Ice
140219˙3.Gmo.Bal
200093˙3.Gmo.Can
112
10
20
30
40
50
60
70
80
90
100
110

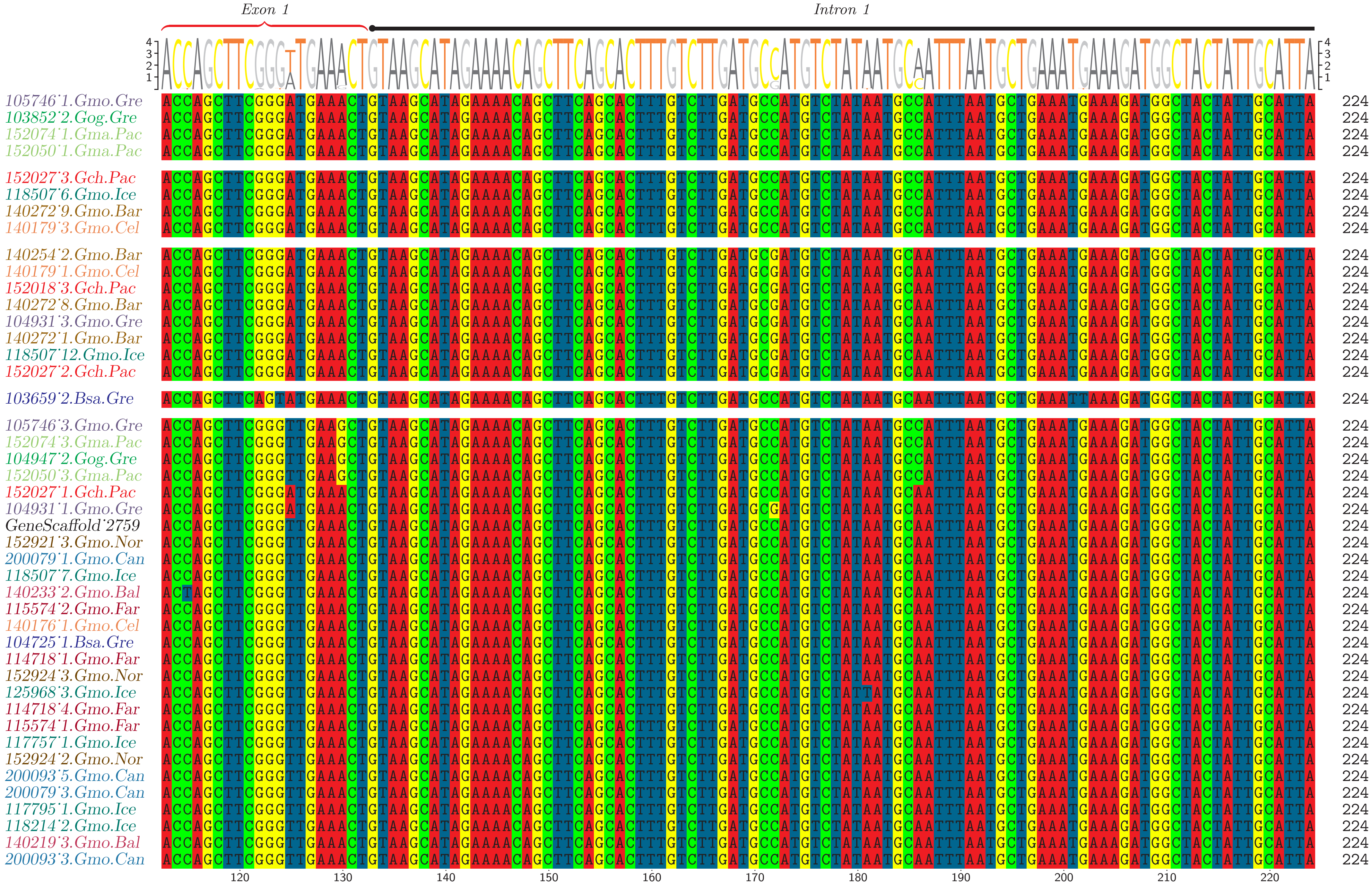

Exon 1
Intron 1
105746˙1.Gmo.Gre
103852˙2.Gog.Gre
152074˙1.Gma.Pac
152050˙1.Gma.Pac
152027˙3.Gch.Pac
118507˙6.Gmo.Ice
140272˙9.Gmo.Bar
140179˙3.Gmo.Cel
140254˙2.Gmo.Bar
140179˙1.Gmo.Cel
152018˙3.Gch.Pac
140272˙8.Gmo.Bar
104931˙3.Gmo.Gre
140272˙1.Gmo.Bar
118507˙12.Gmo.Ice
152027˙2.Gch.Pac
103659˙2.Bsa.Gre
105746˙3.Gmo.Gre
152074˙3.Gma.Pac
104947˙2.Gog.Gre
152050˙3.Gma.Pac
152027˙1.Gch.Pac
104931˙1.Gmo.Gre
GeneScaffold˙2759
152921˙3.Gmo.Nor
200079˙1.Gmo.Can
118507˙7.Gmo.Ice
140233˙2.Gmo.Bal
115574˙2.Gmo.Far
140176˙1.Gmo.Cel
104725˙1.Bsa.Gre
114718˙1.Gmo.Far
152924˙3.Gmo.Nor
125968˙3.Gmo.Ice
114718˙4.Gmo.Far
115574˙1.Gmo.Far
117757˙1.Gmo.Ice
152924˙2.Gmo.Nor
200093˙5.Gmo.Can
200079˙3.Gmo.Can
117795˙1.Gmo.Ice
118214˙2.Gmo.Ice
140219˙3.Gmo.Bal
200093˙3.Gmo.Can
224
120
130
140
150
160
170
180
190
200
210
220

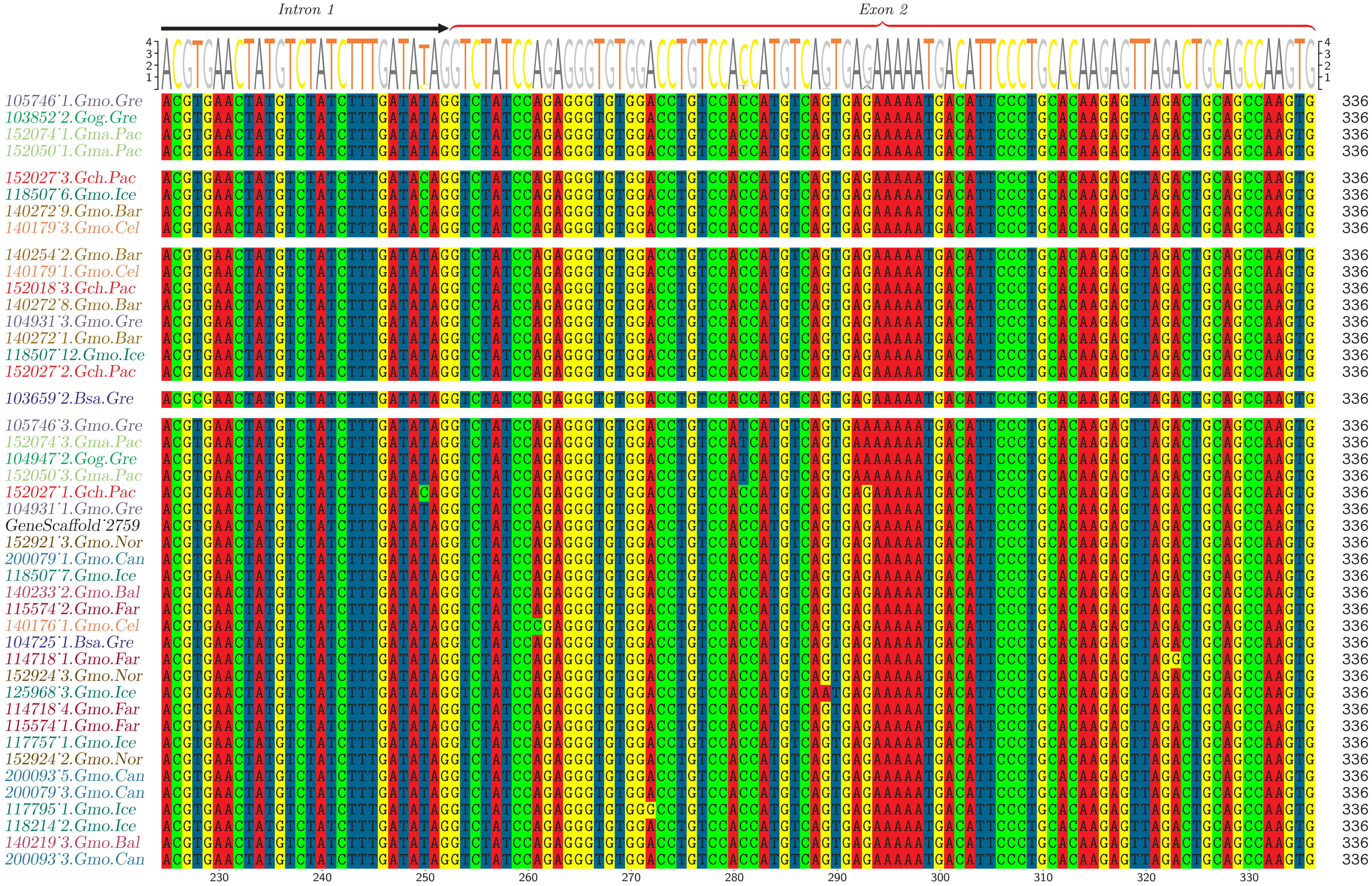

Intron 1
Exon 2
105746˙1.Gmo.Gre
103852˙2.Gog.Gre
152074˙1.Gma.Pac
152050˙1.Gma.Pac
152027˙3.Gch.Pac
118507˙6.Gmo.Ice
140272˙9.Gmo.Bar
140179˙3.Gmo.Cel
140254˙2.Gmo.Bar
140179˙1.Gmo.Cel
152018˙3.Gch.Pac
140272˙8.Gmo.Bar
104931˙3.Gmo.Gre
140272˙1.Gmo.Bar
118507˙12.Gmo.Ice
152027˙2.Gch.Pac
103659˙2.Bsa.Gre
105746˙3.Gmo.Gre
152074˙3.Gma.Pac
104947˙2.Gog.Gre
152050˙3.Gma.Pac
152027˙1.Gch.Pac
104931˙1.Gmo.Gre
GeneScaffold˙2759
152921˙3.Gmo.Nor
200079˙1.Gmo.Can
118507˙7.Gmo.Ice
140233˙2.Gmo.Bal
115574˙2.Gmo.Far
140176˙1.Gmo.Cel
104725˙1.Bsa.Gre
114718˙1.Gmo.Far
152924˙3.Gmo.Nor
125968˙3.Gmo.Ice
114718˙4.Gmo.Far
115574˙1.Gmo.Far
117757˙1.Gmo.Ice
152924˙2.Gmo.Nor
200093˙5.Gmo.Can
200079˙3.Gmo.Can
117795˙1.Gmo.Ice
118214˙2.Gmo.Ice
140219˙3.Gmo.Bal
200093˙3.Gmo.Can
336
230
240
250
260
270
280
290
300
310
320
330

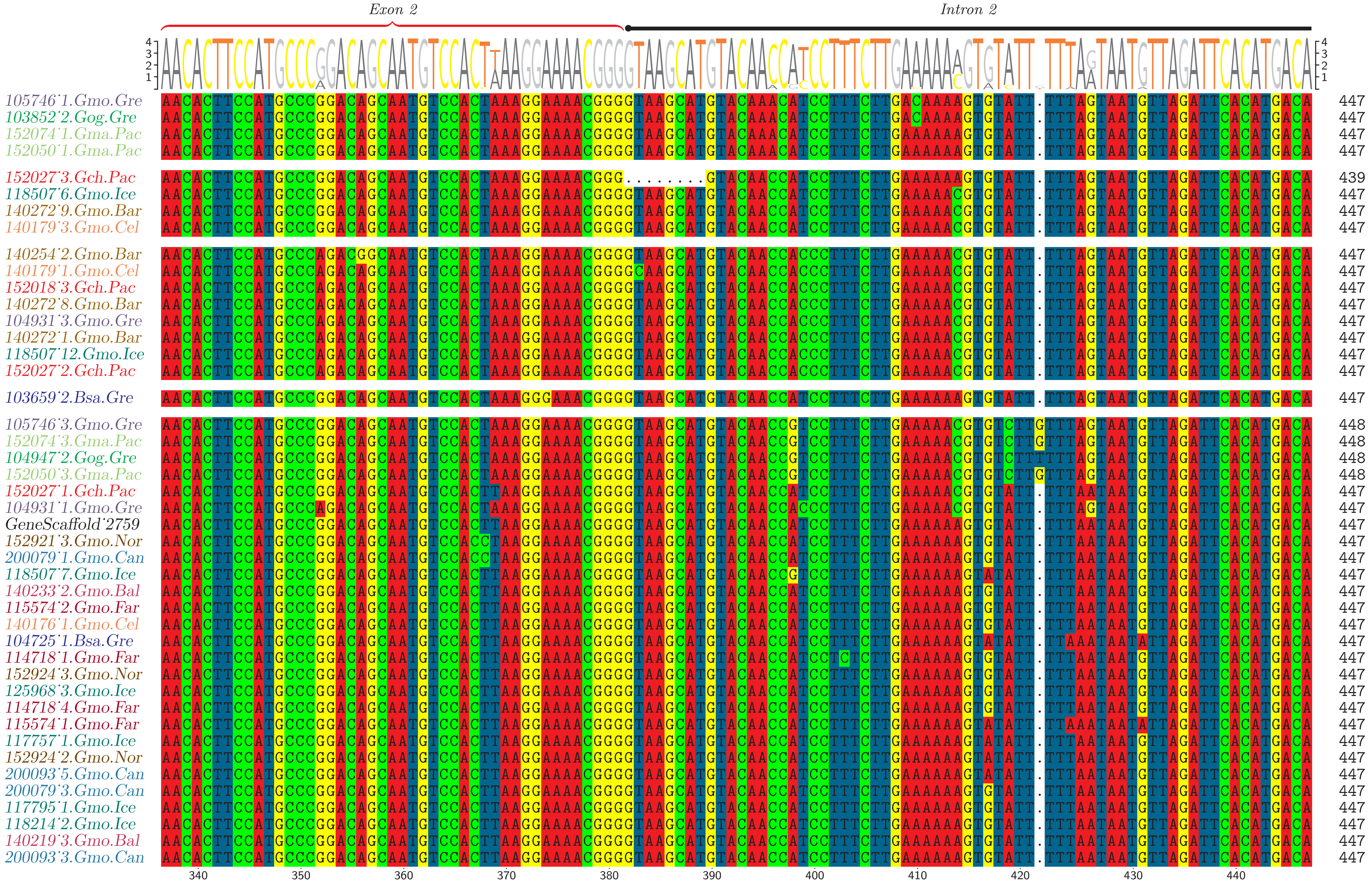

Exon 2
Intron 2
105746˙1.Gmo.Gre 447
103852˙2.Gog.Gre 447
152074˙1.Gma.Pac 447
152050˙1.Gma.Pac 447
152027˙3.Gch.Pac 439
118507˙6.Gmo.Ice 447
140272˙9.Gmo.Bar 447
140179˙3.Gmo.Cel 447
140254˙2.Gmo.Bar 447
140179˙1.Gmo.Cel 447
152018˙3.Gch.Pac 447
140272˙8.Gmo.Bar 447
104931˙3.Gmo.Gre 447
140272˙1.Gmo.Bar 447
118507˙12.Gmo.Ice 447
152027˙2.Gch.Pac 447
103659˙2.Bsa.Gre 447
105746˙3.Gmo.Gre 448
152074˙3.Gma.Pac 448
104947˙2.Gog.Gre 448
152050˙3.Gma.Pac 448
152027˙1.Gch.Pac 447
104931˙1.Gmo.Gre 447
GeneScaffold˙2759 447
152921˙3.Gmo.Nor 447
200079˙1.Gmo.Can 447
118507˙7.Gmo.Ice 447
140233˙2.Gmo.Bal 447
115574˙2.Gmo.Far 447
140176˙1.Gmo.Cel 447
104725˙1.Bsa.Gre 447
114718˙1.Gmo.Far 447
152924˙3.Gmo.Nor 447
125968˙3.Gmo.Ice 447
114718˙4.Gmo.Far 447
115574˙1.Gmo.Far 447
117757˙1.Gmo.Ice 447
152924˙2.Gmo.Nor 447
200093˙5.Gmo.Can 447
200079˙3.Gmo.Can 447
117795˙1.Gmo.Ice 447
118214˙2.Gmo.Ice 447
140219˙3.Gmo.Bal 447
200093˙3.Gmo.Can 447
340 350 360 370 380 390 400 410 420 430 440

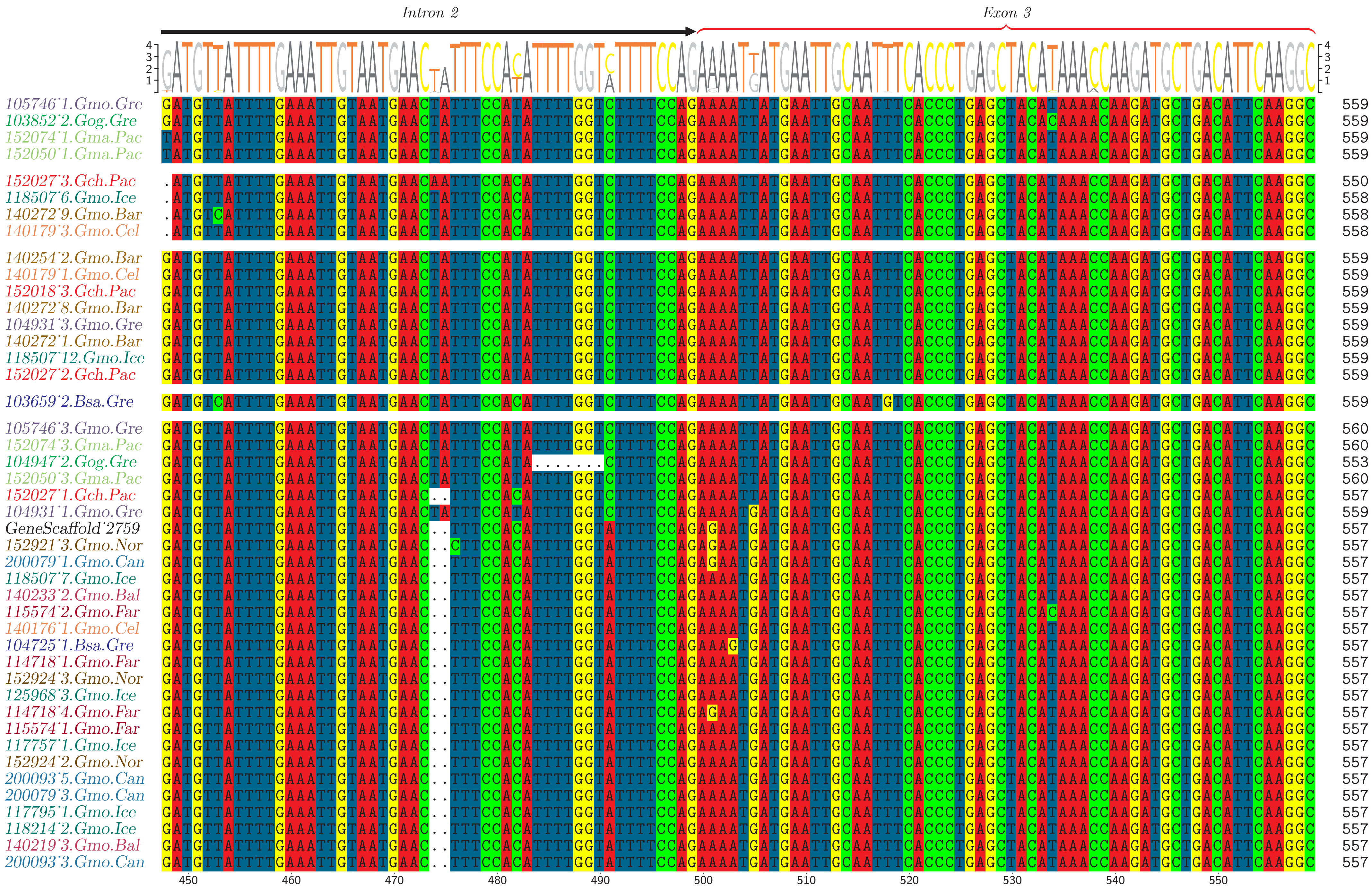
Intron 2
Exon 3
105746˙1.Gmo.Gre 559
103852˙2.Gog.Gre 559
152074˙1.Gma.Pac 559
152050˙1.Gma.Pac 559
152027˙3.Gch.Pac 550
118507˙6.Gmo.Ice 558
140272˙9.Gmo.Bar 558
140179˙3.Gmo.Cel 558
140254˙2.Gmo.Bar 559
140179˙1.Gmo.Cel 559
152018˙3.Gch.Pac 559
140272˙8.Gmo.Bar 559
104931˙3.Gmo.Gre 559
140272˙1.Gmo.Bar 559
118507˙12.Gmo.Ice 559
152027˙2.Gch.Pac 559
103659˙2.Bsa.Gre 559
105746˙3.Gmo.Gre 560
152074˙3.Gma.Pac 560
104947˙2.Gog.Gre 553
152050˙3.Gma.Pac 560
152027˙1.Gch.Pac 557
104931˙1.Gmo.Gre 559
GeneScaffold˙2759 557
152921˙3.Gmo.Nor 557
200079˙1.Gmo.Can 557
118507˙7.Gmo.Ice 557
140233˙2.Gmo.Bal 557
115574˙2.Gmo.Far 557
140176˙1.Gmo.Cel 557
104725˙1.Bsa.Gre 557
114718˙1.Gmo.Far 557
152924˙3.Gmo.Nor 557
125968˙3.Gmo.Ice 557
114718˙4.Gmo.Far 557
115574˙1.Gmo.Far 557
117757˙1.Gmo.Ice 557
152924˙2.Gmo.Nor 557
200093˙5.Gmo.Can 557
200079˙3.Gmo.Can 557
117795˙1.Gmo.Ice 557
118214˙2.Gmo.Ice 557
140219˙3.Gmo.Bal 557
200093˙3.Gmo.Can 557
450 460 470 480 490 500 510 520 530 540 550

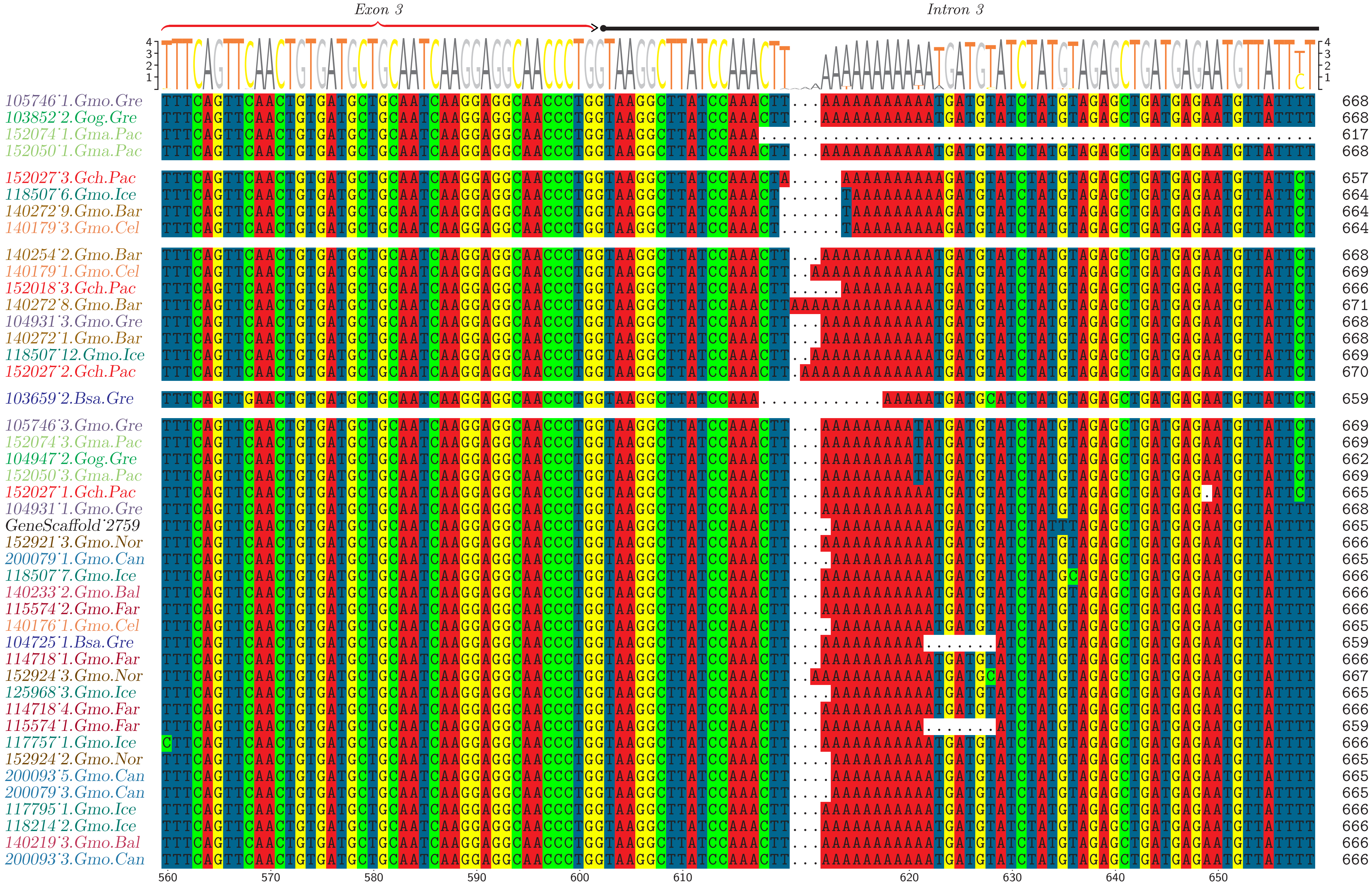
Exon 3
Intron 3
105746˙1.Gmo.Gre 668
103852˙2.Gog.Gre 668
152074˙1.Gma.Pac 617
152050˙1.Gma.Pac 668
152027˙3.Gch.Pac 657
118507˙6.Gmo.Ice 664
140272˙9.Gmo.Bar 664
140179˙3.Gmo.Cel 664
140254˙2.Gmo.Bar 668
140179˙1.Gmo.Cel 669
152018˙3.Gch.Pac 666
140272˙8.Gmo.Bar 671
104931˙3.Gmo.Gre 668
140272˙1.Gmo.Bar 668
118507˙12.Gmo.Ice 669
152027˙2.Gch.Pac 670
103659˙2.Bsa.Gre 659
105746˙3.Gmo.Gre 669
152074˙3.Gma.Pac 669
104947˙2.Gog.Gre 662
152050˙3.Gma.Pac 669
152027˙1.Gch.Pac 665
104931˙1.Gmo.Gre 668
GeneScaffold˙2759 665
152921˙3.Gmo.Nor 666
200079˙1.Gmo.Can 665
118507˙7.Gmo.Ice 666
140233˙2.Gmo.Bal 666
115574˙2.Gmo.Far 666
140176˙1.Gmo.Cel 665
104725˙1.Bsa.Gre 659
114718˙1.Gmo.Far 666
152924˙3.Gmo.Nor 667
125968˙3.Gmo.Ice 665
114718˙4.Gmo.Far 666
115574˙1.Gmo.Far 659
117757˙1.Gmo.Ice 666
152924˙2.Gmo.Nor 665
200093˙5.Gmo.Can 665
200079˙3.Gmo.Can 665
117795˙1.Gmo.Ice 666
118214˙2.Gmo.Ice 666
140219˙3.Gmo.Bal 666
200093˙3.Gmo.Can 666
560 570 580 590 600 610 620 630 640 650

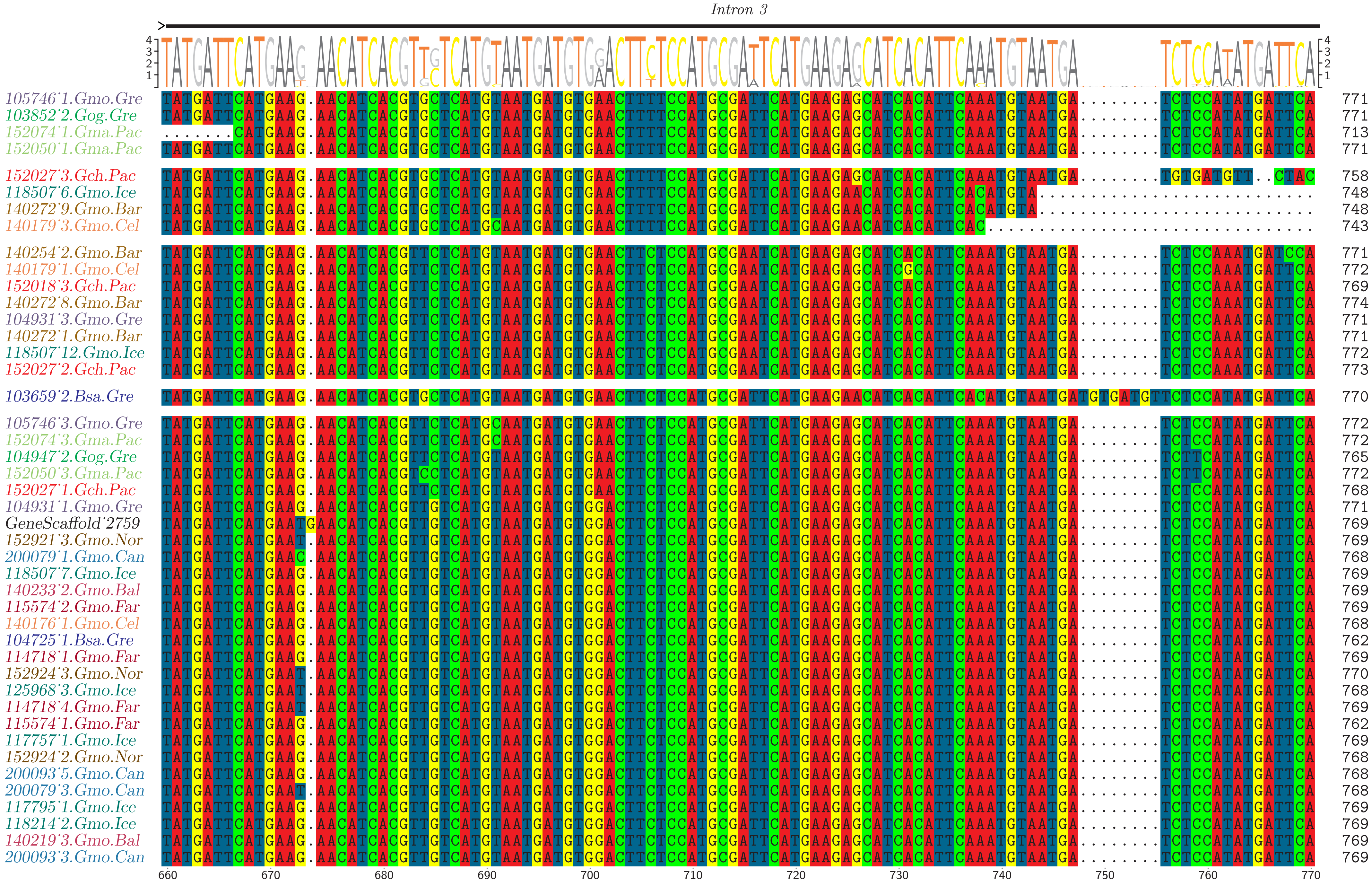

Intron 3
105746˙1.Gmo.Gre 771
103852˙2.Gog.Gre 771
152074˙1.Gma.Pac 713
152050˙1.Gma.Pac 771
152027˙3.Gch.Pac 758
118507˙6.Gmo.Ice 748
140272˙9.Gmo.Bar 748
140179˙3.Gmo.Cel 743
140254˙2.Gmo.Bar 771
140179˙1.Gmo.Cel 772
152018˙3.Gch.Pac 769
140272˙8.Gmo.Bar 774
104931˙3.Gmo.Gre 771
140272˙1.Gmo.Bar 771
118507˙12.Gmo.Ice 772
152027˙2.Gch.Pac 773
103659˙2.Bsa.Gre 770
105746˙3.Gmo.Gre 772
152074˙3.Gma.Pac 772
104947˙2.Gog.Gre 765
152050˙3.Gma.Pac 772
152027˙1.Gch.Pac 768
104931˙1.Gmo.Gre 771
GeneScaffold˙2759 769
152921˙3.Gmo.Nor 769
200079˙1.Gmo.Can 768
118507˙7.Gmo.Ice 769
140233˙2.Gmo.Bal 769
115574˙2.Gmo.Far 769
140176˙1.Gmo.Cel 768
104725˙1.Bsa.Gre 762
114718˙1.Gmo.Far 769
152924˙3.Gmo.Nor 770
125968˙3.Gmo.Ice 768
114718˙4.Gmo.Far 769
115574˙1.Gmo.Far 762
117757˙1.Gmo.Ice 769
152924˙2.Gmo.Nor 768
200093˙5.Gmo.Can 768
200079˙3.Gmo.Can 768
117795˙1.Gmo.Ice 769
118214˙2.Gmo.Ice 769
140219˙3.Gmo.Bal 769
200093˙3.Gmo.Can 769
660 670 680 690 700 710 720 730 740 750 760 770

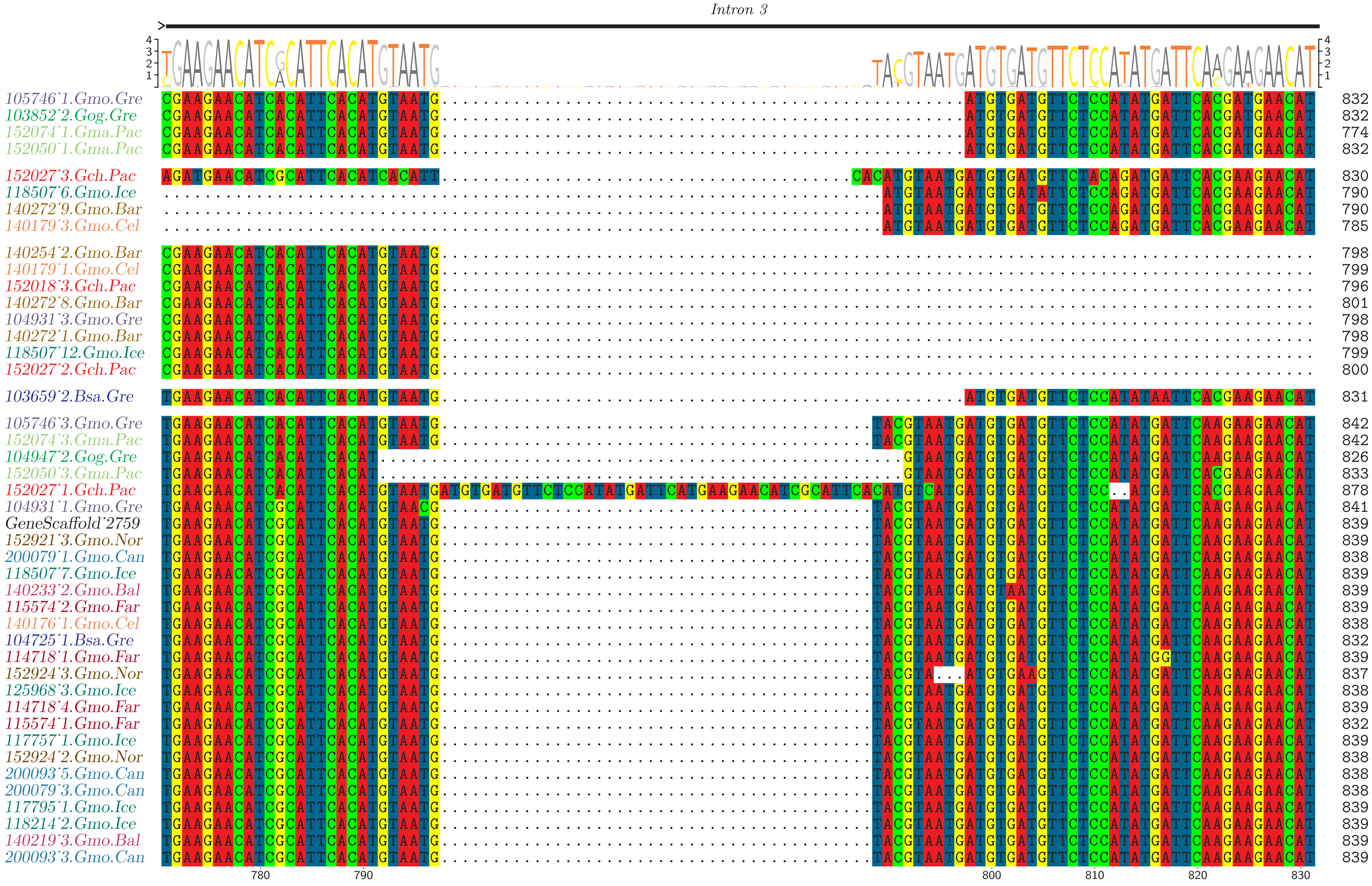

Intron 3
105746˙1.Gmo.Gre 832
103852˙2.Gog.Gre 832
152074˙1.Gma.Pac 774
152050˙1.Gma.Pac 832
152027˙3.Gch.Pac 830
118507˙6.Gmo.Ice 790
140272˙9.Gmo.Bar 790
140179˙3.Gmo.Cel 785
140254˙2.Gmo.Bar 798
140179˙1.Gmo.Cel 799
152018˙3.Gch.Pac 796
140272˙8.Gmo.Bar 801
104931˙3.Gmo.Gre 798
140272˙1.Gmo.Bar 798
118507˙12.Gmo.Ice 799
152027˙2.Gch.Pac 800
103659˙2.Bsa.Gre 831
105746˙3.Gmo.Gre 842
152074˙3.Gma.Pac 842
104947˙2.Gog.Gre 826
152050˙3.Gma.Pac 833
152027˙1.Gch.Pac 878
104931˙1.Gmo.Gre 841
GeneScaffold˙2759 839
152921˙3.Gmo.Nor 839
200079˙1.Gmo.Can 838
118507˙7.Gmo.Ice 839
140233˙2.Gmo.Bal 839
115574˙2.Gmo.Far 839
140176˙1.Gmo.Cel 838
104725˙1.Bsa.Gre 832
114718˙1.Gmo.Far 839
152924˙3.Gmo.Nor 837
125968˙3.Gmo.Ice 838
114718˙4.Gmo.Far 839
115574˙1.Gmo.Far 832
117757˙1.Gmo.Ice 839
152924˙2.Gmo.Nor 838
200093˙5.Gmo.Can 838
200079˙3.Gmo.Can 838
117795˙1.Gmo.Ice 839
118214˙2.Gmo.Ice 839
140219˙3.Gmo.Bal 839
200093˙3.Gmo.Can 839
780
790
800
810
820
830

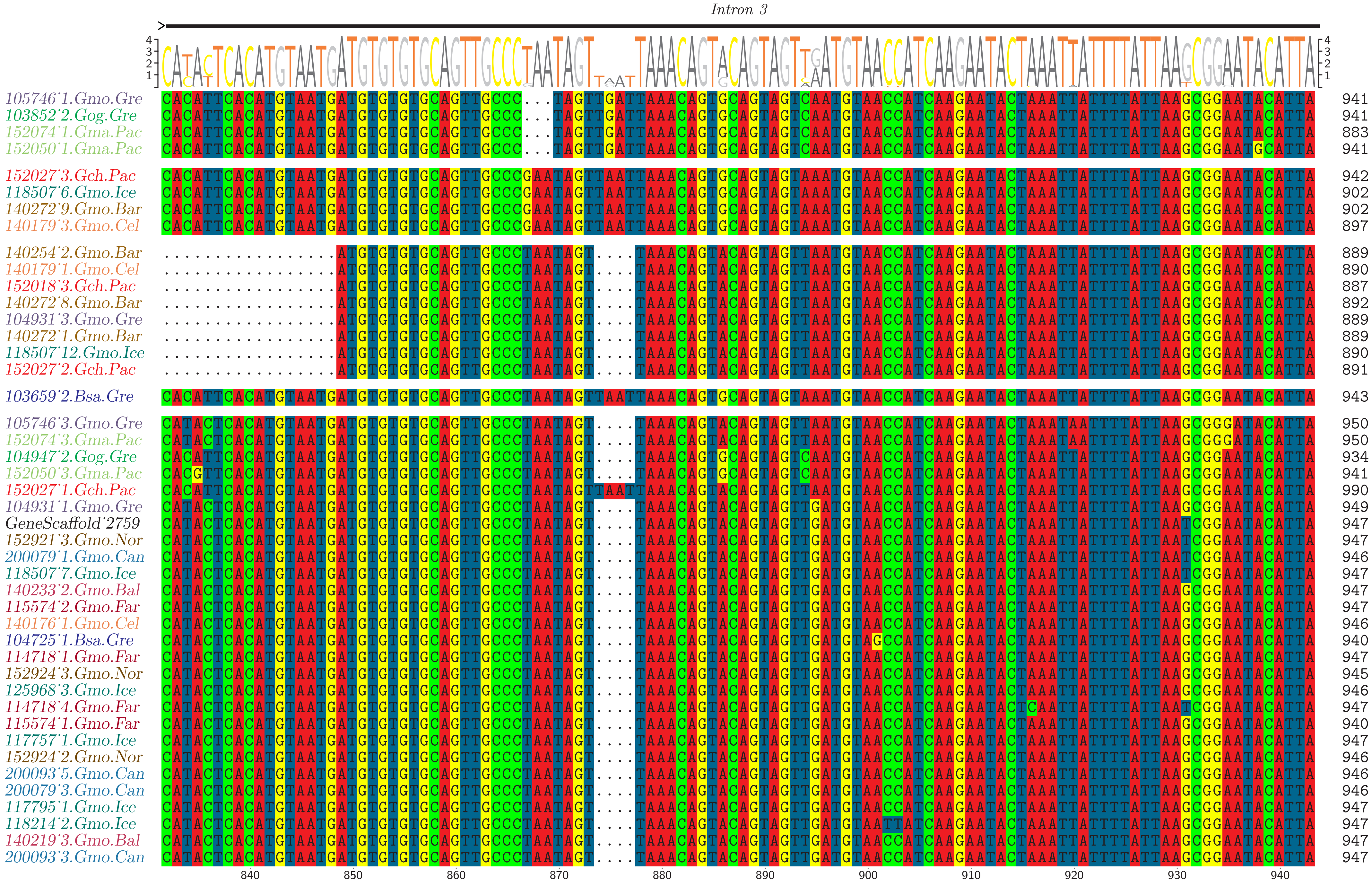
Intron 3
105746˙1.Gmo.Gre 941
103852˙2.Gog.Gre 941
152074˙1.Gma.Pac 883
152050˙1.Gma.Pac 941
152027˙3.Gch.Pac 942
118507˙6.Gmo.Ice 902
140272˙9.Gmo.Bar 902
140179˙3.Gmo.Cel 897
140254˙2.Gmo.Bar 889
140179˙1.Gmo.Cel 890
152018˙3.Gch.Pac 887
140272˙8.Gmo.Bar 892
104931˙3.Gmo.Gre 889
140272˙1.Gmo.Bar 889
118507˙12.Gmo.Ice 890
152027˙2.Gch.Pac 891
103659˙2.Bsa.Gre 943
105746˙3.Gmo.Gre 950
152074˙3.Gma.Pac 950
104947˙2.Gog.Gre 934
152050˙3.Gma.Pac 941
152027˙1.Gch.Pac 990
104931˙1.Gmo.Gre 949
GeneScaffold˙2759 947
152921˙3.Gmo.Nor 947
200079˙1.Gmo.Can 946
118507˙7.Gmo.Ice 947
140233˙2.Gmo.Bal 947
115574˙2.Gmo.Far 947
140176˙1.Gmo.Cel 946
104725˙1.Bsa.Gre 940
114718˙1.Gmo.Far 947
152924˙3.Gmo.Nor 945
125968˙3.Gmo.Ice 946
114718˙4.Gmo.Far 947
115574˙1.Gmo.Far 940
117757˙1.Gmo.Ice 947
152924˙2.Gmo.Nor 946
200093˙5.Gmo.Can 946
200079˙3.Gmo.Can 946
117795˙1.Gmo.Ice 947
118214˙2.Gmo.Ice 947
140219˙3.Gmo.Bal 947
200093˙3.Gmo.Can 947
840
850
860
870
880
890
900
910
920
930
940

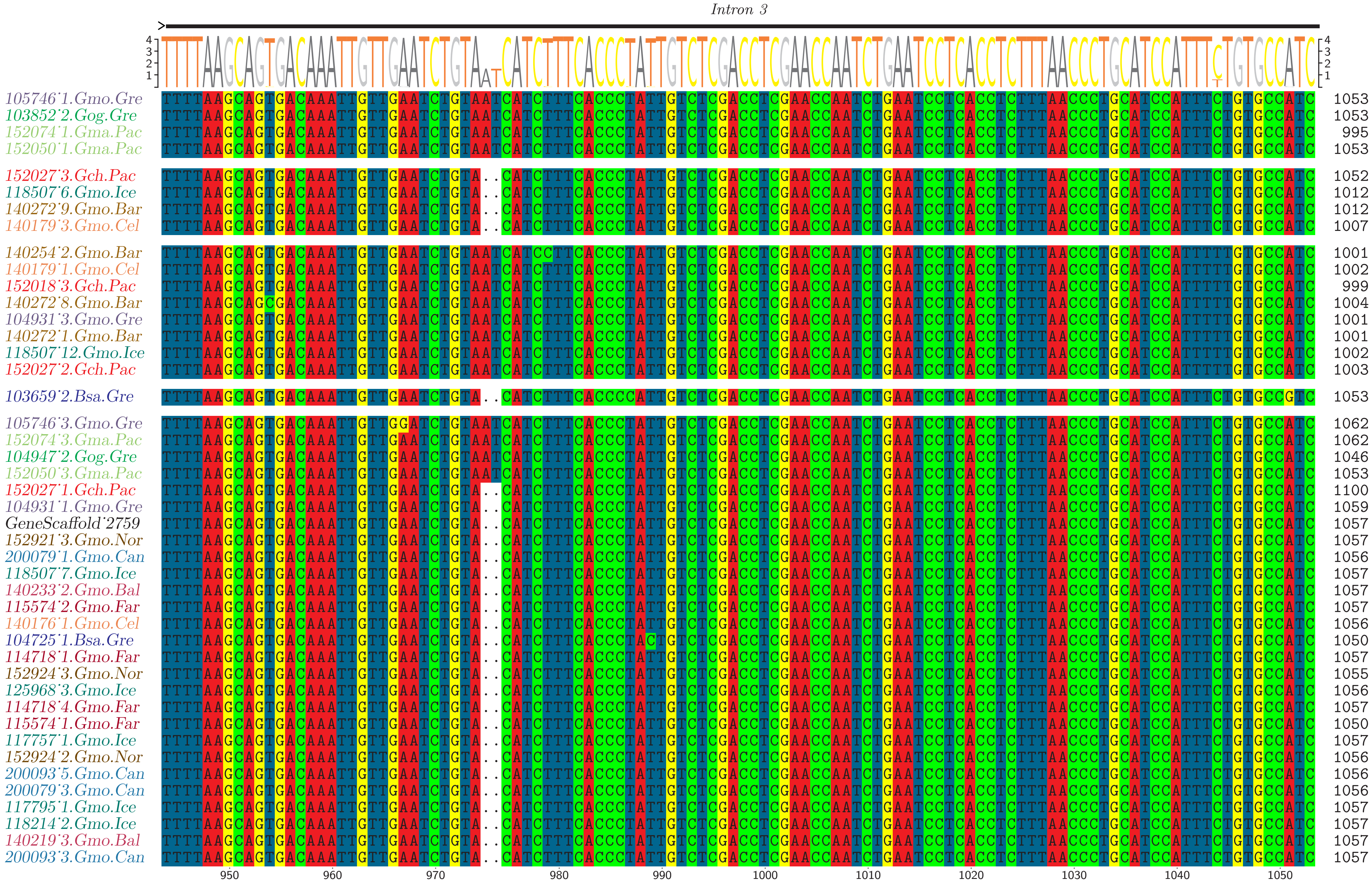

Intron 3
105746_1.Gmo.Gre 1053
103852_2.Gog.Gre 1053
152074_1.Gma.Pac 995
152050_1.Gma.Pac 1053
152027_3.Gch.Pac 1052
118507_6.Gmo.Ice 1012
140272_9.Gmo.Bar 1012
140179_3.Gmo.Cel 1007
140254_2.Gmo.Bar 1001
140179_1.Gmo.Cel 1002
152018_3.Gch.Pac 999
140272_8.Gmo.Bar 1004
104931_3.Gmo.Gre 1001
140272_1.Gmo.Bar 1001
118507_12.Gmo.Ice 1002
152027_2.Gch.Pac 1003
103659_2.Bsa.Gre 1053
105746_3.Gmo.Gre 1062
152074_3.Gma.Pac 1062
104947_2.Gog.Gre 1046
152050_3.Gma.Pac 1053
152027_1.Gch.Pac 1100
104931_1.Gmo.Gre 1059
GeneScaffold_2759 1057
152921_3.Gmo.Nor 1057
200079_1.Gmo.Can 1056
118507_7.Gmo.Ice 1057
140233_2.Gmo.Bal 1057
115574_2.Gmo.Far 1057
140176_1.Gmo.Cel 1056
104725_1.Bsa.Gre 1050
114718_1.Gmo.Far 1057
152924_3.Gmo.Nor 1055
125968_3.Gmo.Ice 1056
114718_4.Gmo.Far 1057
115574_1.Gmo.Far 1050
117757_1.Gmo.Ice 1057
152924_2.Gmo.Nor 1056
200093_5.Gmo.Can 1056
200079_3.Gmo.Can 1056
117795_1.Gmo.Ice 1057
118214_2.Gmo.Ice 1057
140219_3.Gmo.Bal 1057
200093_3.Gmo.Can 1057
950 960 970 980 990 1000 1010 1020 1030 1040 1050

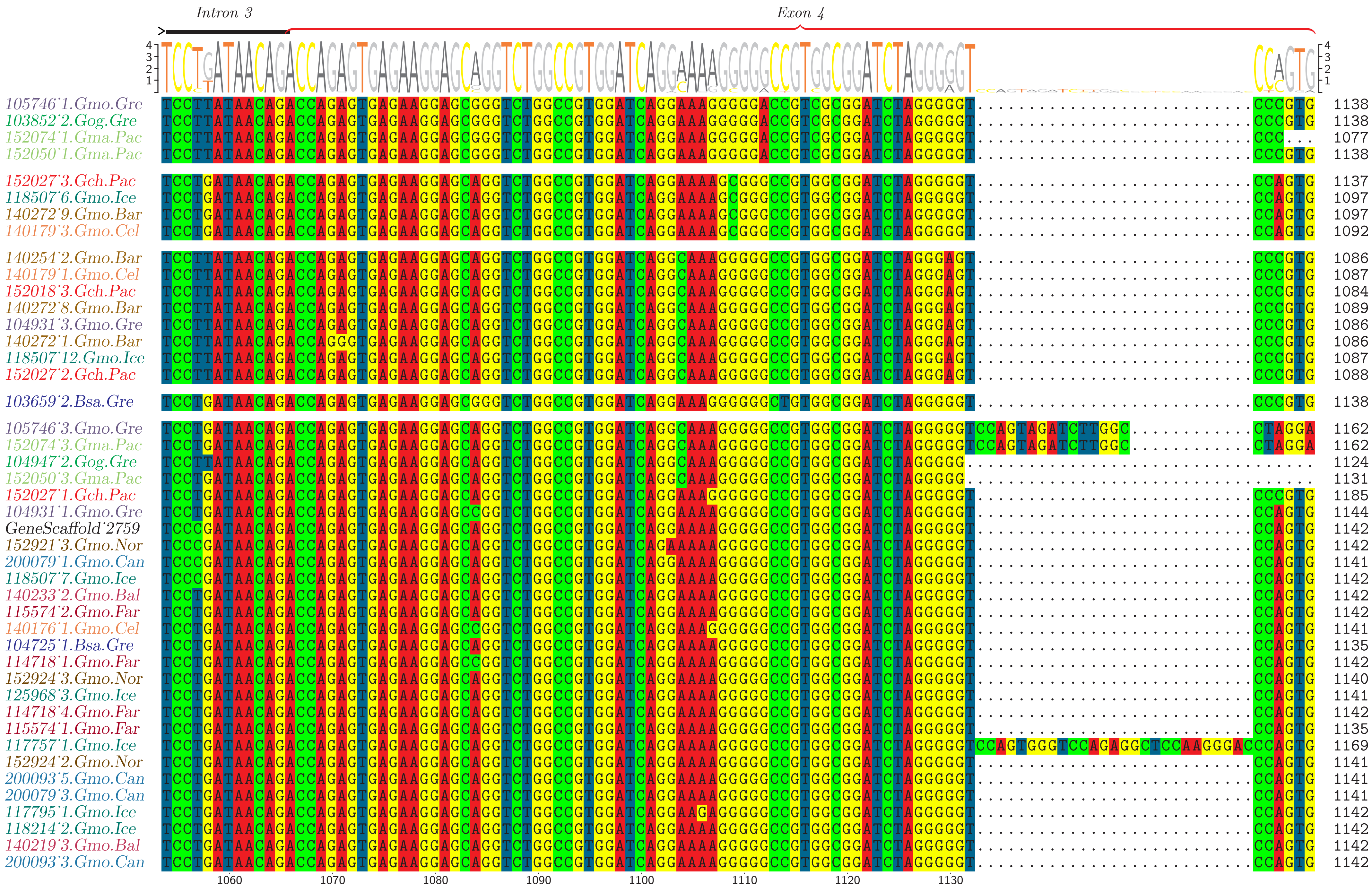

Intron 3
Exon 4
105746˙1.Gmo.Gre 1138
103852˙2.Gog.Gre 1138
152074˙1.Gma.Pac 1077
152050˙1.Gma.Pac 1138
152027˙3.Gch.Pac 1137
118507˙6.Gmo.Ice 1097
140272˙9.Gmo.Bar 1097
140179˙3.Gmo.Cel 1092
140254˙2.Gmo.Bar 1086
140179˙1.Gmo.Cel 1087
152018˙3.Gch.Pac 1084
140272˙8.Gmo.Bar 1089
104931˙3.Gmo.Gre 1086
140272˙1.Gmo.Bar 1086
118507˙12.Gmo.Ice 1087
152027˙2.Gch.Pac 1088
103659˙2.Bsa.Gre 1138
105746˙3.Gmo.Gre 1162
152074˙3.Gma.Pac 1162
104947˙2.Gog.Gre 1124
152050˙3.Gma.Pac 1131
152027˙1.Gch.Pac 1185
104931˙1.Gmo.Gre 1144
GeneScaffold˙2759 1142
152921˙3.Gmo.Nor 1142
200079˙1.Gmo.Can 1141
118507˙7.Gmo.Ice 1142
140233˙2.Gmo.Bal 1142
115574˙2.Gmo.Far 1142
140176˙1.Gmo.Cel 1141
104725˙1.Bsa.Gre 1135
114718˙1.Gmo.Far 1142
152924˙3.Gmo.Nor 1140
125968˙3.Gmo.Ice 1141
114718˙4.Gmo.Far 1142
115574˙1.Gmo.Far 1135
117757˙1.Gmo.Ice 1169
152924˙2.Gmo.Nor 1141
200093˙5.Gmo.Can 1141
200079˙3.Gmo.Can 1141
117795˙1.Gmo.Ice 1142
118214˙2.Gmo.Ice 1142
140219˙3.Gmo.Bal 1142
200093˙3.Gmo.Can 1142
1060 1070 1080 1090 1100 1110 1120 1130

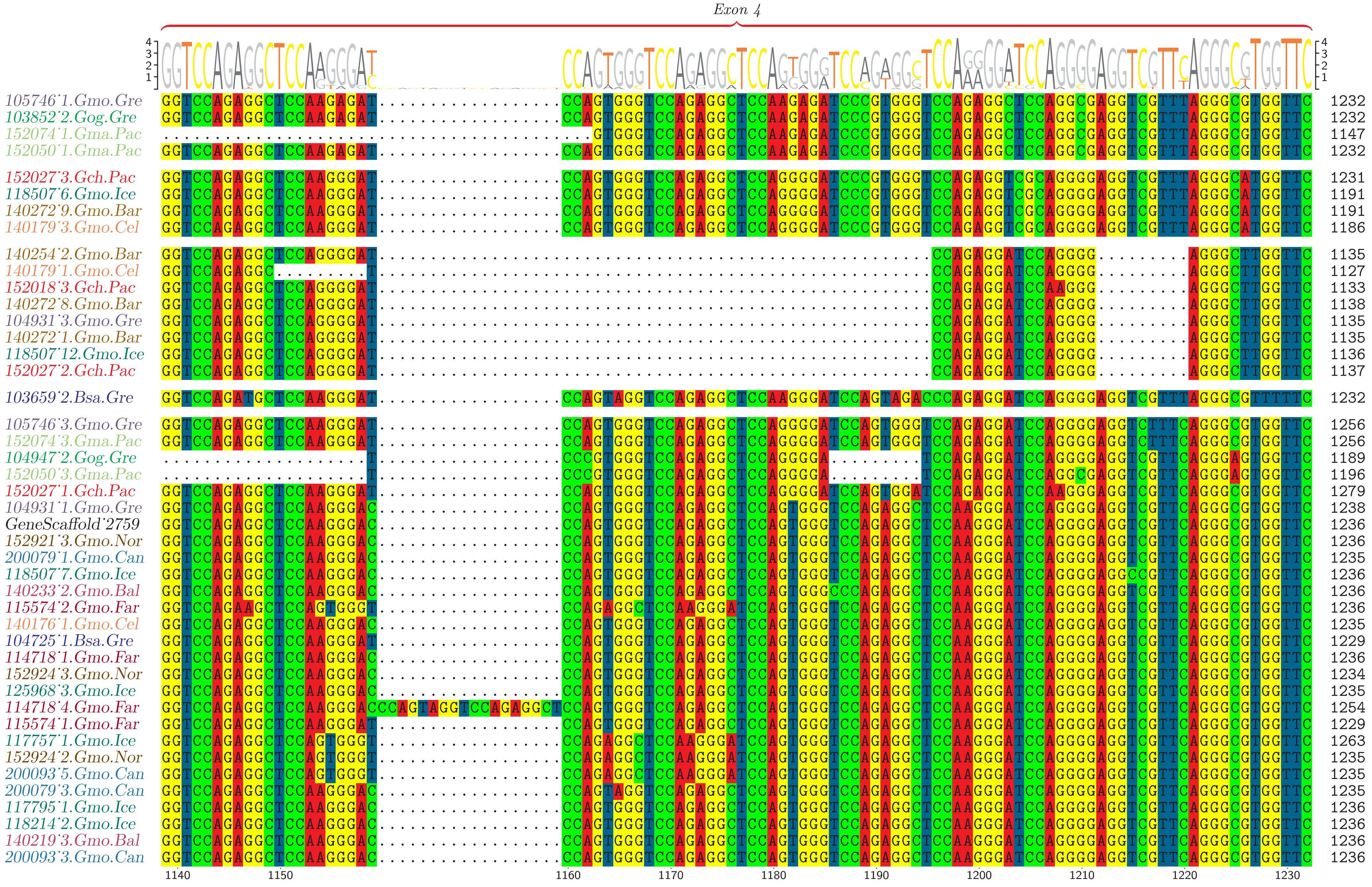

Exon 4
105746˙1.Gmo.Gre 1232
103852˙2.Gog.Gre 1232
152074˙1.Gma.Pac 1147
152050˙1.Gma.Pac 1232
152027˙3.Gch.Pac 1231
118507˙6.Gmo.Ice 1191
140272˙9.Gmo.Bar 1191
140179˙3.Gmo.Cel 1186
140254˙2.Gmo.Bar 1135
140179˙1.Gmo.Cel 1127
152018˙3.Gch.Pac 1133
140272˙8.Gmo.Bar 1138
104931˙3.Gmo.Gre 1135
140272˙1.Gmo.Bar 1135
118507˙12.Gmo.Ice 1136
152027˙2.Gch.Pac 1137
103659˙2.Bsa.Gre 1232
105746˙3.Gmo.Gre 1256
152074˙3.Gma.Pac 1256
104947˙2.Gog.Gre 1189
152050˙3.Gma.Pac 1196
152027˙1.Gch.Pac 1279
104931˙1.Gmo.Gre 1238
GeneScaffold˙2759 1236
152921˙3.Gmo.Nor 1236
200079˙1.Gmo.Can 1235
118507˙7.Gmo.Ice 1236
140233˙2.Gmo.Bal 1236
115574˙2.Gmo.Far 1236
140176˙1.Gmo.Cel 1235
104725˙1.Bsa.Gre 1229
114718˙1.Gmo.Far 1236
152924˙3.Gmo.Nor 1234
125968˙3.Gmo.Ice 1235
114718˙4.Gmo.Far 1254
115574˙1.Gmo.Far 1229
117757˙1.Gmo.Ice 1263
152924˙2.Gmo.Nor 1235
200093˙5.Gmo.Can 1235
200079˙3.Gmo.Can 1235
117795˙1.Gmo.Ice 1236
118214˙2.Gmo.Ice 1236
140219˙3.Gmo.Bal 1236
200093˙3.Gmo.Can 1236
1140 1150 1160 1170 1180 1190 1200 1210 1220 1230

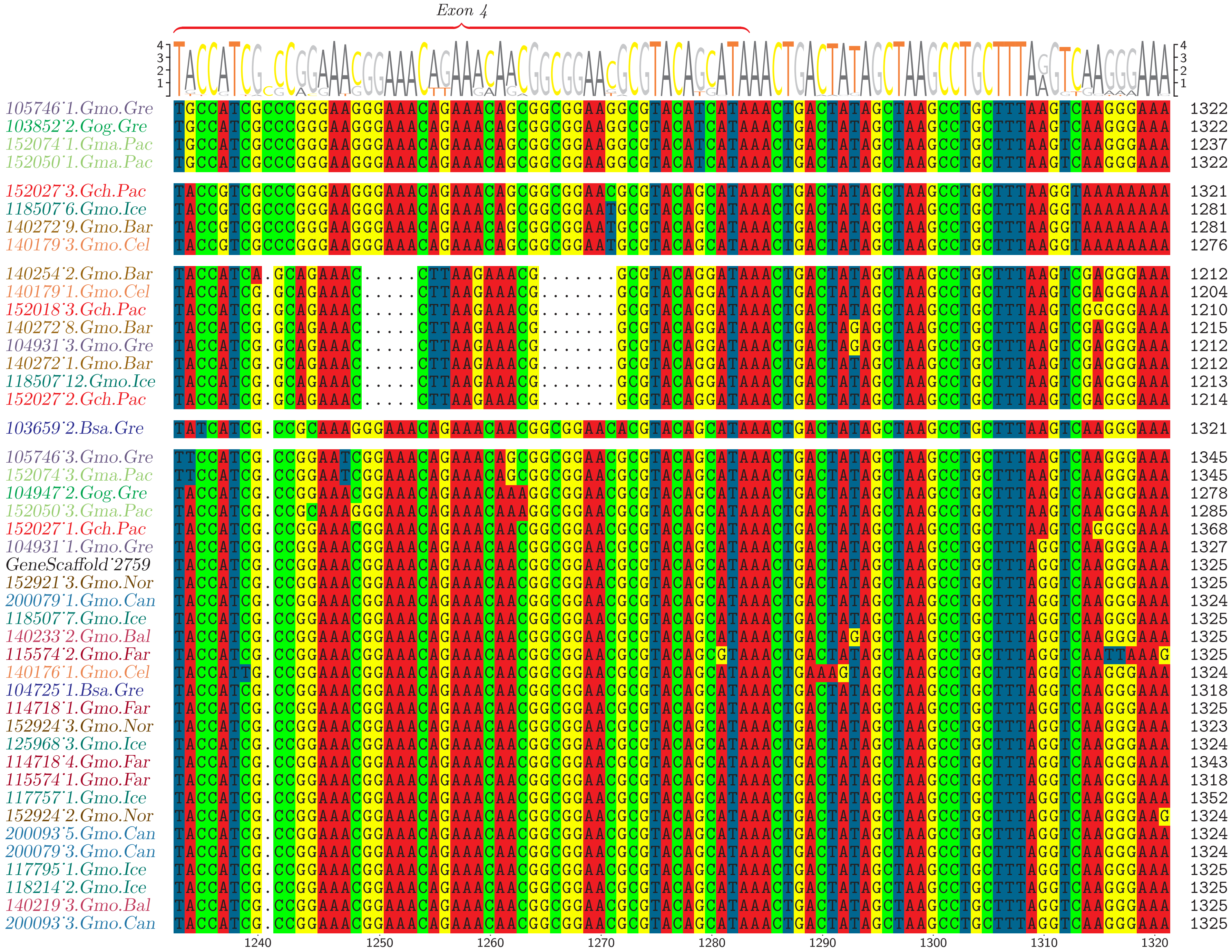
Exon 4
105746˙1.Gmo.Gre 1322
103852˙2.Gog.Gre 1322
152074˙1.Gma.Pac 1237
152050˙1.Gma.Pac 1322
152027˙3.Gch.Pac 1321
118507˙6.Gmo.Ice 1281
140272˙9.Gmo.Bar 1281
140179˙3.Gmo.Cel 1276
140254˙2.Gmo.Bar 1212
140179˙1.Gmo.Cel 1204
152018˙3.Gch.Pac 1210
140272˙8.Gmo.Bar 1215
104931˙3.Gmo.Gre 1212
140272˙1.Gmo.Bar 1212
118507˙12.Gmo.Ice 1213
152027˙2.Gch.Pac 1214
103659˙2.Bsa.Gre 1321
105746˙3.Gmo.Gre 1345
152074˙3.Gma.Pac 1345
104947˙2.Gog.Gre 1278
152050˙3.Gma.Pac 1285
152027˙1.Gch.Pac 1368
104931˙1.Gmo.Gre 1327
GeneScaffold˙2759 1325
152921˙3.Gmo.Nor 1325
200079˙1.Gmo.Can 1324
118507˙7.Gmo.Ice 1325
140233˙2.Gmo.Bal 1325
115574˙2.Gmo.Far 1325
140176˙1.Gmo.Cel 1324
104725˙1.Bsa.Gre 1318
114718˙1.Gmo.Far 1325
152924˙3.Gmo.Nor 1323
125968˙3.Gmo.Ice 1324
114718˙4.Gmo.Far 1343
115574˙1.Gmo.Far 1318
117757˙1.Gmo.Ice 1352
152924˙2.Gmo.Nor 1324
200093˙5.Gmo.Can 1324
200079˙3.Gmo.Can 1324
117795˙1.Gmo.Ice 1325
118214˙2.Gmo.Ice 1325
140219˙3.Gmo.Bal 1325
200093˙3.Gmo.Can 1325
1240
1250
1260
1270
1280
1290
1300
1310
1320
