# Supplementary figures and images for: Trans-species polymorphism at antimicrobial innate immunity cathelicidin genes of Atlantic cod and related species
Source: PeerJ. 2015 May 21;3:e976. doi: 10.7717/peerj.976 (PMC4451034; doi:10.7717/peerj.976)

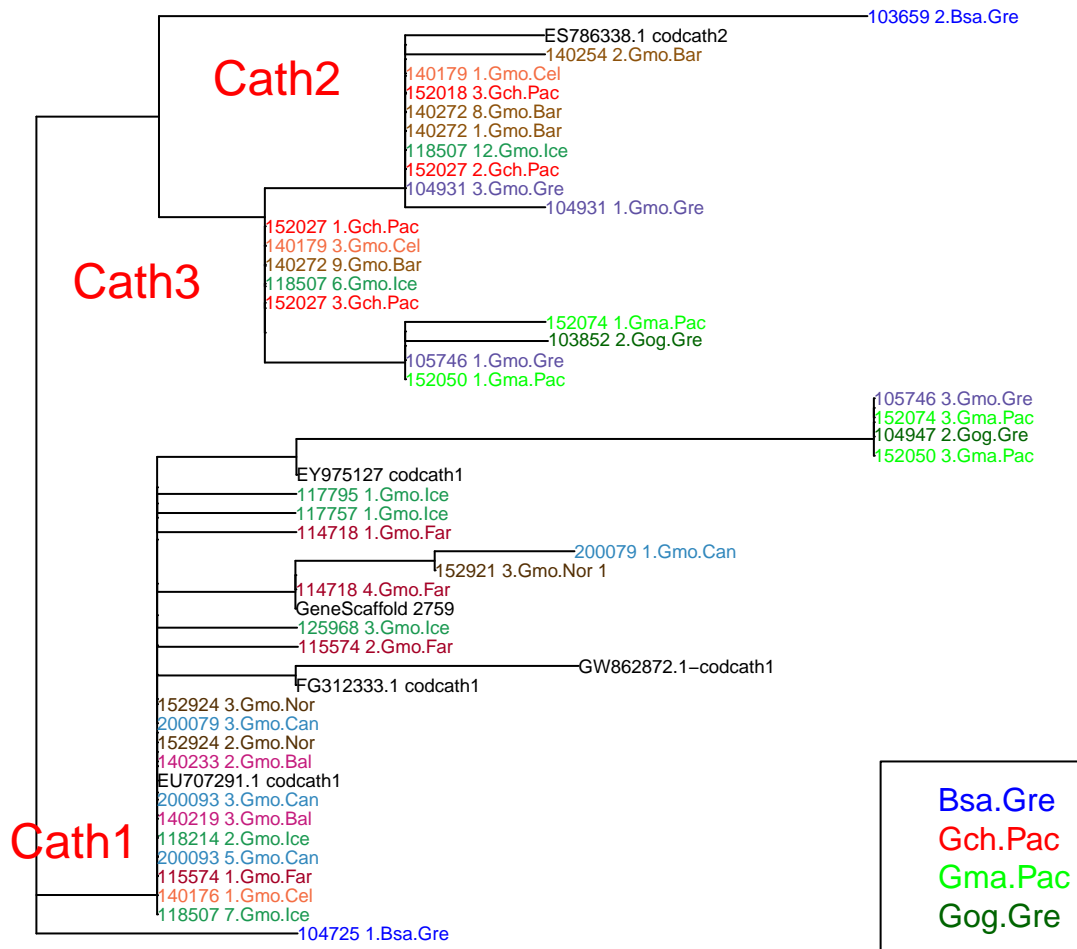

Supplement: Figure S3 — Phylogenetic tree built on amino acid sequences in exons 1, 2, and 3 combined, the conserved part of cathelicidin, of clones from various individuals of Atlantic cod and four closely related taxa. Bsa.Gre (Boreogadus saida), Gch.Pac (Gadus chalcogrammus), Gma.Pac (Gadus macrocephalus), Gog.Gre (Gadus ogac) and Gmo (Gadus morhua) from various locations; Iceland (Gmo.Ice), Greenland (Gmo.Gre), Barents Sea (Gmo.Bar), Celtic Sea (Gmo.Cel), Baltic Sea (Gmo.Bal), Norway (Gmo.Nor), Faeroe Islands (Gmo.Far), Canada (Gmo.Can). [file peerj-03-976-s004.pdf]

Cath3

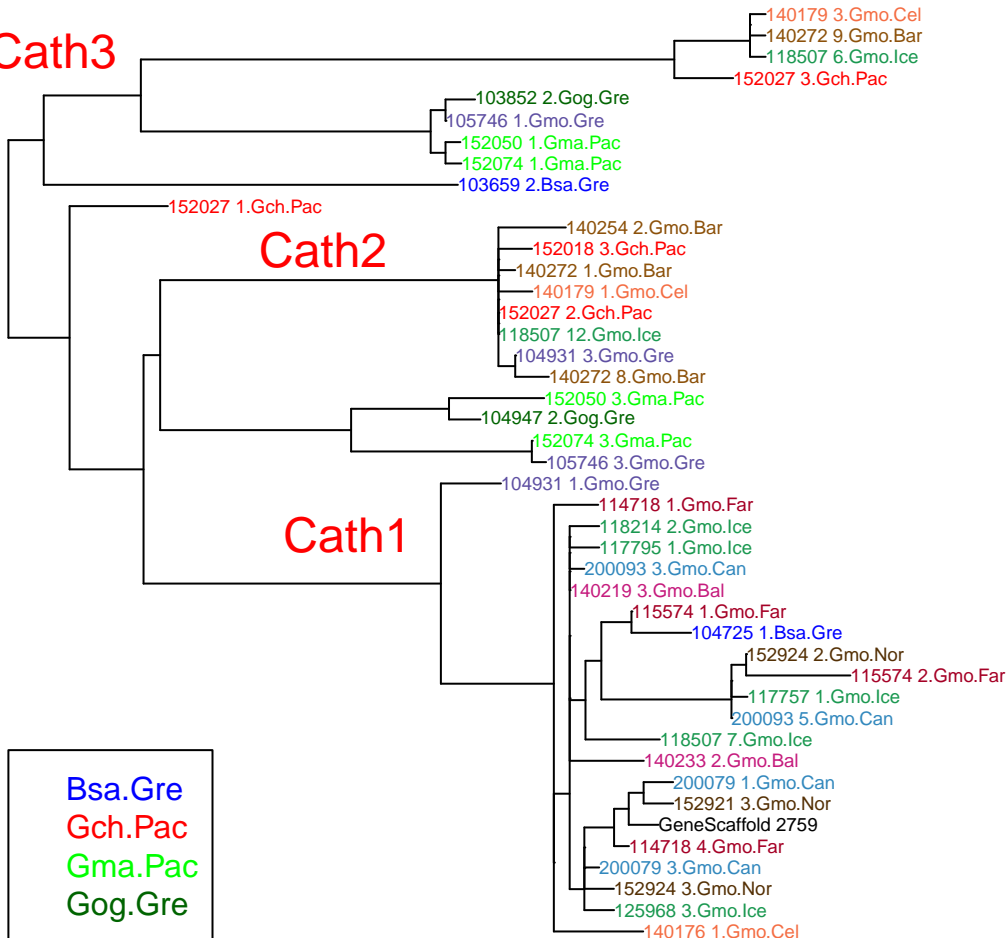

Supplement: Figure S4 — Phylogenetic tree of nucleotide sequences of the cathelicidin gene from 43 representative clones of various individuals of Atlantic cod and four sister taxa. Bsa.Gre (Boreogadus saida), Gch.Pac (Gadus chalcogrammus), Gma.Pac (Gadus macrocephalus), Gog.Gre (Gadus ogac) and Gmo (Gadus morhua) from various locations; Iceland (Gmo.Ice), Greenland (Gmo.Gre), Barents Sea (Gmo.Bar), Celtic Sea (Gmo.Cel), Baltic Sea (Gmo.Bal), Norway (Gmo.Nor), Faeroe Islands (Gmo.Far), Canada (Gmo.Can). [file peerj-03-976-s005.pdf]

Pi and Theta Cath 1

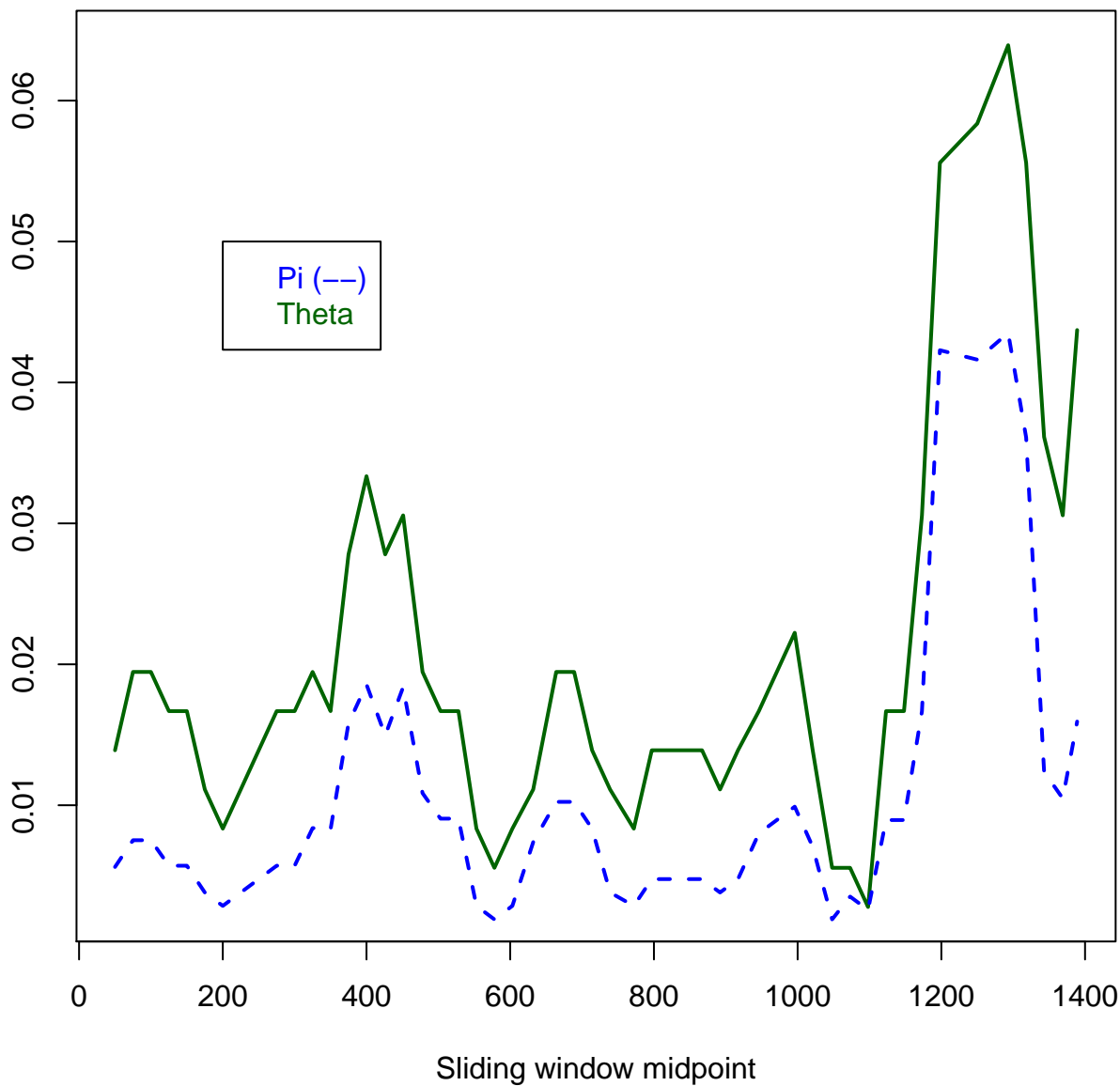

Supplement: Figure S5 — Window length was 100 bp with a 25 bp step size. [file peerj-03-976-s006.pdf]

Tajima's D Cath 1

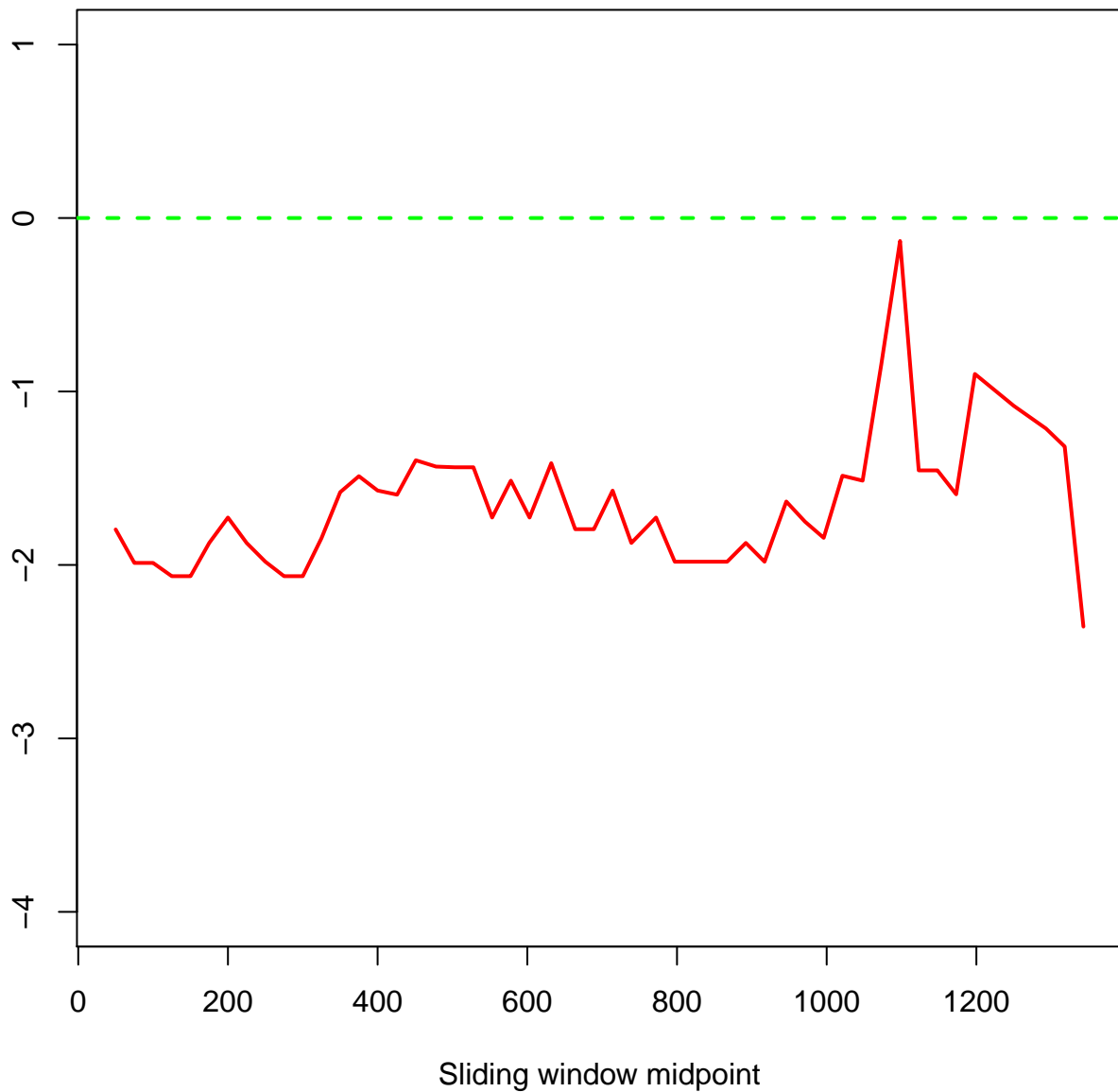

Supplement: Figure S6 — Window length was 100 bp with a 25 bp step size. [file peerj-03-976-s007.pdf]

Pi and Theta Cath 3

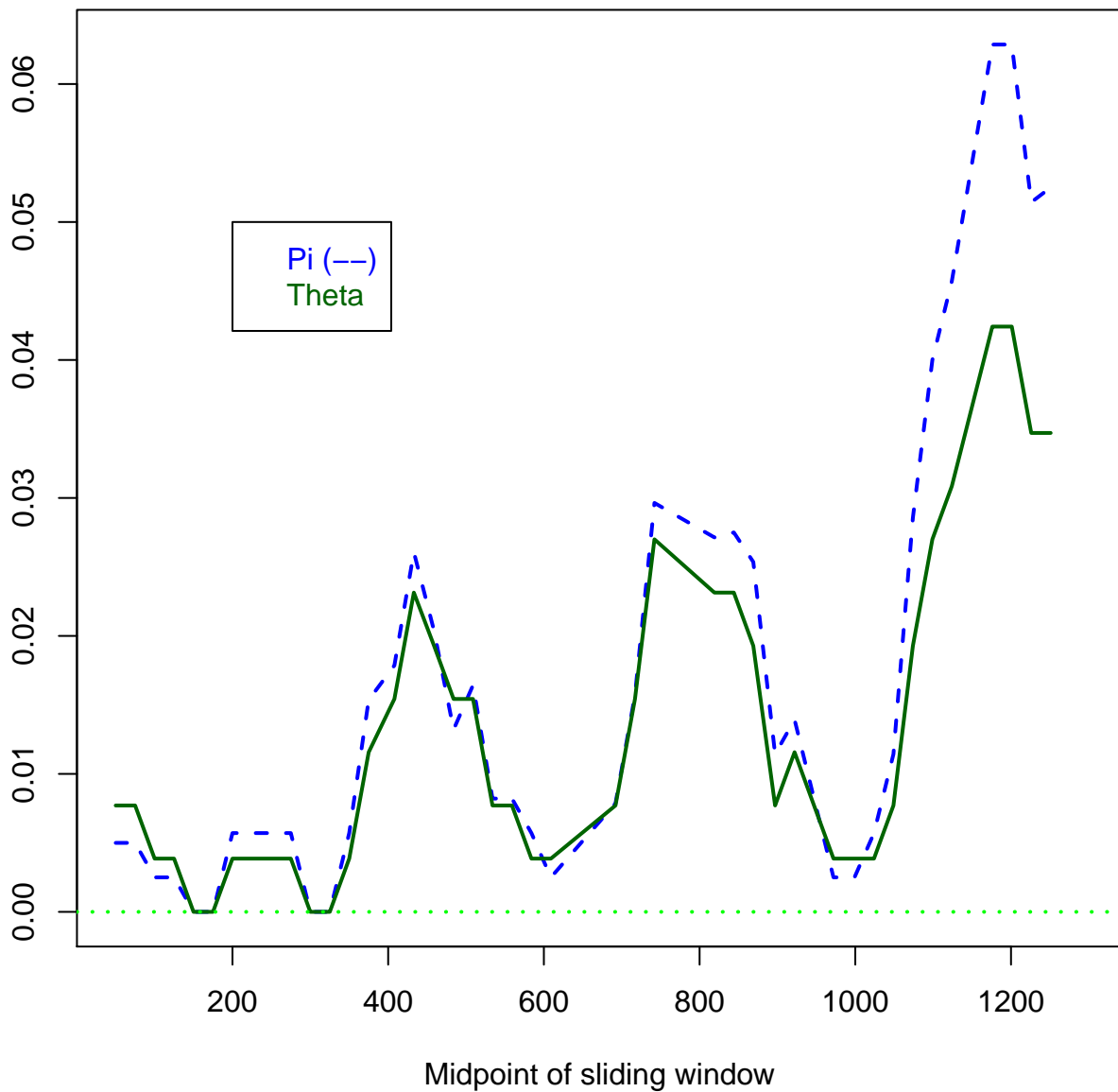

Supplement: Figure S7 — Window length was 100 bp with a 25 bp step size. [file peerj-03-976-s008.pdf]

Tajima's D Cath 3

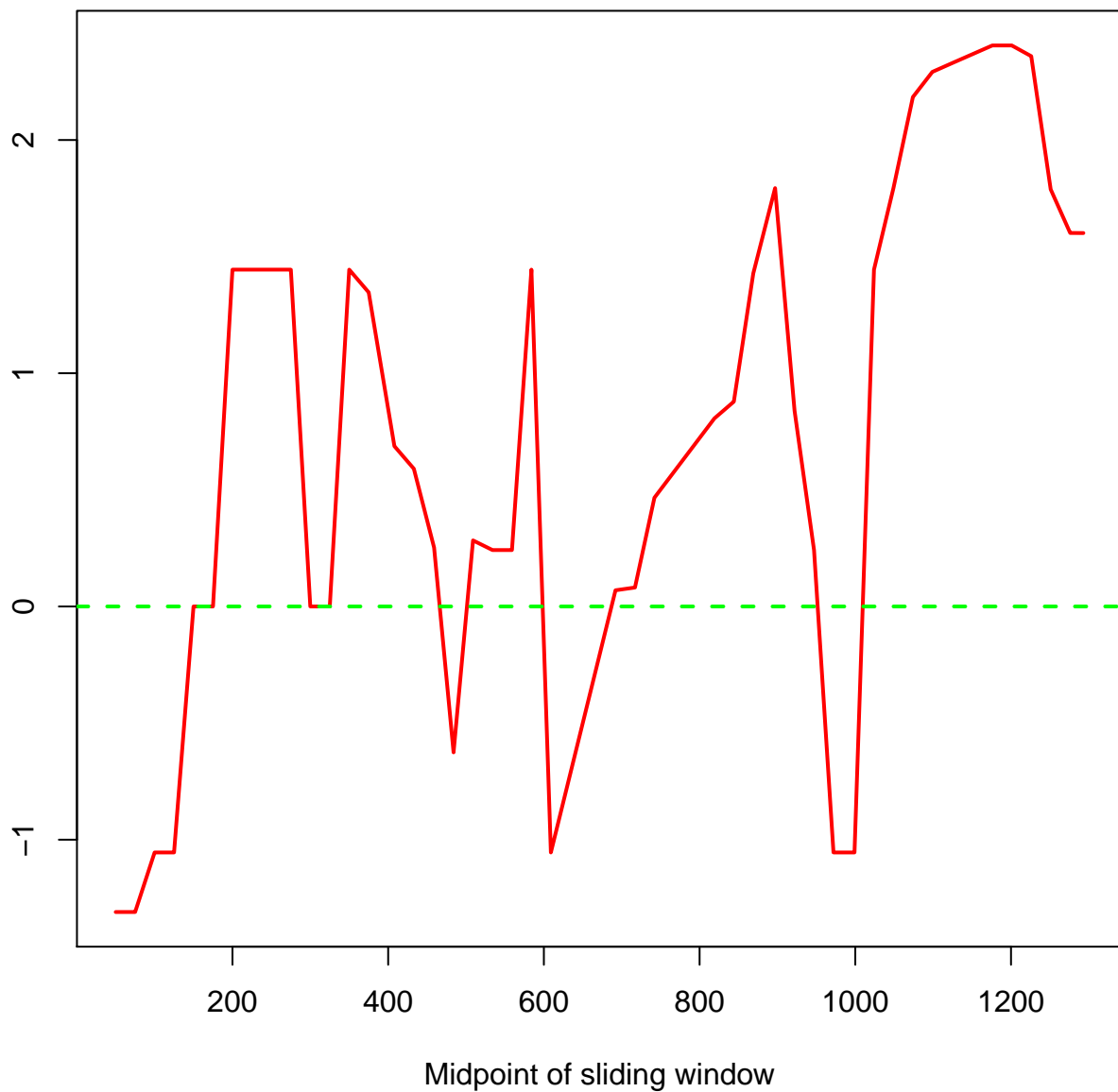

Supplement: Figure S8 — Window length was 100 bp with a 25 bp step size. [file peerj-03-976-s009.pdf]
